# Gut Microbiome and Metabolome Changes in Chronic Low Back Pain Patients With Vertebral Bone Marrow Lesions

**DOI:** 10.1002/jsp2.70042

**Published:** 2025-01-27

**Authors:** Wentian Li, Ji Tu, Jinjian Zheng, Abhirup Das, Qi Yan, Xiaotao Jiang, Wenyuan Ding, Xupeng Bai, Kaitao Lai, Sidong Yang, Cao Yang, Jun Zou, Ashish D. Diwan, Zhaomin Zheng

**Affiliations:** ^1^ Spine Labs, St. George and Sutherland Clinical School University of new South Wales Kogarah Australia; ^2^ Gulbali Institute, School of Agricultural, Environmental and Veterinary Sciences Charles Sturt University Wagga Wagga Australia; ^3^ Nepean Hospital Nepean Blue Mountains Local Health District Penrith Australia; ^4^ Department of Spine Surgery, the First Affiliated Hospital Sun Yat‐Sen University Guangzhou China; ^5^ Department of Orthopedic Surgery The First Affiliated Hospital of Soochow University Suzhou China; ^6^ UNSW Microbiome Research Centre, St George and Sutherland Clinical Campuses, School of Clinical Medicine, UNSW Medicine and Health The University of new South Wales Sydney Australia; ^7^ Department of Spinal Surgery The Third Hospital of Hebei Medical University Shijiazhuang China; ^8^ Hebei Joint International Research Centre for Spinal Diseases Center for Innovation & Translational Medicine, the First Affiliated Hospital, Zhejiang University School of Medicine Hangzhou China; ^9^ Zhejiang Provincial Key Laboratory of Pancreatic Disease, the First Affiliated Hospital Zhejiang University School of Medicine Hangzhou China; ^10^ Charles Perkins Centre and School of Medical Sciences University of Sydney Sydney Australia; ^11^ Northcott Neuroscience Laboratory ANZAC Research Institute, Concord Hospital Sydney Australia; ^12^ Tissue Engineering and Microfluidics Laboratory (TE&M) Australian Institute for Bioengineering and Nanotechnology (AIBN), the University of Queensland St Lucia Australia; ^13^ Department of Orthopaedics, Union Hospital, Tongji Medical College Huazhong University of Science and Technology Wuhan China; ^14^ Spine Service Department of Orthopaedic Surgery, St. George Hospital Kogarah Australia

**Keywords:** BM‐MSCs, bone marrow lesions, chronic low back pain, fatty replacement, gut microbiome, serum metabolomics

## Abstract

**Background:**

Chronic low back pain (LBP) is a significant global health concern, often linked to vertebral bone marrow lesions (BML), particularly fatty replacement (FR). This study aims to explore the relationship between the gut microbiome, serum metabolome, and FR in chronic LBP patients.

**Methods:**

Serum metabolomic profiling and gut microbiome analysis were conducted in chronic LBP patients with and without FR (LBP + FR, *n* = 40; LBP, *n* = 40) and Healthy Controls (HC, *n* = 31). The study investigates alterations in branched‐chain amino acids (BCAAs) levels and identifies key microbial species associated with BCAA metabolism. In vitro experiments elucidate the role of BCAAs in adipogenesis of bone marrow mesenchymal stem cells (BM‐MSCs) via the SIRT4 pathway.

**Results:**

Chronic LBP patients with FR exhibit depleted BCAA levels in their serum metabolome, along with alterations in the gut microbiome. Specific microbial species, including *Ruminococcus gnavus* , *Roseburia hominis* , and *Lachnospiraceae bacterium 8 1 57FAA*, are identified as influential in BCAA metabolism and BM‐MSCs metabolism. In vitro experiments demonstrate the ability of BCAAs to induce BM‐MSCs adipogenesis through SIRT4 pathway activation.

**Conclusion:**

This study sheds light on the intricate relationship between the disturbed gut ecosystem, serum metabolites, and FR in chronic LBP. Dysbiosis in the gut microbiome may contribute to altered BCAA degradation, subsequently promoting BM‐MSCs adipogenesis and FR. Understanding these interactions provides insights for targeted therapeutic strategies to mitigate chronic LBP associated with FR by restoring gut microbial balance and modulating serum metabolite profiles.

## Graphical abstract

This study explores the link between the gut microbiome, serum metabolome, and fatty replacement (FR) in chronic low back pain (LBP) patients. Findings reveal that dysbiosis in the gut microbiome and altered branched‐chain amino acid (BCAA) levels contribute to FR through the SIRT4 pathway, suggesting potential therapeutic strategies targeting gut microbial balance and serum metabolites to alleviate chronic LBP.

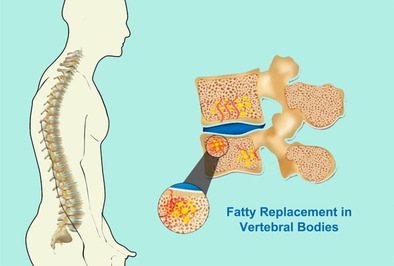

## 1 Introduction

Chronic low back pain (LBP) is a common and debilitating condition worldwide with the Lancet group calling for action [1]. Although chronic LBP relates to different spinal pathologies, vertebral bone marrow lesions (BML) on magnetic resonance imaging (MRI) have a high specificity for discogenic LBP [2]. Vertebral BML are pathological changes of the bone marrow composition in vertebral bodies. A meta‐analysis by Jensen et al. established that the prevalence of BML and the association with chronic LBP estimated the median prevalence of LBP types in symptomatic populations at 43% compared to only 6% in non‐symptomatic populations [3]. Fatty replacement (FR) of normal bone marrow is the most frequent in vertebral BML appearances accounting for up to 90% of BML's observed [4, 5]. Currently, the pathogenesis of vertebral BML remains unclear. FR happens in the vertebral bodies, specifically, in the bone marrow adjacent to the endplate. (Figure S1).

The microenvironment of bone marrow is filled with bone marrow mesenchymal stem cells (BM‐MSCs). BM‐MSCs are multipotent stem cells, and they can differentiate into several mature cells, such as adipocytes and osteoblasts. As common progenitor cells of adipocytes and osteoblasts, BM‐MSCs have delicately balanced the differentiation of osteogenesis and adipogenesis in the bone marrow [6]. Chronic LBP with FR were visualized as fat replacement in vertebral bone marrow on MRI [7]. Metabolism is vitally significant in BM‐MSCs' fate determination and differentiation [8]. Thus, we hypothesize that chronic LBP with FR result from dysfunction of BM‐MSC differentiation regulated by cellular metabolism.

Our understanding of the microbiome that lives in our gut, its functions, and its roles in human health and disease has advanced significantly over the last decade, aided by rapid technological advancement. Gut‐bone axis was proposed to play a significant role in the onset of several bone‐joint diseases such as osteoporosis, rheumatoid arthritis, and spinal cord multiple sclerosis [9]. Previous studies indicated that gut microbiome communicated with bone marrow, regulating disease pathogenesis [10]. As the FR are alterations in the bone marrow milieu in patients with chronic LBP, such as increased bone marrow adiposity, whether the gut microbiome impacts these alterations is still unknown.

Longstreth and Yao drew attention to an excess of back surgery in patients with irritable bowel syndrome (IBS) [11, 12]. It has been shown that back pain is associated with altered gut microbiota composition [13]. Recent research showed that LBP with BML could be caused by one specific microbiota (formerly known as *Propionibacterium acnes* , *P. acnes* ) [14]. This type of bacteria is an aerotolerant gram‐positive and a common skin commensal. Several animal models have found *P. acnes* could exist in the degenerated discs and cause BML [15]. Considering the close relationship between LBP and BML, it is rational to hypothesize the potential roles of human microbiome in BML. However, thus far, no research has been done to detect whether the disturbance of the gut microbiome accompanies the occurrence of BML.

There might be three potential mechanisms by which the gut microbiota could induce intervertebral disc (IVD) degeneration and cause chronic LBP with BML: (a) bacteria translocate across the gut epithelial barrier and arrive at IVDs or bone marrow. IVDs provide an excellent environment for the anaerobic bacteria growth because of the low oxygen tension and the absence of the immune surveillance; (b) translocation of the bacteria could regulate the mucosal and systemic immune systems; (c) the nutrients and metabolites formatted in the gut epithelium diffuse into the IVDs or bone marrow, and then cause FR [16].

The human gut microbiota produces numerous metabolites. These metabolites could be accumulated in the bloodstream, where they can have systemic effects on the host [17]. Serum levels of amino acids, most consistently the BCAAs [18], were the most consumed metabolites in BM‐MSCs differentiated into adipocytes. Interestingly, mature adipocytes have been shown to utilize BCAAs for acetyl‐coenzyme A (CoA) production for lipogenesis [19]. However, the correlation between the gut microbiome and serum metabolome in LBP patients is unknown.

Taken together, to bridge the above‐mentioned knowledge gaps, we conducted 16 s rRNA and shotgun metagenomics analysis of fecal samples from 107 chronic LBP patients with or without FR and 31 healthy volunteers to define their composition of gut microbiota. We first combined shotgun metagenomics and metabolic analysis to uncover the functional and taxonomic characteristics of the gut microbiome in chronic LBP patients with or without FR and healthy volunteers. Thereafter, we evaluated microbiomes' influence on the capabilities of BM‐MSC differentiation. By integrating these multilevel omics results, we defined and characterized the different gut microbiome and metabolites, and their combination and interaction in the gut‐bone marrow ecosystem of chronic LBP with FR.

## 2 Results

### 2.1 Clinical Characteristics of the Participants

We recruited 107 chronic LBP patients and 31 healthy controls (HC). Based on spinal MRI results [20], 107 LBP patients were divided into two groups: 54 LBP patients with BML, referred to as LBP + FR, and 53 LBP patients without BML, referred to as LBP. Hence, we focused on the microbiome analysis of LBP + FR, LBP (LBP without FR) and HC. There was no significant difference in gender, age, body mass index (BMI), total cholesterol levels, and triglycerides levels between the three groups. We found that LBP + FR cohort showed a significantly higher Visual Analog Scale (VAS) and Oswestry Disability Index (ODI) scores compared with the LBP and HC cohort. (Table 1) These well‐matched samples were used to identify molecular signatures inherent in the gut microbiome that modulate host metabolism.

**TABLE 1:** Characteristics of the study population.

	LBP + FR (*n* = 54)	LBP (*n* = 53)	HC (*n* = 31)	*p*
Age (years)	45.67 ± 6.27	45.91 ± 8.72	42.10 ± 9.50	Ns
Gender(F/M)	26/28	29/24	13/18	Ns
BMI, mean (Kg/m^2^)	22.20 ± 5.01	23.83 ± 2.44	23.10 ± 2.10	Ns
VAS (1–10)	5.31 ± 1.84	4.43 ± 2.03	~	0.02
ODI (%)	46.2% ± 19.1%	38.5% ± 17.7%	~	0.03
Total cholesterol	4.55 ± 1.08	4.42 ± 1.04	4.47 ± 0.71	Ns
Level (mmol/L)				
Total triglycerides level(mmol/L)	1.40 ± 0.72	1.43 ± 0.89	1.45 ± 0.72	Ns
Diet	Ad libitum diet	Ad libitum diet	Ad libitum diet	

*Note:* Values are presented as mean ± standard deviation.

Abbreviations: BMI = body mass index; HC = healthy controls; LBP + FR = low back pain with fatty replacement; LBP = low back pain without fatty replacement; ODI = Oswestry Disability Index; VAS = Visual Analog Scale for pain.

### 2.2 Fecal Microbiome Taxonomic Indicators of LBP With FR Using 16S rRNA Gene Sequencing

**FIGURE 1:**
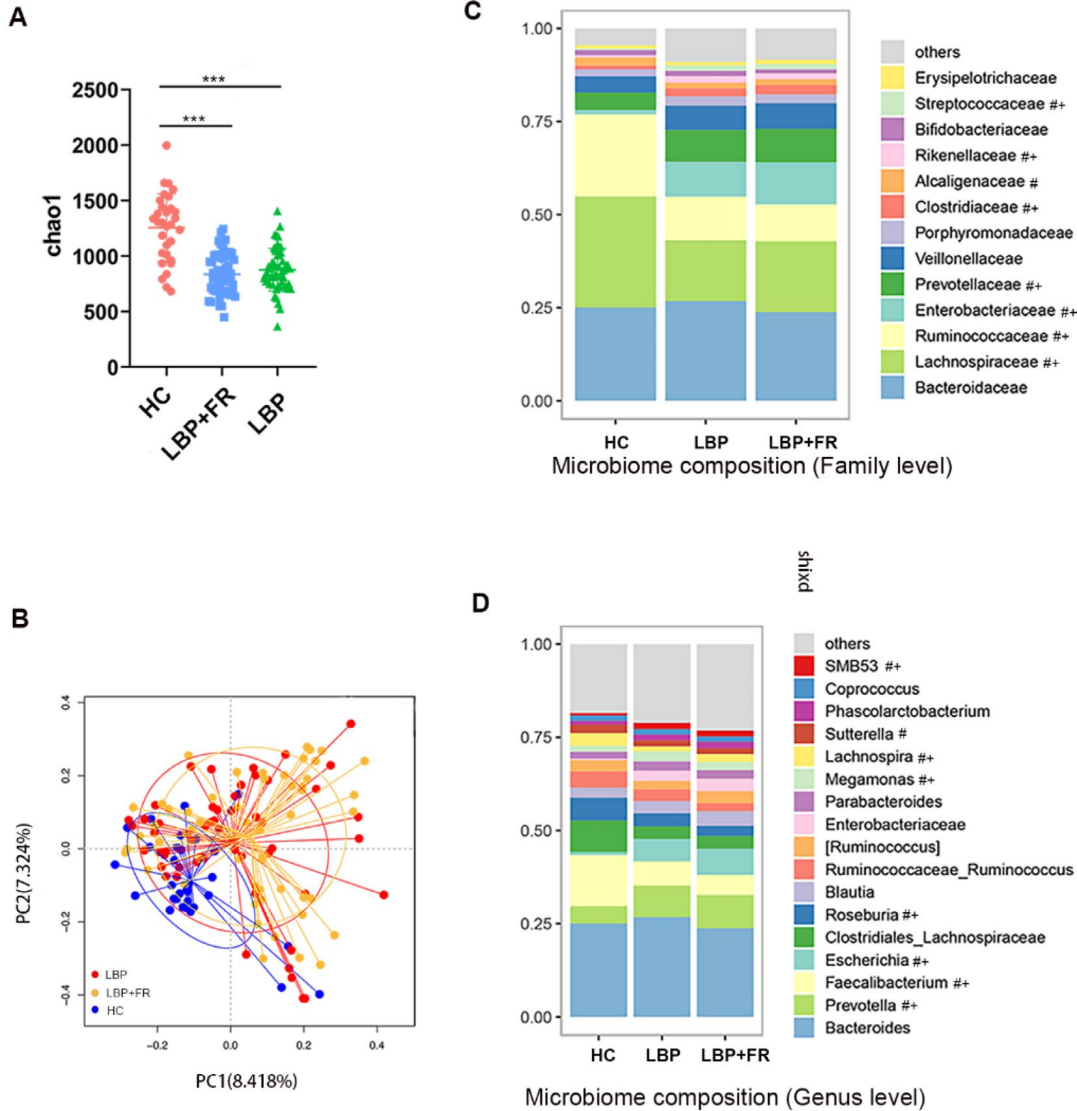
Distinct fecal microbiota profiles of subjects with LBP + FR, LBP and HC by using 16S rRNA gene amplicon sequencing. (A) Comparison of alpha‐diversity indices (Chao‐1‐richness index) between HC, LBP + FR and LBP groups. (B) Principal coordinate analysis (PCoA) based on Bray Curtis distances demonstrating the separation of HC, LBP + FR and LBP groups. (C) Microbiome composition at family level in HC, LBP + FR and LBP fecal samples. (D) Microbiome composition at genus level showing the top 17 most abundant genus HC, LBP + FR and LBP fecal samples. Data in C, D are presented as mean relative abundance, with differences between groups shown as #*p* < 0.05 for LBP + FR compared to HC control, and + *p* < 0.05 for LBP compared to HC control, with exact *p* values shown in Table S2.

To compare the gut bacterial community composition between LBP with FA, LBP patients and healthy controls, we first performed 16S rRNA sequencing for all 138 fecal samples. 16S rRNA sequencing confirmed a dysbiosis in LBP + FR and LBP compared to HC (Figure 1A,B). Initially, alpha‐diversity analysis (alpha‐diversity analysis measures the variety and number of different types of bacteria present in the gut) of gut microbiome indicated samples from LBP + FR and LBP had decreased alpha‐diversity index: Chao‐1‐richness index (Figure 1A), Observed‐otus, Shannon's diversity index and PD‐whole‐tree index (Figure S2A–C), compared with samples from HC group. Besides, bacterial alpha‐diversity analysis showed that there was no significant difference among these indexes between LBP + FR and LBP groups.

**FIGURE 2:**
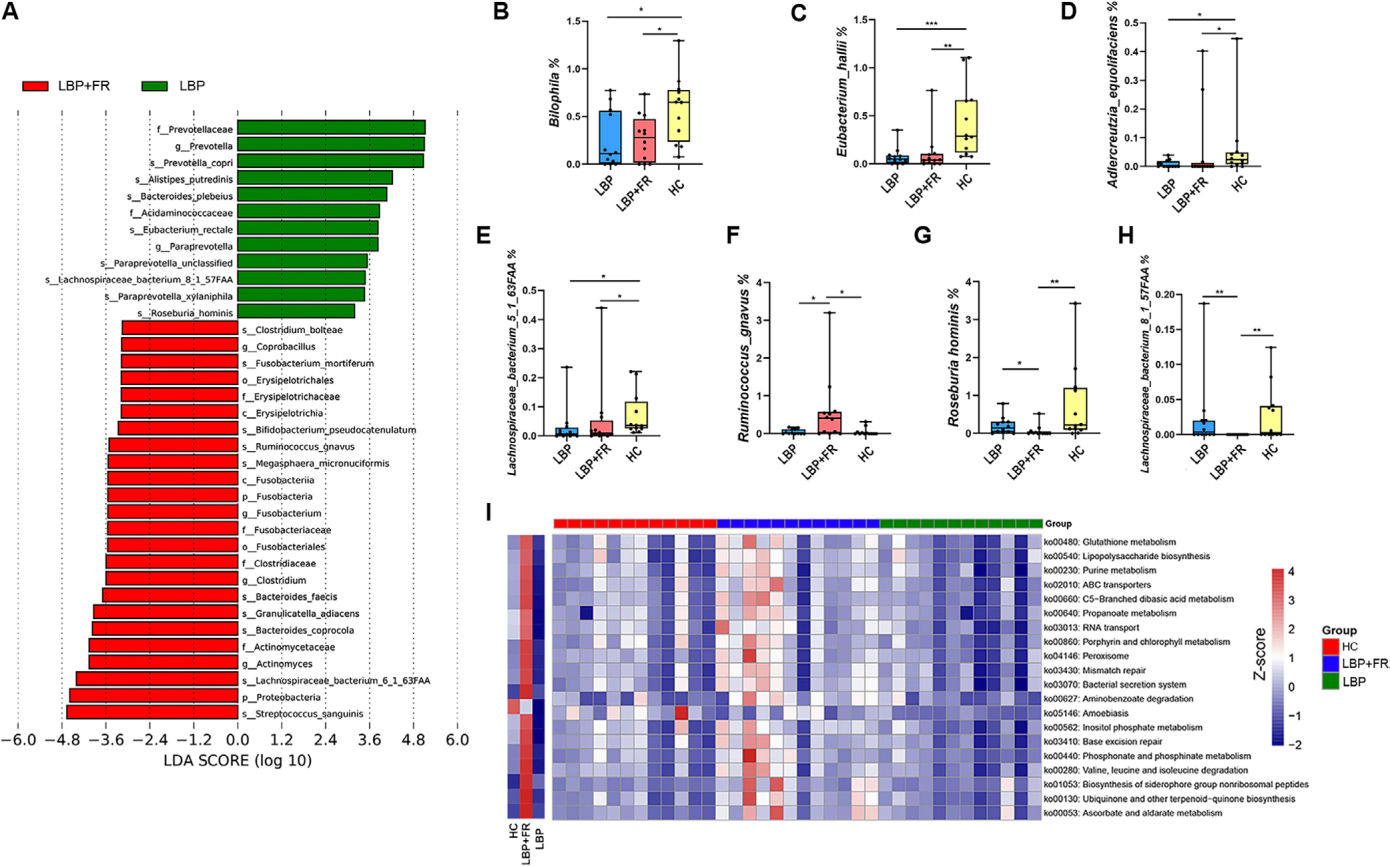
The fecal microbiota of LBP + FR patients can be distinguished from that of LBP and HC individuals in metagenomic analysis. (A) Linear discriminant analysis effect size identified the most differentially abundant taxa between the LBP + FR and LBP groups. LBP‐enriched taxa are indicated with a positive LDA score, and taxa enriched in LBP + FR groups have a negative score. Only taxa meeting an LDA significant threshold of > 2 are shown. (B–H) The relative abundances at species level of *Bilophila, Eubacterium hallii, Adlercreutzia equolifaciens, Lachnospiraceae bacterium 5 1 63FAA, Ruminococcus gnavus, Roseburia hominis*, and *L. bacterium 8 1 57FAA* between LBP + FR, LBP and HC groups. * indicates *p* < 0.05; ** indicates *p* < 0.01; and *** indicates *p* < 0.001 by Wilcoxon rank‐sum test. (I) Heatmap of functional capacity profiles showed the top 20 enrichments in the LBP + FR group by metagenomic sequencing analysis.

Next, β‐diversity analysis (β‐diversity analysis compares the differences in the types and abundance of bacteria between different gut microbiome samples) was used to explore patients' overall gut microbiome phenotypes between three patient groups. Principal coordinate analysis (PCoA) showed that bacterial signatures between LBP + FR and LBP when compared to HC group were significantly distinct when using the Bray_Curtis distance (*p* = 0.01) (Figure 2B), Unweighted_UniFrac and Weighted_UniFrac distances (Figure S2D,E).

Here, we identified a total of 343 discriminative bacterial species between three groups (Table S1). Compared with HC cohort, LBP + FR and LBP cohorts were characterized by presence of five enriched families (Streptococcaceae, Prevotellaceae, Rikenellaceae, Clostridiaceae, and Enterobacteriaceae) and by three depleted families (Ruminococcaceae, Alcaligenaceae, and Lachnospiraceae) (Figure 1C). No other differences were observed between the LBP with FR and LBP groups at the family level. Further, at the genus level, compared with HC, LBP + FR and LBP were confirmed by four enriched genuses (Prevotella, Escherichia, Megamonas, and SMB53) and four depleted genuses (Faecalibacterium, Roseburia, Lachnospira, and Sutterella) (Figure 1D).

### 2.3 Fecal Microbiome Taxonomic Indicators of LBP With FR Using Metagenomics

Having identified distinct fecal taxa using 16S rRNA gene sequencing, we sought to increase the resolution of these findings via metagenomic sequencing. 16S rRNA sequencing is conducted to identify bacteria, while shotgun metagenomic sequencing is performed to detect the full range of microbial organisms, including bacteria, fungi, viruses, and other microorganisms. Metagenomics surpasses 16S rRNA sequencing in detecting novel or poorly characterized species by analyzing the entire genetic content of a sample, providing a more comprehensive and unbiased view of microbial diversity. Shotgun metagenomics can detect more of the gut microbiota than 16S rRNA gene sequencing. While 16S sequencing only identifies a portion of the microbiota, shotgun sequencing can find more species, especially when there are many reads available. We chose a two‐stage design (16S, first‐stage; metagenomics, second‐stage) in this study which is recommended by previous researchers [21, 22]. 36 samples from 3 groups (LBP + FR, LBP, and HC) (Table S6. Characteristics of the study population in Fecal Metagenomic Sequencing Analysis) were chose for metagenomic sequencing. Alpha diversity analysis showed that there was no significant difference among these indexes (Observed‐otus: counts the number of different species found in a sample; Chao‐1‐richness index: estimates the total number of species in a sample; and Shannon's diversity index: measures the diversity of a sample by considering both the number of species and how evenly they are distributed.) between any two groups (Figure S3A). PCoA based on Jaccard dissimilarity distances (compares how different the species are between two samples based on their presence or absence.) showed a significantly difference between LBP + FR and LBP compared to HC controls (*p* = 0.002) (Figure S3B).

To further identify which bacterial taxa were distinct between the LBP + FR and LBP, we performed the linear discriminant analysis of effect size (LEfSe analysis is a tool that identifies and ranks the bacteria or microbes most responsible for differences between groups, helping researchers find key biomarkers in microbiome studies.) and identified 11 generas showing significant differences at the species level (Figure 2A and Figure S3C–M). The abundance comparisons of predominant genera showed that *Fusobacterium mortiferum, Ruminococcus gnavus, Granulicatella adiacens*, and *Streptococcus sanguinis* were significantly enriched, whereas *Bacteroides coprocola, Prevotella copri, Alistipes putredinis, Bacteroides plebeius, Paraprevotella, Lachnospiraceae bacterium 8 1 57FAA*, and *Roseburia hominis* were depleted in patients with LBP + FR compared to LBP group (Figure S3C–M).

Moreover, examination of the microbiome at the species level identified a consortium of common bacterial species significantly depleted in both LBP + FR and LBP compared to HC groups (Figure 2B–H). These included *Bilophila unclassified, Eubacterium hallii*, *Adlercreutzia equolifaciens*, and *L. bacterium 5 1 63FAA* (Figure 2B–E). Furthermore, three species were found to discriminate LBP + FR from LBP and HC: enriched *R. gnavus* , depleted *R. hominis* , and reduced *L. bacterium 8 1 57FAA* (Figure 2F–H). Enriched *R. gnavus* was also seen at LEfSe analysis between three groups (Figure S3N), thereby confirming that *R. gnavus* is specifically enriched in LBP + FR compared to LBP and HC controls (LDA log_10_ 3.5).

We also compared the functionality of the fecal Microbiome in LBP + FR, LBP and HC groups based on metagenomics sequencing. We confirmed the twenty most abundant KO (KEGG ORTHOLOGY) pathways in LBP + FR group (Figure 2I). The top five pathways were associated with ascorbate and aldarate metabolism (ko00053), ubiquinone and other terpenoid‐quinone biosynthesis (ko00130), biosynthesis of siderophore group nonribosomal peptides (ko01053), valine, leucine, and isoleucine degradation (ko00280) and phosphonate and phosphinate metabolism (ko00440) (Figure 2I and Table S3).

### 2.4 Functional Indicators of the LBP + FR Fecal Metabolome

To find the microbe‐host interaction in chronic LBP with FR, we collected 120 serum samples from 138 participants and tried to discriminate the metabolic profiles between LBP + FR (*n* = 40), LBP (*n* = 40), and HC (*n* = 30) (Table S7. Characteristics of the study population in Fecal Metabolome Analysis). The overwhelming mass of research supports gut microbiome could produce some end products of fermentation, these products may enter our circulation system by blood and cause some influence on our physiology. The serum metabolome can provide the functional readout of the gut microbiome [23].

**FIGURE 3:**
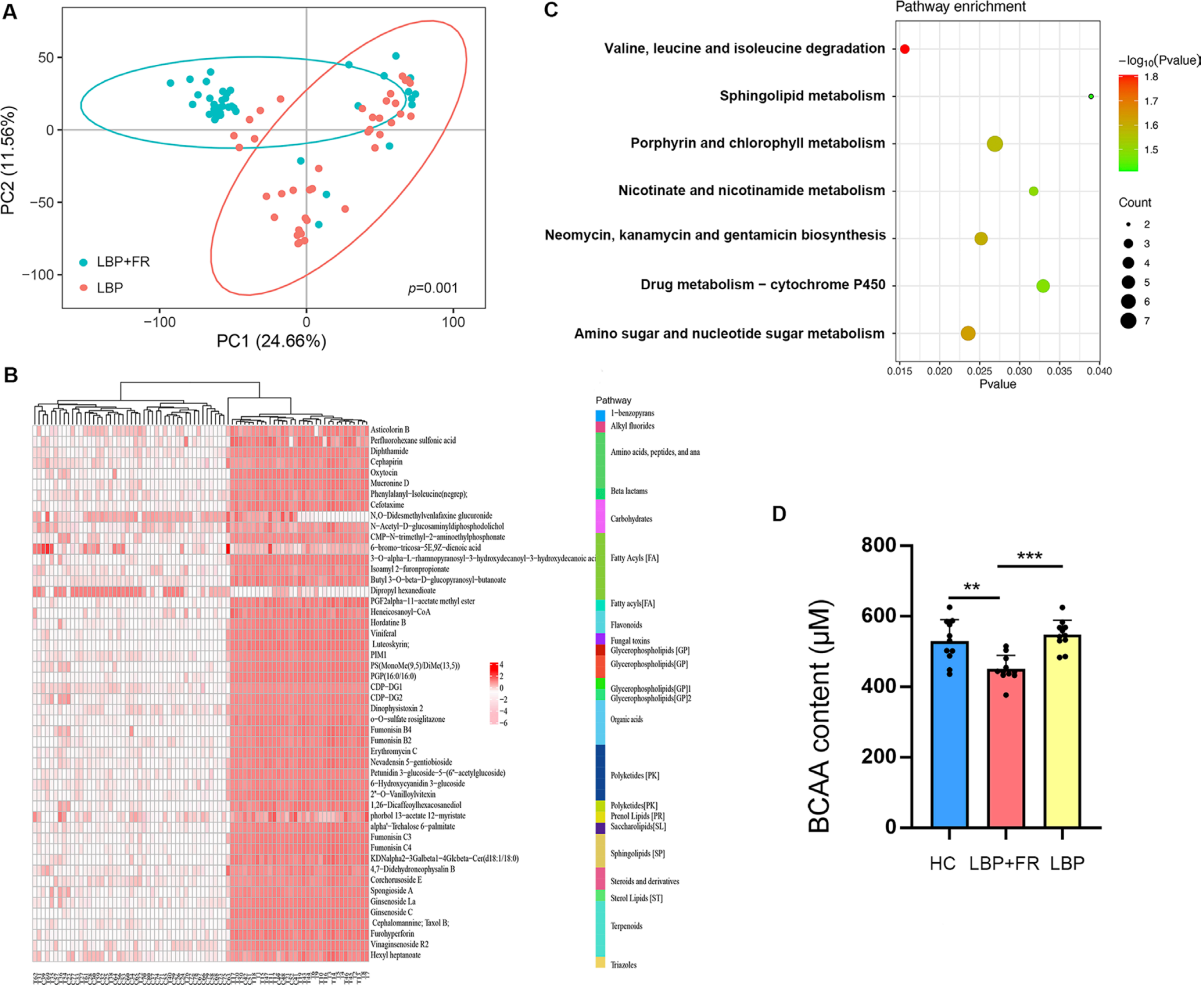
Blood metabolites that discriminate HC, LBP + FR and LBP. (A) Metabolic signatures of LBP + FR subjects were significantly distinguished from LBP (PERMANOVA, *p* = 0.001). Discovery set: LBP + FR, *n* = 40; LBP, *n* = 40. (B) Relative abundances of 50 blood metabolites differentiating between the two groups. Compared with LBP, the LBP + FR group was characterized by 16 up‐regulated and 34 down‐regulated metabolites. These metabolites were mainly involved in amino acid metabolism, carbohydrate metabolism, fatty acyls metabolism, glycerophospholipids metabolism, and sphingolipids metabolism. (C) Metabolomics pathway enrichment analysis identified seven significantly over‐represented sub‐pathways among the differential metabolites. (D) The absolute qualification of BCAAs in serum samples between LBP + FR, LBP and HC groups. * indicates *p* < 0.05; ** indicates *p* < 0.01; and *** indicates *p* < 0.001 by Wilcoxon rank‐sum test.

In this study, principal component analysis (PCA) revealed a significant but incomplete separation of LBP + FR and LBP (PERMANOVA, *p* = 0.001; Figure 3A). Moreover, PCA also revealed incomplete separation between LBP + FR, LBP, and HC (Figure S4). In LBP + FR and LBP groups, we detected a total of 745 biochemicals that differed significantly with VIP > 1. Compared with the LBP group, the LBP + FR group showed depletion in 185 metabolites and enrichment in 560 metabolites. Of the top 50 indicator metabolites with highest variable importance in projection (VIP) score separating LBP + FR from LBP samples, these altered metabolites were mainly involved in Amino acid metabolism, Carbohydrate metabolism, Fatty Acyls metabolism, Glycerophospholipids metabolism, and Sphingolipids metabolism. (Figure 3B).

Furthermore, we conducted a metabolic pathway analysis to help us understand the major biochemical metabolic pathways and signal transduction pathways involved in metabolites based on the KEGG database. The result showed that patients with LBP + FR were mainly characterized by disturbances of valine, leucine, and isoleucine degradation (BCAAs degradation), Sphingolipid metabolism, Porphyrin and chlorophyll metabolism, Nicotinate and nicotinamide metabolism, Neomycin, kanamycin and gentamicin biosynthesis, Drug metabolism—cytochrome P450, Amino sugar and nucleotide sugar metabolism. (Figure 3C and Table S4) Among these metabolisms, BCAAs degradation was the most significant (*p* = 0.0156). Then, we measured the absolute qualification of BCAAs in serum samples. The level of BCAA in LBP + FR was decreased significantly compared with LBP and HC groups. (Figure 3D) Integration of these findings showed that disturbance of BCAAs metabolism was of particular relevance to LBP + FR.

### 2.5 Co‐Occurrence Analysis Among the Gut Microbiome and Metabolites

**FIGURE 4:**
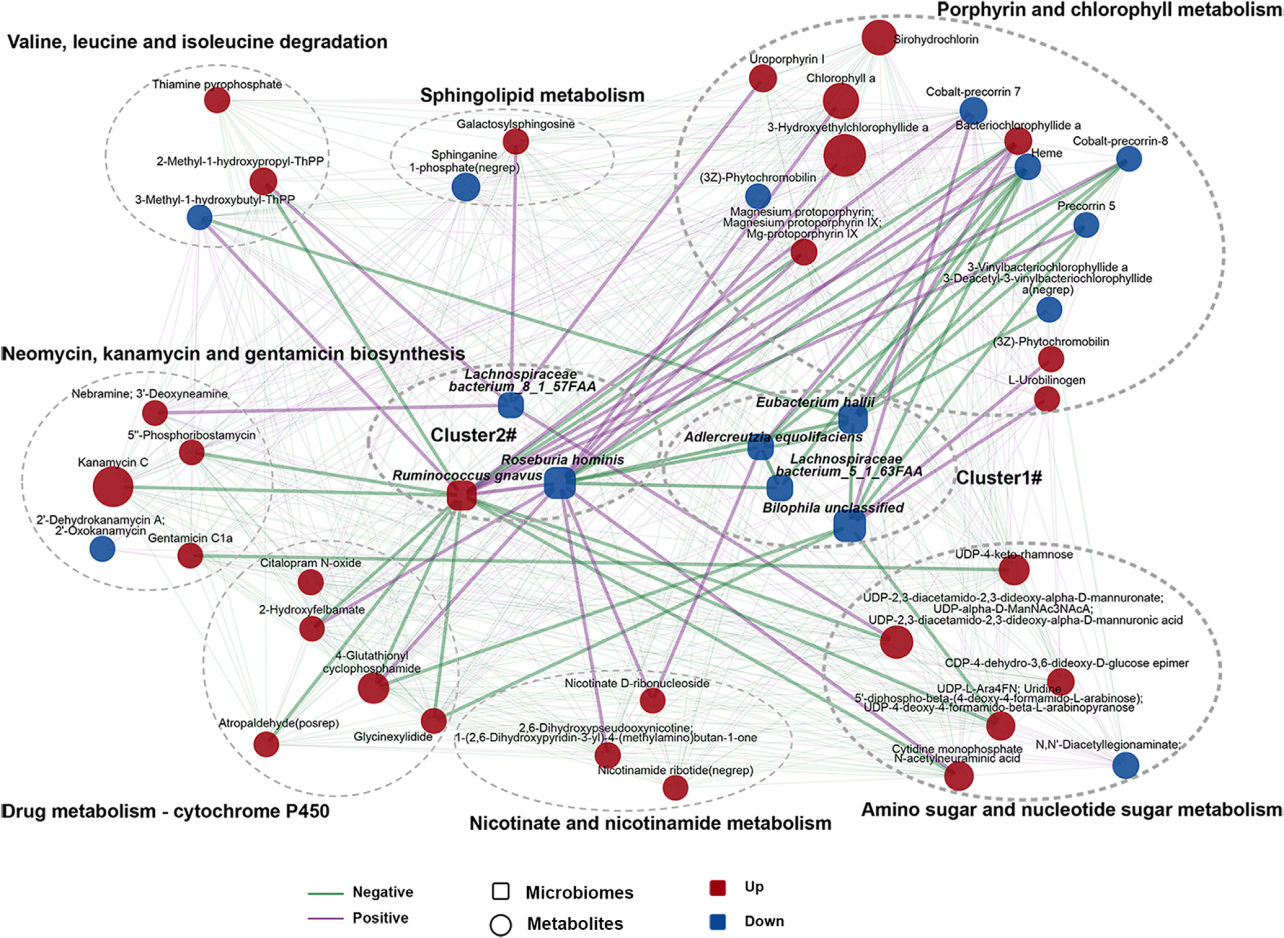
A co‐occurrence network constructed from the relative abundances of differential bacteria, and serum metabolites in LBP + FR/LBP subjects versus HCs.

To explore the potential relationships of abundances of different microbiome species and serum metabolites, co‐occurrence analysis showed that the bacterial species formed strong and broad co‐occurring relationships with serum metabolites. (Figure 4).

Within this co‐expression network, these differential bacterial species were separated from two clusters (Clusters 1 and 2). In the LBP with or without FR group relative to the HC group, Cluster 1 was composed of four depleted bacterial species; (*Bilophila unclassified, E. hallii, A. equolifaciens*, and *L. bacterium 5 1 63FAA*). Cluster 2 mainly included three species ( *R. gnavus* , *R. hominis* , and *L. bacterium 8 1 57FAA*) to distinguish LBP + FR group from LBP and HC group. Within Cluster 1, *Bilophila unclassified, E. hallii, A. equolifaciens*, and *L. bacterium 5 1 63FAA* showed negative correlation with each other, except for the connection between *Bilophila unclassified* and *L. bacterium 5 1 63FAA*. Clusters 1 and 2 were linked by a common node ( *R. hominis* ). In Cluster 2, *R. hominis* positively correlated with *R. gnavus* . Besides, some members from Cluster 1 (*E. hallii, A. equolifaciens*, and *L. bacterium 5 1 63FAA*) showed negative correlations with the members from Cluster 2 ( *R. hominis* ). These findings suggest that those important differential bacterial species may form a synergistic relationship in patients with LBP, LBP + FR, and HC.

Regarding the correlation between microbiome and metabolites, the first network indicated associations between Cluster 1 and a group of 14 metabolites in Porphyrin and chlorophyll metabolism. In our multivariate analysis, each of these 14 metabolites was identified as different between LBP (with/without FR) and HC group. The second network showed the connection between Cluster 2 and numerous metabolites from 7 enriched pathways (valine, leucine and isoleucine degradation, Sphingolipid metabolism, Porphyrin and chlorophyll metabolism, Nicotinate and nicotinamide metabolism, Neomycin, kanamycin and gentamicin biosynthesis, Drug metabolism—cytochrome P450, and Amino sugar and nucleotide sugar metabolism) (Figure 3C). Significantly, Porphyrin and chlorophyll metabolism showed a strong correlation with both the Cluster 1 species and Cluster 2 species, which helps explain the close relationship of this pathway between LBP and related species. Moreover, the metabolites from the most significantly enriched pathway: valine, leucine, and isoleucine degradation (Figure 3C) showed a positive correlation with *R. gnavus* and *L. bacterium 8 1 57FAA*, and a negative connection with *R. gnavus* . Meanwhile, in Cluster 2, *R. gnavus* showed a more negative relationship with other metabolites, but *R. hominis* and *L. bacterium 8 1 57FAA* present more positive connection with other metabolites. It suggested the different functions of these three species in development of FR. Although our study found a potential interaction of gut microbiome with metabolites in LBP + FR, it remains to be further determined whether these metabolic products can influence the LBP + FR pathogenesis directly.

The discriminating bacterial species are mainly separated into two clusters. Cluster 1 was composed of 4 depleted species (*Bilophila unclassified, E. hallii, A. equolifaciens*, and *L. bacterium 5 1 63FAA*) in LBP (with/without FR) subjects compared to HC group. Cluster 2 comprised three species (increased *R. gnavus* , reduced *R. hominis* and decreased *L. bacterium 8 1 57FAA*). Within Cluster 1, *Bilophila unclassified*, *E. hallii, A. equolifaciens*, and *L. bacterium 5 1 63FAA* showed negative correlation with each other, except the connection between *Bilophila unclassified* and *L. bacterium 5 1 63FAA*. In Cluster 2, *R. gnavus* positively correlated with *R. hominis* . Besides, some members from Cluster 1 (*E. hallii, A. equolifaciens*, and *L. bacterium 5 1 63FAA*) showed negative correlations with the members from Cluster 2 ( *R. hominis* ).

In this network, altered metabolites in Clusters 1 and 2 were mainly involved in Porphyrin and chlorophyll metabolism. Valine, leucine and isoleucine degradation showed a positive correlation with *R. gnavus* and *L. bacterium 8 1 57FAA*, and a negative connection with *R. gnavus* . The size of the nodes represents the abundance of these variables. Red and blue dots indicate the increased and decreased relative abundances of variables in LBP + FR/LBP subject relative to HC, respectively. Edges between nodes indicate Spearman's negative (light green) or positive (light purse) correlation.

### 2.6 BM‐MSCs From Patients With LBP + FR Exhibit a Strong Adipogenesis Capability

After characterizing the gut microbiome composition and related metabolomics in LBP + FR and LBP, we tried to detect the effects of the gut microbiota on the BM‐MSCs' function in an ex vivo cell culture. We isolated in vitro BM‐MSCs from LBP + FR patients and LBP patients. Firstly, BM‐MSCs were immune‐phenotypically characterized by flow‐cytometry for the expression of mesenchymal, hematopoietic, and neuronal markers. BM‐MSCs were positive for CD105 and CD29, negative for CD34 and CD45 (Figure S5).

**FIGURE 5:**
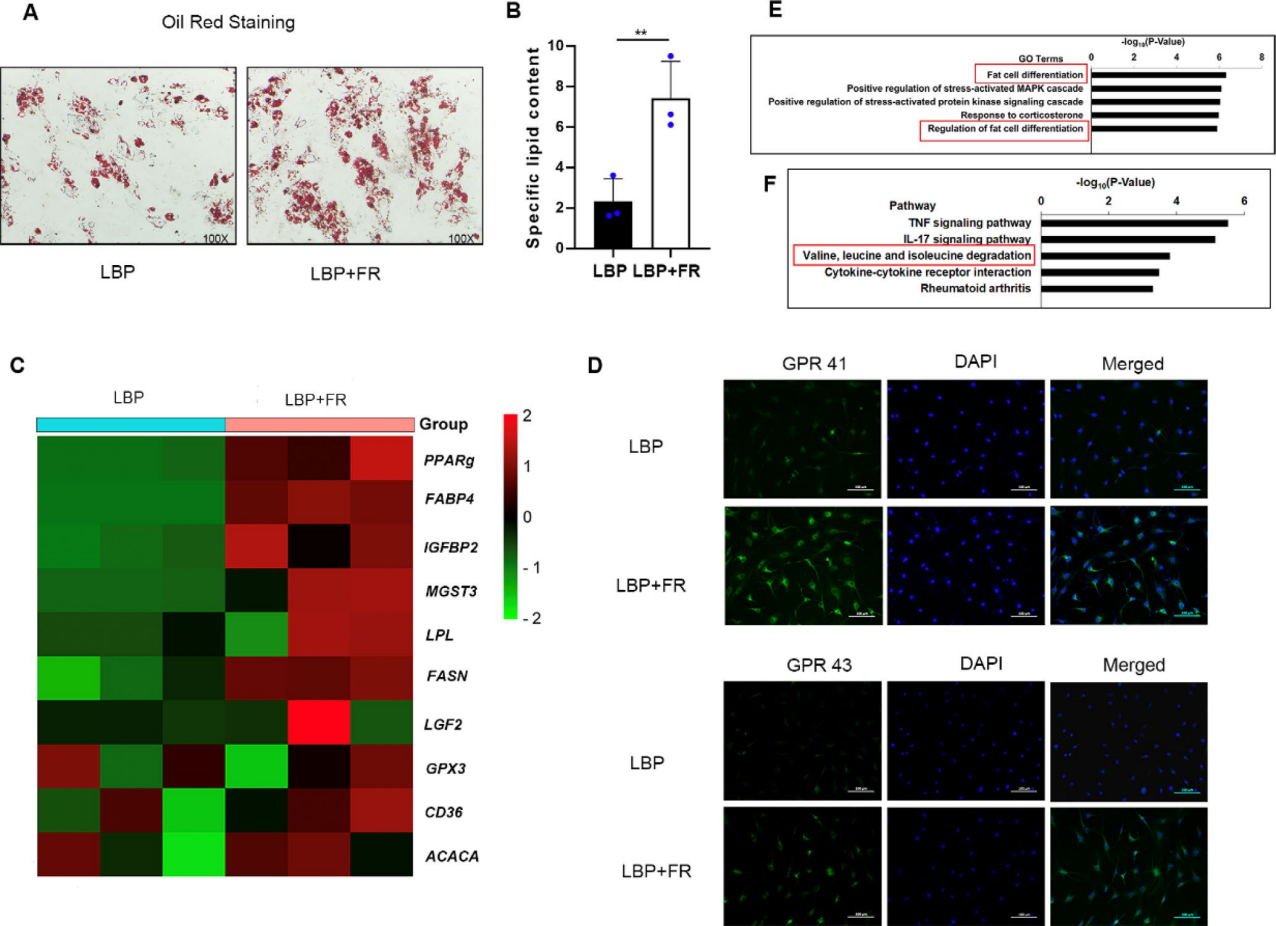
BM‐MSCs from LBP patients with FR exhibit a strong differentiation capability. (A) Representative images of Oil Red O staining of lipids and (B) quantification of the number of oil spots in BM‐MSCs cultured in adipogenic induction medium for 14 days. 100× magnification. * indicates *p* < 0.05; ** indicates *p* < 0.01; and *** indicates *p* < 0.001 by Wilcoxon rank‐sum test. (C) qRT‐PCR analysis results of dysregulated adipogenic genes in LBP + FR and LBP derived BM‐MSCs cultured in adipogenesis induction medium for 48 h. (D) Immunocytochemistry of G protein‐coupled receptor 41 (GPR41) and G‐protein‐coupled receptor 43 (GPR43) in BM‐MSC. Scale bar = 100 μm. (E, F) Gene Ontology (GO) and Kyoto Encyclopedia of Genes and Genomes (KEGG) pathway enrichment analysis in LBP + FR (*n* = 6) and LBP groups (*n* = 6).

To examine the BM‐MSCs' differentiated potential, BM‐MSCs were cultured in adipogenesis medium for 14 days. Oil Red staining showed that BM‐MSCs from LBP + FR facilitated lipid droplet formation (Figure 5A,B). Besides, RT‐qPCR also confirmed the mRNA expression of adipogenic genes in BM‐MSCs' adipogenesis differentiation. The expression of peroxisome proliferator–activated receptor‐γ (Ppar‐gama/PPARg) and fatty acid binding protein 4 (FABP4), 2 key markers of adipocyte differentiation, including other adipogenic differentiation markers (IGFBP2, MGST3, LPL, and FASN) in BM‐MSCs from LBP + FR patients, were higher than the BM‐MSCs from LBP patients (Figure 5C).

Besides, we found the fatty acids related to cell receptor G‐protein‐coupled receptor 41 (GPR41) also called free fatty acid receptor 3 (FFAR3) and G‐protein‐coupled receptor 43 (GPR43/FFAR2) is a Gαi‐coupled receptor activated by SCFAs mainly produced from dietary complex carbohydrate fibers in the large intestine as products of fermentation by microbiota. This result showed the BM‐MSCs from LBP + FR patients had a stronger GPR41/43 immunostaining, which means BM‐MSCs from LBP + FR patients were easily influenced by fatty acids. (Figure 5D).

To further confirm the obtained results on the transcriptomic scale, whole transcriptome RNA sequencing was performed for the cells cultured in adipogenesis media solutions. Using gene ontology (GO) enrichment analysis, we observed that there were some enrichments in GO terms associated with fat cell differentiation and regulation of fat cell differentiation, this result illustrated that the BM‐MSCs from LBP + FR patients have a promoted initiation of adipogenesis. (Figure 5E) Furthermore, a KEGG pathway analysis in the upregulated gene group was also carried out. The top five related pathways of upregulated genes for the two groups are shown in Figure 5F, including: TNF signaling pathway, IL‐17 signaling pathway, valine, leucine, and isoleucine degradation, Cytokine‐cytokine receptor interaction and Rheumatoid arthritis. It should be noted that the pathway about valine, leucine and isoleucine degradation was mentioned again, this pathway has been confirmed to have a close relationship with gut microbiome and serum metabolome in LBP + FR patients. Detailed results of GO and KEGG enrichment analysis are summarized in Table S5.

All these results show that the BM‐MSCs' adipogenesis capability increases in LBP patients with FR. These results kept consistent with the MRI pathology characteristics and indicated that BM‐MSCs' differentiation ability had a close relationship with the FR process. The BCAA degradation pathway was indicated to affect the development of FR positively.

### 2.7 BCAA Degradation Promotes BM‐MSC's Adipogenic Differentiation

**FIGURE 6:**
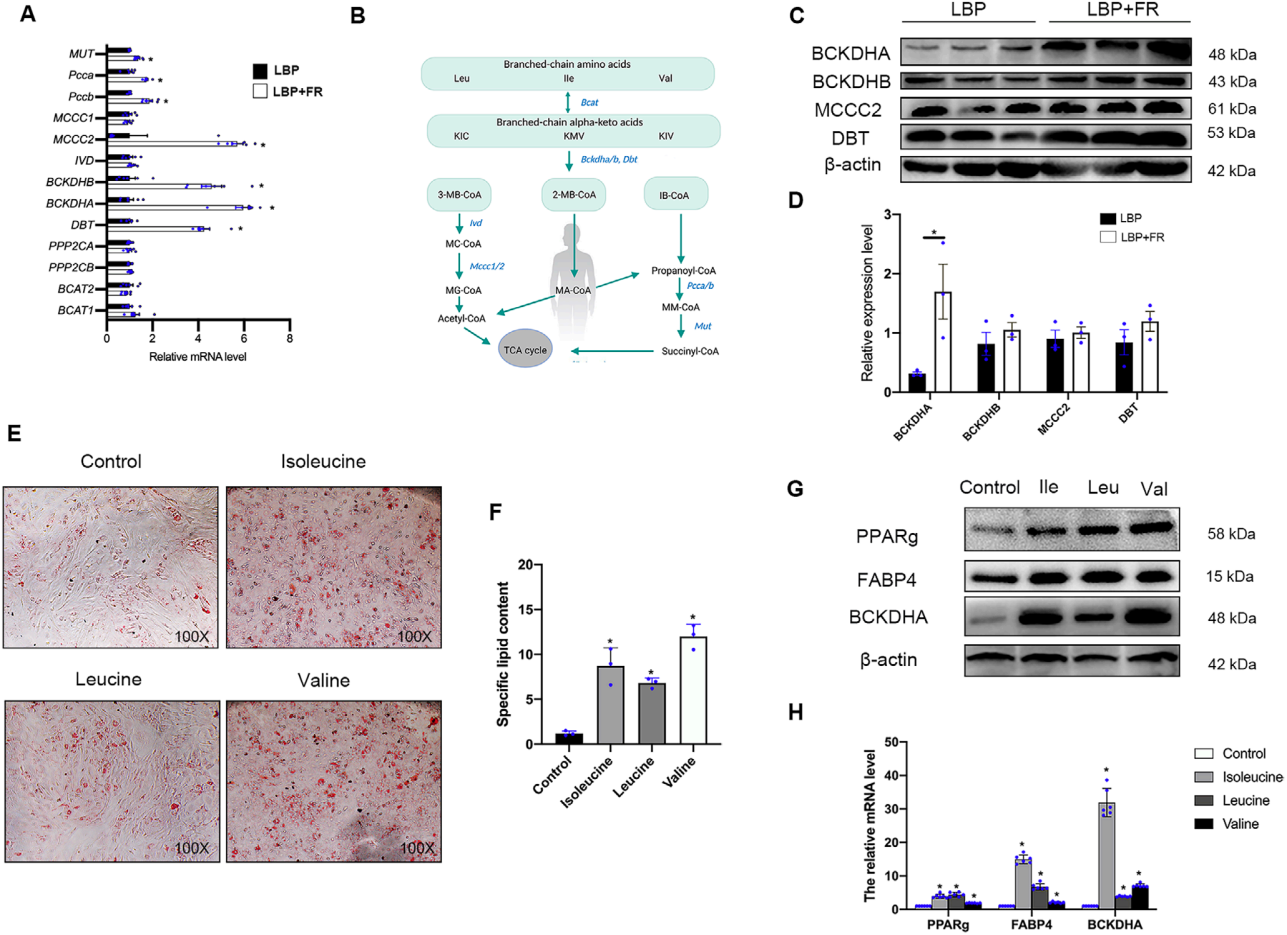
BCAA degradation positively regulates BM‐MSC's adipogenic differentiation. (A) RT‐qPCR analyses of selected BCAA degradation genes in LBP + FR and LBP. (*p < 0.05, LBP + FR vs. LBP, *n* = 6). MUT, Methylmalonyl Coenzyme A Mutase; Pcca, Propionyl Coenzyme A Carboxylase, Alpha Polypeptide; Pccb, Propionyl Coenzyme A Carboxylase, Beta Polypeptide; MCCC1, Methylcrotonoyl‐CoA Carboxylase 1; MCCC2, Methylcrotonoyl‐CoA Carboxylase 2; IVD, Isovaleryl‐CoA Dehydrogenase; BCKDHB, Branched Chain Keto Acid Dehydrogenase E1 Subunit Beta; BCKDHA, Branched Chain Keto Acid Dehydrogenase E1 Subunit Alpha; DBT, Dihydrolipoamide Branched‐chain Transacylase E2; PPP2CA, Protein Phosphatase 2 Catalytic Subunit Alpha; PPP2CB, Protein Phosphatase 2 Catalytic Subunit Beta; BCAT2, Branched Chain Amino Acid Transaminase 2; BCAT1, Branched Chain Amino Acid Transaminase 1. (B) Schematic illustration of the BCAA degradation pathway. Enzymes examined in Figure 6A are shown in blue. Degradation of Leu, Ile, and Val share the same initial steps catalyzed by Bcat2, Bckdha, Bckdhb and Dbt. Leu leucine, Ile isoleucine, Val valine, KIV α‐ketoisovalerate, 3‐MB‐CoA 3‐Methylbutanoyl‐CoA, 2‐MB‐CoA 2‐Methylbutanoyl‐CoA, IB‐CoA Isobutyryl‐CoA, MC‐CoA 2‐Methylcrotonyl‐CoA, MG‐CoA 2‐Methylglutaconyl‐CoA, MA‐CoA 2‐Methylbutanoyl‐CoA, MM‐CoA Methylmalonyl‐CoA. (C, D) Representative immunoblots of BCKDHA, BCKDHB, MCCC2, DBT, and β‐actin in BM‐MSCs from LBP + FR and LBP groups (C) and statistical analyses of densitometric measurements of BCKDHA, BCKDHB, MCCC2 and DBT (D) are shown (*p < 0.05). (E, F) Representative images of Oil Red O staining of lipids (E) and quantification of the number of oil spots (F) in BM‐MSCs from control and BCAA groups cultured in adipogenesis induction medium for 14 days. 100× magnification. (G) Representative immunoblots of PPARg, FABP4, BCKDHA, and β‐actin in BM‐MSCs from the BCAA group. (H) RT‐qPCR analysis results of PPARg, FABP4, and BCKDHA in BCAA derived BM‐MSCs cultured in adipogenesis induction medium for 14 days. (*p < 0.05).

We next detected the regulatory mechanisms of BCAAs on BM‐MSC's adipogenic differentiation. Genes related to BCAA degradation, including BCKDHA, BCKDHB, DBT, MCCC2, Pccb, Pcca, and MUT were upregulated in BM‐MSCs from LBP + FR. (Figure 6A) As shown in Figure 6B, the BCAA degradation pathway's schematic illustration helps us know the roles of different enzymes involved in the BCAA pathway. Then, the changes of these key enzymes (BCKDHA, BCKDHB, DBT, MCCC2) were identified by immunoblot (Figure 6C,D). Among them, the protein levels of BCKDHA complex, an important enzyme that controls the committed and initial steps of BCAA degradation to branched‐chain acylcoA, was significantly upregulated in the LBP + FR group. Taken together, BCKDH's activity was regarded as the most direct factor to affect the BCAA degradation in LBP + FR.

To detect the correlations and functions of BCKDHA and BCAA in BM‐MSCs' differentiation, we tested the effect of the BCAA in an ex vivo cell culture model. We used three BCAAs (valine, isoleucine, leucine) to stimulate the BM‐MSCs separately (Figure S6). BM‐MSCs from BCAA treatment group exhibited increased adipogenesis (Figure 6E,F), accompanied by increased protein levels and mRNA levels of PPARg and FABP4 (Figure 6G,H). Moreover, BCKDHA protein and mRNA level were significantly higher in BCCA group compared with control group. (Figure 6G,H).

Notably, the untargeted metabolomics analysis of serum samples that show two metabolites (Thiamine pyrophosphate (Thpp) and 2‐Methyl‐1‐hydroxypropyl‐ThPP) related to the BCAAs degradation pathway were mainly enriched in the LBP + FR compared with the LBP. (Figure 3 and Table S4) These two metabolites, especially the ThPP, could be required as a coenzyme for the E1 component of the BCKDH complex and can also activate the complex by inhibiting BCKDH kinase (BDK) [24]. Taken together, BCKDHA contributes greatly to the LBP + FR process.

Thus, these observations strongly suggest that amino acid metabolism (mainly intracellular BCAA degradation) and the critical enzyme BCKDHA plays an essential role in BM‐MSC's adipogenesis in LBP + FR.

### 2.8 SIRT4 Boosts Adipogenesis and the Expression of BCKDHA

**FIGURE 7:**
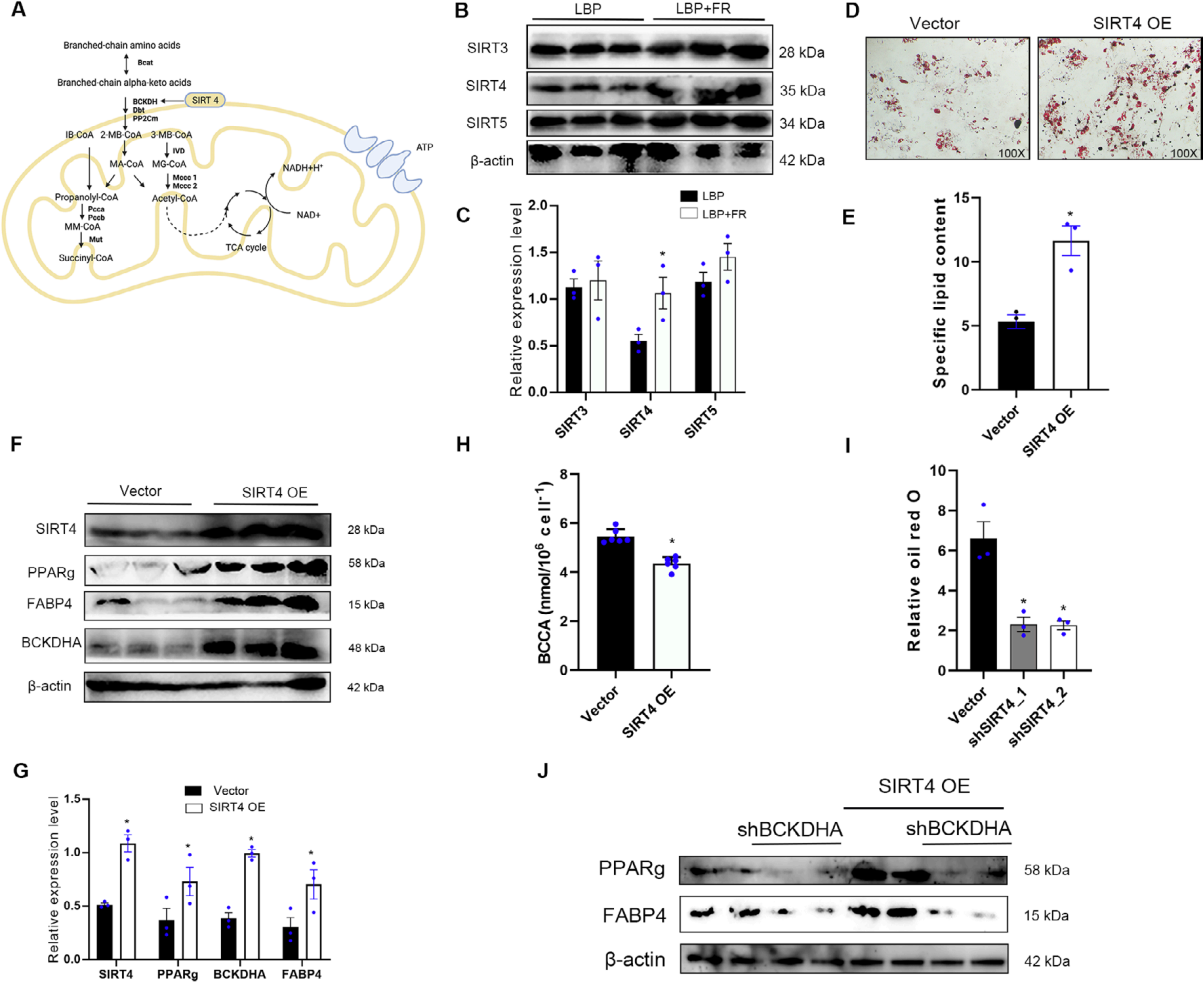
SIRT4 promotes adipogenesis and BCKDHA. (A) Model for SIRT4 regulation of BCAA catabolism. (B, C) Representative immunoblots of SIRT3, SIRT4, SIRT5, and β‐actin in BM‐MSCs from LBP + FR and LBP groups (B) and statistical analyses of densitometric measurements of SIRT3, SIRT4, and SIRT5 (C) are shown (*p < 0.05). (D, E) Representative images of Oil Red O staining of lipids (D) and quantification of the number of oil spots (E) in BM‐MSCs from vector and SIRT4‐overexpressing group cultured in adipogenesis induction medium for 14 days. 100× magnification. (F, G) Representative immunoblots of SIRT4, PPARg, FABP4, BCKDHA, and β‐actin in BM‐MSCs from vector and SIRT4‐overexpressing groups (F) and statistical analyses of densitometric measurements of SIRT4, PPARg, FABP4, and BCKDHA (G) are shown (*p < 0.05). (H) The BCAA content in BM‐MSCs from control and SIRT4‐overexpressing groups after adipo‐differentiation (*p < 0.05). (I) Quantification of relative oil red O in vector, shSIRT4_1 and shSIRT4_2 BM‐MSCs differentiated for 14 days (*p < 0.05). (J) Western blot analysis of PPARg and FABP4 in BM‐MSCs differentiated in14 days overexpressing SIRT4 or control vector and shBCKDHA or control plasmid.

Next, we explored the upstream regulatory mechanisms mediating the BCAA degradation pathway. Sirtuins are NAD^+^‐dependent enzymes conserved from bacteria to human [13] and three sirtuins‐SIRT3, SIRT4, and SIRT5 localize to the mitochondrial matrix, are key regulators of mitochondrial metabolic enzymes [25, 26]. Furthermore, A proteomics research on fibroblasts has found SIRT4 can alter the mitochondrial activity by binding to BCAA catabolic enzymes BCAT [27] and MCCC1 [28, 29]. Given that BCAA degradation is a mitochondrial process, we hypothesized that sirtuins might play a role in increasing BM‐MSC adipogenesis by regulating BCAA degradation (Figure 7A).

After testifying by RT‐PCR and WB, we found a largest increase in SIRT4 expression on protein and mRNA compared with the expression of SIRT3 and SIRT5 in BM‐MSCs of LBP + FR patients (Figures S7A and 7B,C). To interrogate the role of mitochondrial SIRT4 in BM‐MSCs' differentiation, we overexpressed SIRT4 in normal BM‐MSCs. SIRT4 overexpression caused the significant increase in adipogenesis, as assessed by oil red O staining (Figure 7D,E). Besides, SIRT4 overexpression increased expression of genes and proteins related to adipogenesis (PPARg and FABP4) and BCKDHA (Figures S7B and 7F,G).

Given the established relationship between BCAA degradation and SIRT4, we hypothesized that SIRT4 might promote BM‐MSC's adipogenesis by regulating BCAA catabolism. We detected that SIRT4 overexpression decreased the BCAA content in BM‐MSC after 14 days of adipo‐differentiation (Figure 7H). Moreover, we used two different shRNAs to knockdown SIRT4 (shSIRT4) and found that the loss of SIRT4 impaired adipocyte differentiation (Figures 7I and S7C). We also found that SIRT4 knockdown cells had significantly decreased protein and mRNA levels of BCKDHA compared to control BM‐MSCs (Figure S7D,E). To further detect if BCKDHA is necessary for SIRT4‐mediated adipogenesis in BM‐MSCs, knocked down BCKDHA was used in the context of SIRT4 overexpression. We found that BCKDHA knockdown suppresses SIRT4‐mediated increases of PPARg and FABP4 at protein level, suggesting that SIRT4 promotes adipogenesis through BCKDHA (Figure 7J). Overall, these observations strongly suggest that SIRT4 positively regulates BCAA degradation and adipogenesis process in BM‐MSCs.

### 2.9 Gut Microbiome‐ and Metabolome‐Based Prediction of LBP and FR

**FIGURE 8:**
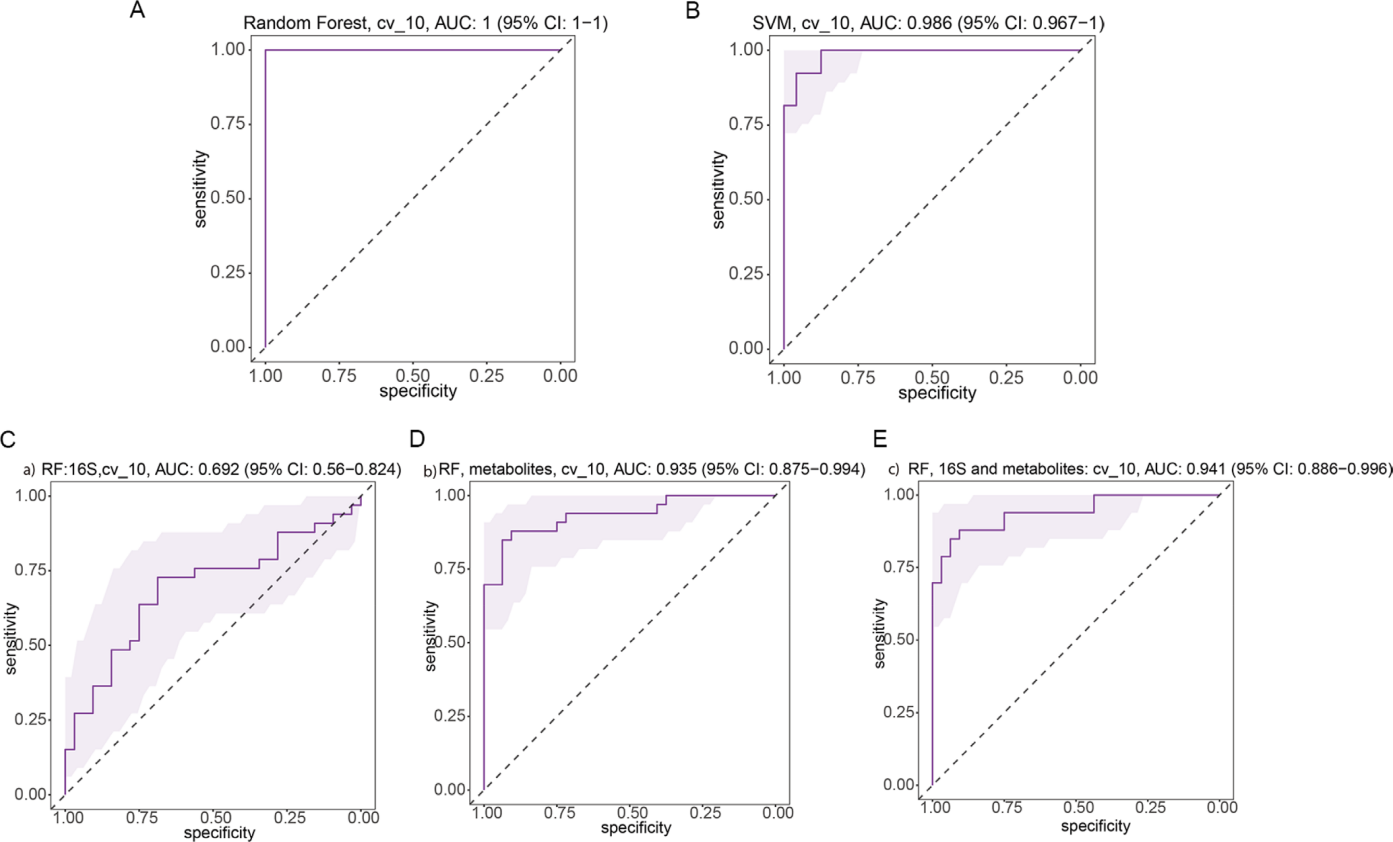
Random forest models to predict LBP and FR. (A, B) Prediction models to predict LBP to healthy control. The microbial signatures of 16S genera have AUC of 1 with 10‐fold cross‐validations for random forest, for support vector machine model, it has an AUC of 0.986. (C–E) Random Forest models to predict LBP types by top 20 16S genus and top 61 metabolites selected by all data constructed model.

To determine whether differences in gut microbiome composition can be regarded as recognition biomarkers for distinguishing LBP patients from healthy participants, we trained Radom Forest (RF) classifiers on relative abundances of 16S tag sequences shared between the two LBP and HC groups. RF model was generated, and the corresponding receiver operating characteristic (ROC) curve was drawn to evaluate its distinguishing ability. As shown in Figure 8A,B, the classifiers yielded nearly perfect predictions (95% area under the ROC curve [AUC]) of LBP. This result shows that the random forest model based on fecal microflora can distinguish LBP patients from healthy individuals, indicating that intestinal microflora information can be used to identify LBP patients. In addition, a second RF classifier was trained to predict LBP with FR by top 20 genus (16S genus) and top 61 metabolites selected by all data constructed models. 10‐fold cross‐validation was applied to reduce the over‐fitting effects. By combining the 16S top genera and top 61 metabolites, the prediction power can achieve as high as an AUC of 0.94, demonstrating that microbial signatures and metabolites can accurately predict the FR in LBP patients. (Figure 8C–E).

We also found that RF model could discriminate LBP patients with FR from LBP subjects with an area under the curve ranging from 0.69 to 0.94 (all 16S genus, AUC = 0.69; all 10 000 metabolites, AUC = 0.935; combination of all 16S genus and metabolites, AUC = 0.94; Figure S8A–C). The 16S has a very low AUC of around 0.6, but the metabolites can predict types of LBP quite accurately with an AUC of 0.88. While combining them together for all the data, the prediction power does not improve too much compared with metabolites only. These results demonstrated that these models based on gut microbiome and serum metabolites could distinguish LBP patients from healthy individuals and differentiated LBP patients with FR from LBP patients without FR in our cohort, suggesting that the gut microbiome and serum metabolite information could be applied to identify patients.

## 3 Discussion

Researchers found chronic LBP with FR share similar basic pathogenetic characteristics with osteoarthritic (OA), including MRI modalities, prevalence, pain, joint degeneration, risk factors, suggested etiologies, and natural history with bone marrow lesions [30]. The VAS difference of 0.9 and the ODI difference of 8% between the LBP + FR and LBP‐FR groups are smaller than the minimal clinically important differences (MCID) reported in the research [31]. Typically, MCID for VAS is a reduction of 1.4–2 points, and for ODI, it is 11%. Although these differences do not reach the MCID thresholds, they still provide useful insights into the variations in pain and disability between the groups. These small differences might still be meaningful in a clinical context. Future studies with larger sample sizes or different methods might help clarify these findings further. We will discuss these limitations and place our results in context with existing research.

There has been an abundance of evidence to support that gut microbiota and its metabolites have been proposed as cofactors in OA [32], but only a few studies have directly looked at the effects of gut dysbiosis on BML [13, 33]. Here, we outlined the landscapes and interaction networks of differential bacterial species and serum metabolites in the LBP with FR gut ecosystem. Disturbance of amino acid metabolism was a hallmark in the gut ecosystem of LBP with FR. Moreover, we utilized an ex vivo cell culture model to demonstrate an LBP + FR‐specific effect of microbiota and metabolites on the BM‐MSC's adipogenesis differentiation, showing important clinical implications. Previous gut microbiome studies focused on the differential bacteria between LBP and non‐LBP [13] and did not clarify how the gut microbiome affects disease development. Our research compared the microbiome composition between LBP + FR, LBP and healthy controls using two methods:16 s rRNA sequencing and metagenomics sequencing. 16 s rRNA sequencing and metagenomic sequencing were prerequisites for screening the critical species associated with LBP with/without FR onset and identifying diagnostic markers for clinical applications.

Despite the exist of geographical variations between three groups (LBP + FR, LBP, and HC), 16 s rRNA sequencing results showed that the bacterial composition of LBP with/without FR was different from that in HC. LBP was characterized by enriched families Streptococcaceae, Prevotellaceae, Rikenellaceae, Alcaligenaceae, Clostridiaceae, and Enterobacteriaceae; reduced families Ruminococcaceae and Lachnospiraceae. At the genus level, LBP with/without FR was distinguished from HC by the expansion of Prevotella, Escherichia, Megamonas, and SMB53, and the reduction in Faecalibacterium, Roseburia, Lachnospira, and Sutterella. Even though we did not find the different microbiome composition at family and genus level in the top abundant species between the LBP + FR group and LBP group, abundance comparisons of other not prominent genera showed that enriched Atopobium, Catenibacterium, and Eubacterium and reduced Aggregatibacter in LBP + FR patients compared to LBP. Only a portion of the gut microbiota community revealed by shotgun sequencing could be detected by 16S rRNA gene sequencing [34]. Shotgun sequencing, in particular, has a greater sensitive ability to identify taxa with less abundant than 16S rRNA gene sequencing when a sufficient number of reads are available. Furthermore, the alterations in metagenomes relate to disease features, the results involved could be utilized as therapeutic targets or the outputs used as fecal biomarkers, although this would need clinical and experimental validation. In this study, we demonstrated that the less abundant genera detected only by shotgun sequencing are biologically meaningful, being able to discriminate between three groups.

Our metagenomic analysis identified seven bacterial species linked with LBP (LBP with/without FR). Metagenomic microbiome sequencing data support an emerging core microbiome signature that LBP was characterized by reduced species of *Bilophila unclassified, E. hallii, A. equolifaciens*, and *L. bacterium 5 1 63FAA* compared to HC. *A. equolifaciens* has been proved to play a significant role in gut microbiota–host interactions, especially LBP (42). *A. equolifaciens* is a well‐known species that produce equol [35]. In menopausal and postmenopausal women, equol has been proven to be critical in reducing bone loss and alleviating muscle and joint pain [36]. Besides, Marloes et al. [13] used 16S rRNA analyses of fecal specimens in a group of 36 overweight or obese people who had or had not back pain. In contrast to our findings, Marloes et al. reported that the genera Adlercreutzia were found in greater abundance in people with back pain than in people who did not have back pain (*p* = 0.0008). These disparate findings are plausibly due to significant differences in the study cohorts. In Marloes's study, all participants were overweight or obese people who additionally had a higher BMI (≥ 25 kg/m^2^), compared to our study cohort with a common BMI (≤ 24 kg/m^2^). In addition to this, other characteristics, including demographic, clinical, biochemical characteristics, diet and physical activity are different between Marloes's study and our research.

Bilophila belongs to the Desulfovibrionaceae family, which produces hydrogen sulphide (H_2_S). Metabolizes sulphated substances to create H_2_S, which can cause inflammation, cause epithelial cell genotoxicity and cytotoxicity, and disrupt intestinal barrier function [37]. Importantly, H_2_S has been reported to exert antinociceptive effects [38] and sulfidogenic bacteria have recently been linked to the etiology of chronic metabolic diseases [39]. *E. hallii* and *L. bacterium 5 1 63FAA*, with its ability to create large levels of SCFAs such as butyrate and propionate, has been hypothesized to be vital in maintaining gut microbial metabolic health and homoeostasis [40, 41]. Even though, there is no clear evidence about the effects of SCFAs on LBP, SCFAs have been demonstrated to have an direct effect on inhibition of bone resorption or osteoclast formation either via activation of G Protein‐Coupled Receptors (GPCR) or through histone deacetylase (HDAC) inhibition [42]. Obviously, sufficiently powered studies in large cohorts are still needed to determine the specific alterations of the microbiome with LBP.

In this paper, we focused on the differentiated species between LBP + FR and LBP. Three species, including identified enriched *R. gnavus* , depleted *R. hominis* , and *L. bacterium 8 1 57FAA* to distinguish LBP + FR from LBP and HC group. *R. gnavus* is the only enriched species in LBP + FR compared to LBP and HC group. In most studies about *R. gnavus* , increased relative abundance of *R. gnavus* was mainly linked with Crohn's disease (CD), a major form of inflammatory bowel disease [43]. It is worth noting that musculoskeletal pain occurs in 9%–53% of IBD patients and is considered the most common extraintestinal manifestations [44]. Although it is hard to connect the musculoskeletal pain and IBD though the current knowledge about *R. gnavus* , these research findings provide the key to understanding the potential function of *R. gnavus* in musculoskeletal diseases. Despite numerous studies associating *R. gnavus* with various inflammatory diseases [45, 46], no molecular mechanisms to explain these correlations have been developed, and here we identified a possible mechanism for LBP + FR and *R. gnavus* . To further prove that these key species in the gut microbiome played a driving role to FR requires more in vivo experiment such as fecal microbiota transplant (FMT).

Gut bacteria are now well recognized as having a considerable influence on various metabolic pathways in the host. Both statistical correlation (co‐expression network) and metabolic function support this conclusion in our study. In co‐occurrence analysis among the gut microbiome and metabolites, changed microbiome species, *R. gnavus* and *R. hominis* , were substantially correlated with serum metabolites involved in most of pathways (Figure 4). Co‐occurrence analysis shown the connection between BCAA degradation and *R. gnavus* /*L. bacterium 8 1 57FAA*. Gut bacteria can affect serum BCAA levels in several ways. They can alter how BCAAs are absorbed from the gut, produce substances that change BCAA metabolism in the liver, or affect the gut barrier's function. These bacteria can also impact inflammation levels, which in turn can influence BCAA concentrations in the blood. The specific mechanisms by which the bacterial species influence serum BCAA metabolism remain unknown and require further experimental validation. Besides, in metabolic analysis, BCAAs degradation pathway was the most enriched pathway in LBP + FR group. This finding is consistent with the functionality analysis of fecal microbiome based on metagenomics sequencing (Figure 2I), KEGG pathway analysis showed BCAA pathway with functions related to metabolic pathways and signal transduction in LBP + FR group. Functional assignments and network analysis revealed that BCAA degradation pathway serves as the metabolic mediator for the cross talk between host and the microbiome and thus BCAA related pathways would differ in the disease condition compared to the healthy state. BCAA have key physiological roles in regulating protein synthesis, metabolism, food intake, and aging [47]. BCAA also showed a strong relationship with metabolic diseases, including metabolic syndrome, type 2 diabetes, and urea cycle disorders [47, 48]. And LBP with FR could be characterized as a disorder of lipid metabolism in bone marrow. Even though we have demonstrated the relationship between BCAA and gut microbiome through co‐occurrence analysis of the metagenomic sequencing and untargeted metabolome, more direct evidence is needed to establish their correlation.

Interestingly, Marloes et al. also found overweight or obese individuals with back pain had a higher abundance of the genera Roseburia than those without back pain [13]. On the one hand, this result confirms the prominent role of Roseburia in LBP. On the other hand, combined with the results of our study, the enrichment of Roseburia was changeable in different LBP conditions, like LBP + obesity or LBP + FR, which means Roseburia abundance could affect various metabolic pathways in LBP. Besides, Roseburia has been detected to associate with several diseases, including irritable bowel syndrome, ulcerative colitis, obesity, type 2 diabetes, nervous system disorders, and allergies [49, 50]. However, it must be acknowledged that these clinical findings are preliminary, and the impact of environmental and demographic factors cannot be totally ruled out.

Co‐occurrence analysis found the different microbial species and serum metabolites in LBP group were consistently mapped into Porphyrin and chlorophyll metabolism. Porphyrins are a class of metabolites that play a role in the manufacture of life‐sustaining chemicals [51]. Their tetrapyrrole ring‐like shape allows them to bind metal ions like iron and magnesium, making them useful as intermediates in the heme and chlorophyll production pathways, respectively [52]. The levels of bacterial porphyrin on the skin were shown to be substantially greater in acne vulgaris (acne) patients [53]. The dominant porphyrin species produced by *P. acnes* , coproporphyrin III, was shown to induce *Staphylococcus aureus* aggregation and biofilm formation in the nostrils [54], which means *P. acnes* could be a preliminary cause of severe sepsis caused by *S. aureus* . All these information points to the connection between *P. acnes* and Porphyrin and chlorophyll metabolism. More interesting, researchers have found the evidences that supported the close relationship between the LBP (especially LBP + FR group) and *P. acnes* [55]. In fact, there have always been a lot of doubts that the exist of *P. acnes* in disc samples from LBP patients belongs to samples contamination [56]. Our results provide possible clues and future directions about serum metabolites clues in the relationship between LBP and *P. acnes* . We hypothesize that *P. acnes* , as a skin commensal, could enter the circulatory system through non‐invasive procedures such as brushing teeth, and arrive at disc eventually. *P. acnes* could be a contributing factor to LBP through regulating Porphyrin and chlorophyll metabolism in serum. The role of *P. acnes* shows synergism with the gut differential microbial species we mentioned in Figure 2. However, the functions and metabolic activities of skin microbial communities and their interactions with the host are still not well understood.

The MRI signal intensity of the bone marrow in LBP with FR reflects a lipid conversion. BM‐MSCs belong to the type of stem cells, and it has the ability to differentiate to the adipocytes or osteoblasts in bone marrow [57]. BM‐MSCs tend to differentiate into adipocytes rather than osteoblasts with aging, which leads to progressive accumulation of fat and bone loss [58]. We demonstrated that BM‐MSCs from patients with LBP + FR exhibit a strong adipogenesis capability. Connected the characteristics of MRI image and RNA sequencing results, BM‐MSCs' differentiated function play a critical role in FR in bone marrow from LBP + FR patients. Moreover, we found the expression of fatty acids related cell receptor GPR 41/43 on BM‐MSCs from LBP + FR patients was higher than the BM‐MSCs from LBP patients. BM‐MSCs from LBP + FR patients were easier to influence by some fatty acid produced by gut microbiome. Noteably, differentiated microbiome in LBP + FR, *R. gnavus* belongs to the family Ruminococcaceae, and *R. hominis, L. bacterium 8 1 57FAA*, belong to family Lachnospiraceae, are proved to be the main butyrate producing‐bacteria in the human gut [59]. These results shown gut microbiome in LBP + FR patients has the potential to regulate BM‐MSCs adipogenesis through SCFAs. We did not find evidence of SCFAs from untargeted blood metabolism. To further detect the relationship between SCFAs and FR in LBP, targeted metabolomics about SCFAs should be done in the future.

Even though BM‐MSCs' differentiate in response to many factors, little is known about how metabolism drives differentiation. Here, we demonstrated that certain amino acids, particularly BCAAs, are consumed during BM‐MSCs' differentiation. BCAA catabolism supports mitochondrial respiration at this stage of BM‐MSCs' adipogenesis.

The process of BM‐MSCs differentiated into adipocytes in bone marrow was ATP‐dependent. BCAA‐induced mitochondrial respiration in adipogenesis may facilitate ATP generation for lipogenesis. These observations point to the importance of mitochondria in BM‐MSCs' differentiation process. Our study highlights the fundamental role of mitochondria as the site of providing enough energy to BM‐MSCs' adipogenesis.

Importantly, SIRT4 and induction of BCAA catabolism amplify expression of PPARg and FABP4 and its targets, which also include BCAA catabolism enzymes: BCKDHA.

Although, it is unclear how SIRT4 function in the mitochondria impacts transcriptional activity of PPARg and FABP4. The work from Laurent et al. showed that the loss of SIRT4 increases expression of PPARa target genes in fatty acid catabolism [60]. Mechanistically, in the absence of SIRT4, NAD+ levels are elevated and activate SIRT1 that can then be recruited to PPARa and co‐regulate transcription. Previous work identified the link between SIRT4 and BCAA catabolism through MCCC1 in the liver [61]. Here, we find that SIRT4 also enhances BCKDHA activity in BM‐MSCS and that loss of this regulation results in decreased adipogenesis. SIRT4 has been reported to bind to numerous substrates involved in BCAA catabolism, including MCCC1, BCKDHA, DBT, and dihydrolipoyl dehydrogenase (DLD) [29, 61], raising the possibility that SIRT4 engages this pathway at multiple enzymatic nodes. Additional investigation is required to determine which SIRT4‐BCAA catabolism enzyme interactions are most important for promoting adipogenesis in BM‐MSCs from LBP + FR patients. Given that numerous enzymatic activities of SIRT4 have been revealed [62], it is possible that SIRT4 augments BCKDHA activity in BM‐MSCs through a previously unidentified activity.

In summary, using multi omics data, we have presented evidence that LBP or LBP + FR were characterized by gut bacterial disturbances and serum metabolites, which represented the overall disturbances of LBP or LBP + FR gut ecology. Furthermore, disturbance of microbial amino acid metabolism (BCAA degradation pathway) was a potential source of disease biomarkers in LBP + FR. Moreover, this work identifies a significant process in adipogenesis regulated by SIRT4‐mediated stimulation of BCAA metabolism with important developmental implications in BM‐MSCs. Together, these findings provide new directions to uncover pathogenesis and develop novel diagnostic strategies for chronic LBP with FR.

## 4 Material and Methods

### 4.1 Patients' Samples

107 chronic LBP Patients with/without FR and 31 healthy controls were recruited. The ethics approval was obtained from the Human Research and Ethics Committee of the University of New South Wales (UNSW), Sydney, Australia (HC190680) and First Affiliated Hospital, Sun Yat‐sen University, China (No: 2021–270). Due to the COVID‐19 pandemic, sample collection for this collaborative project was exclusively conducted in China. For the chronic LBP group, participants were recruited for this project if they were any gender aged between 20 and 70 years, willing to undergo an MRI of lumbar spine for diagnosing FR, had chronic LBP at least half the day in the past 6 months, and no previous low back surgery, and had not been diagnosed with ankylosing spondylitis, osteoporotic vertebral fracture, multiple myeloma, metastatic carcinoma and psoas abscess. Healthy individuals were adults with no history of LBP and FR or spinal disease. Moreover, all participants with known gastrointestinal disorders, including Irritable Bowel Syndrome (IBS), Inflammatory Bowel Disease (IBD), or other gut or metabolic diseases, were excluded from the study to prevent confounding effects related to these conditions. Advertisement for participant recruitment (participants with LBP or NO LBP) was placed at the First Affiliated Hospital. Participants were excluded from the project if they had any antibiotics or oral corticosteroids in past 1 month. These drugs will interfere with the gut microbiome composition.

### 4.2 Specimen Collection and Library Preparation

All participants will be given a fecal collection kit and guided to generate their faces within 2 days. All fecal samples were frozen and stored at −80°C in 1 day after their receipt. Microbial DNA was extracted from stool by MOBIO PowerSoil DNA Isolation kit (MOBIO Laboratories, Carlsbad, CA, USA) with bases‐beating step following the manufacture's protocol. Sequencing libraries were generated using NEB Next Ultra DNA Library Prep Kit for Illumina (New England Biolabs, MA, USA) following manufacturer's recommendations and index codes were added. The library was sequenced on an Illumina NovaSeq 6000 platform and 150 bp paired end reads were generated.

### 4.3 16S rRNA Gene Sequencing Analysis

We merged, applied quality control and clustered the 16S rRNA gene reads into operational taxonomic units (OTUs) by using QIIME V.1.9.1. Taxonomic groups were based on the Greengenes Database V.13_8 using closed reference to perform referenced‐based OTU clustering [63, 64]. Values for alpha diversity (Chao1 Index, Shannon's Index, Phylogenetic Diversity (PD) Whole Tree Index and observed OTUs), beta diversity (Unweighted UniFrac distance metrics), and principal coordinate analysis (PCoA) employed based on the Bray_Curtis distance, Unweighted_Unifrac and Weighted_Unifrac distance were generated by QIIME V.1.9.1. Permutational multivariate analysis of variance was performed to determine if the compositions of microbiota differed between groups. Linear discriminant analysis effect size (LEfSe) was performed to determine the features most likely to explain the differences between groups.

### 4.4 Metagenomic Sequencing Analysis

The raw data processing using Trimmomatic [65] (v.0.36) was conducted to acquire the Clean Data for subsequent analysis. MEGAHIT (Version v1.0.6) were used to assemble the metagenomics Clean Data. Scaftigs were obtained by mixing assembled scaffolds from N connection. The contigs with a length of or more than 500 bp were selected as the final assembling result. Open reading frames were predicted from both single and mixed Scaftigs using MetaGeneMark (Version 3.38) and filtered the length information shorter than 90 nt from the predicted result with default parameters. Then CD HIT (Version:4.7) is adopted to remove redundancy and obtain the unique initial gene catalog. All predicted genes with a 95% sequence identity and 90% coverage were clustered. Reads and number of reads in each sample were obtained by using BBMAP software.

We annotated gene sets using DIAMOND software based on the NCBI NR database. Each gene is assigned to the highest‐scoring taxonomy based on a unified database. This achieves simultaneous assessment of the gut microbiome of LBP patients with or without FR. The DIAMOND software is adopted to blast Unigenes to KEGG annotation. Alpha‐diversity analysis, including Chao‐1‐richness index, Observed‐otus, Shannon's diversity index, and PD‐whole‐tree index was utilized conducted and visualized using the fossil and vegan packages in R. PCoA was used to visually evaluate the overall difference and similarity of gut bacterial communities between the LBP patients with or without FR and HC groups.

Group differences were tested by using the PERMANOVA. The differential bacterial species between the three groups were identified using LEfSe with linear discriminant analysis (LDA) score > 2. Furthermore, Wilcoxon rank‐sum test was used to identify the differential species and metabolites between different groups (false discovery rate, < 0.05). Then, the correlated microbial genes in the KEGG pathway were detected.

### 4.5 Serum and BM‐MSCs Preparation

Serum samples were collected from the whole blood by serum‐separator tubes, and serum samples should be stored at −80°C within 2 h. Bone marrow (BM) samples were obtained from LBP patients, both with and without FR. Under local anesthesia, approximately 15–20 mL of bone marrow was aspirated from the posterior iliac crest of each donor. The samples were collected in a 50 mL tube containing an equal volume of heparinized (10 U/mL) phosphate‐buffered saline (PBS) to prevent clotting. Six participants were included in each group, and the groups were matched for age and BMI to eliminate potential confounding factors related to adipogenic tendencies. BM‐MSCs samples were separated from the bone marrow and stored at −80°C immediately. Sample treatments were following the recommended processing guides for untargeted metabolomic study.

### 4.6 Metabolomics Measured by LC‐MC

Untargeted metabolomics Profiles were performed by Beijing Genomics institution (BGI). Advanced mass spectrometer Xevo G2‐XS QTOF (Waters, UK) was used for comparing the serum metabolites signatures of three groups. Commercial software Progenesis QI (version 2.2) (Waters, UK) and the BGI's own metabolomics software package metaX were used for mass spectrometry data analysis, wherein identification was based on KEGG database. PCoA was applied to discriminate the samples from different groups visually. The cluster analysis was performed to group the selected differential metabolites. Metabolic pathway in which the differential metabolites involved were enriched referred to the KEGG pathway and database.

### 4.7 Metabolomic and Metagenomic Data Integration

Correlation analysis between metagenomic and metabolomic data were undertaken using genomes and metabolites identified as significantly different between LBP + FR, LBP, and healthy samples incorporating adjustment for age, sex, and BMI. The co‐occurrence among the gut microbiome and serum metabolites was calculated on the basis of the relative abundances by Spearman's rank correlation coefficient (*p* < 0.05).

The network layout was calculated and visualized using a circular layout by the Cytoscape software. Only edges with correlations greater than 0.5 were shown in the bacteria and serum metabolites, and unconnected nodes were omitted. Correlation coefficients with a magnitude of 0.5 or above were selected for visualization in Cytoscape.

### 4.8 Branched Chain Amino Acid Assay

Following the manufacturer's instructions, a commercial colorimetric measurement kit (ab83374, Abcam, United Kingdom) was utilized to quantify the BCAA levels in serum and cell culture media samples. 2.5 times diluted serum samples and BCAA standards were incubated with substrate mix and an equal amount of enzyme at room temperature for 30 min. The absorption at 450 nm was measured with a microplate reader, and the BCAA's level was calculated by using the standard curve.

### 4.9 BM‐MSCs‐Isolation and Ex Vivo Culture

The mononuclear cells (MNCs) were extracted from the marrow cavity of intervertebral body according to a widely used protocol [66]. Briefly, the collected bone marrow was mixed with PBS (1:2) gently. The mixture was equally layered onto equal volume of Ficoll (1077 g/m, GE health care, Chicago, USA) and collected buffy coat by using a density gradient centrifugation. Then the isolated buffy coat was washed twice with PBS and spined with centrifugation. The gotten red blood cell were seeded in DMEM low glucose with 10% FBS (Gibco, Life Technologies Ltd. Paisley, UK) at a density of 160 000/cm [2]. All non‐adherent cells were removed. The culture medium was changed twice a week and the BM‐MSCs could be harvested by the trypsin (Thermofisher, USA) after about 3~5 days and transferred to a new flask.

### 4.10 Immune‐Phenotype

BM‐MSCs were counted and divided 1 × 10^5^/tube and then re‐suspended in 100 μL of antibodies mix. Subsequently, cells were incubated for 30 min at 4°, washed and analyzed with a FACSCanto flow cytometer (BD PharMingen) and with the FACSDiva software (Tree Star Inc. Ashland, OR). BM‐MSCs were characterized with some special monoclonal antibodies again CD105, CD29, CD34, and CD45 (Cyagen, #HUXMX‐09011) associated with different fluorochromes.

### 4.11 Oil Red O Staining and Quantification

BM‐MSCs were culture in the adipogenesis induction medium (α‐MEM containing 0.5 mM 3‐isobutyl‐1‐methylxanthine, 1 μM dexamethasone, 5 μg/mL insulin, and 10% FCS) for 14 days. The medium was replaced twice a week. Oil Red O was performed to detect the mature adipocytes. In briefly, BM‐MSCs were washed twice in PBS, fixed with 3.7% formalin, washed with 60% isopropanol, and then stained with 0.3% Oil Red O solution. Excess stain was removed by washing the cells with sterile water, and cells were then dried for imaging. For quantification, lipid droplets were solubilized in 100% isopropanol and quantified by determining the resultant absorbance at 492 nm. Cells were counterstained with 0.01% crystal violet and extracted with 100% methanol and absorbance was measured at 570 nm.

### 4.12 Gene Expression Analysis by RNA Sequencing

Total RNA extraction was done from BM‐MSCs after different treatments with TRIzol reagent. Subsequently, the RNA samples were sent to BGI Co. LTD (Shenzhen, China), and BGISEQ‐500 platform was applied to perform RNA‐sequencing. Differentially expressed genes were determined based on Q value (Adjusted *p*‐value) < = 0.05.

### 4.13 qRT‐PCR Analysis

Total RNA from BM‐MSCs was extracted by the TRIzol reagent (Invitrogen). The RNA concentration was measured by the Nanodrop 2100 and reverse transcription was performed using 1 μg total RNA. Total RNA was reverse‐transcribed into the first‐strand cDNA using the Superscript First‐Strand Synthesis Kit (Invitrogen). cDNA transcripts were quantified by Rotor Gene Real‐Time PCR System (Qiagen) using SYBR Green (Biorad). Primer sequences were shown in Table 2.

**TABLE 2:** The primer sequences used for quantitative real‐time PCR.

Primer	Forward primer (5′ → 3′)	Reverse prime (3′ → 5′)
h‐PPARg (NM_001172698)	GATGCCAGCGACTTTGACTC	ACCCACGTCATCTTCAGGGA
h‐FABP4 (NM_001442)	ACTGGGCCAGGAATTTGACG	CTCGTGGAAGTGACGCCTT
h‐IGFBP2 (NM_000597)	GACAATGGCGATGACCACTCA	CAGCTCCTTCATACCCGACTT
h‐MGST3 (NM_004528)	GGCCCACCTAGCCATCAATG	CGCTGAATGCAGTTGAAGATGT
h‐LPL (NM_000237)	TCATTCCCGGAGTAGCAGAGT	GGCCACAAGTTTTGGCACC
h‐FASN	AAGGACCTGTCTAGGTTTGATGC	TGGCTTCATAGGTGACTTCCA
(NM_004104)		
h‐IGF2 (NM_001007139)	GTGGCATCGTTGAGGAGTG	CACGTCCCTCTCGGACTTG
h‐GPX3	AGAGCCGGGGACAAGAGAA	ATTTGCCAGCATACTGCTTGA
(NM_002084)		
h‐CD36 (NM_000072)	GGCTGTGACCGGAACTGTG	AGGTCTCCAACTGGCATTAGAA
h‐ACACA (NM_198838)	ATGTCTGGCTTGCACCTAGTA	CCCCAAAGCGAGTAACAAATTCT
h‐BCAT1 (NM_005504)	GTGGAGTGGTCCTCAGAGTTT	AGCCAGGGTGCAATGACAG
h‐BCAT2 (NM_001190)	GCTCAACATGGACCGGATG	CCGCACATAGAGGCTGGTG
h‐PP2CB (NM_001009552)	CTGAACGAGAACCAAGTGCG	ACGAACCTCTTGCACATTTGA
h‐PP2CA (NM_002715)	CAAAAGAATCCAACGTGCAAGAG	CGTTCACGGTAACGAACCTT
h‐BCKDHA (NM_001164783)	CTACAAGAGCATGACACTGCTT	CCCTCCTCACCATAGTTGGTC
h‐BCKDHB (NM_000056)	TGGAGTCTTTAGATGCACTGTTG	CGCAATTCCGATTCCAAATCCAA
h‐DBT (NM_001918)	CAGTTCGCCGTCTGGCAAT	CCTGTGAATACCGGAGGTTTTG
h‐IVD (NM_002225)	ATGGCAGAGATGGCGACTG	TAGCCCATTGATTGCATCGTC
h‐MCCC2 (NM_022132)	AAAGCCCGAGCACTTCACATA	TCCAATGCCTGTAATAATGCCAC
h‐MCCC1 (NM_020166)	GCTGCACAGGCTATCCATCC	CACCATGATAACCCTCCACAAC
h‐Pccb (NM_000532)	AACGAACGCATCGAAAACAAG	CCTGGCTGTTAGCTTTCCTCG
h‐Pcca (NM_001127692)	GCAAGAAGATGGGCATTAAGACA	GCCAACACAGACAGCCTCAT
h‐MUT (NM_000255)	AGAAGACCTAATATGGCACACCC	ATGGTCCAGGGCCTAAAGGTA
h‐SIRT3 (NM_001017524)	ACCCAGTGGCATTCCAGAC	GGCTTGGGGTTGTGAAAGAAG
h‐SIRT4 (NM_012240)	GCTTTGCGTTGACTTTCAGGT	CCAATGGAGGCTTTCGAGCA
h‐SIRT5 (NM_001193267)	GCCATAGCCGAGTGTGAGAC	CAACTCCACAAGAGGTACATCG
h‐GAPDH (NM_001256799.3)	CATGTTCGTCATGGGTGTGAA	GGCATGGACTGTGGTCATGAG

Abbreviation: h‐, human.

BM‐MSCs were washed with phosphate‐buffered saline (PBS) three times, fixed with 4% paraformaldehyde for 15 min, permeabilized in 0.3% Triton X‐100 for 10 min and blocked with 10% normal goat serum for 1 h at room temperature. The following antibodies were used as primary antibodies: FFAR 3 (1: 100, #PA5‐97745, Invitrogen) and GPR 43(1: 200, #BS‐13536R, Thermo Scientific). Alexa Fluor 488 Dye‐conjugated secondary antibody (Invitrogen) was used for detecting indirect fluorescence. Then mounted on glass slides with Vectashield (Vector laboratories).

### 4.14 SDS Page Immunoblot

BM‐MSC lysates were prepared by RIPA lysis buffer (catalog #P0013B; Beyotime Biotechnology, Shanghai, China) supplemented with PMSF protease inhibitor on ice for 30 min. Bicinchoninic acid (BCA) protein assay kit (catalog #P0010S; Beyotime Biotechnology) was used to quantify the protein concentration according to the instruction. Cell proteins were isolated by the SDS‐PAGE blotted on PVDF membranes (Millipore). And then blocked with 5% fat‐free dry ilk at RT for 1 h. The PVDF membranes were incubated with specific antibodies to related genes at 4°C. Next incubated with appropriate HRP‐conjugated secondary antibodies and exposed to x‐ray films.

### 4.15 Statistical Analysis

The numbers of independent experiments can be found in the relevant figure legends. One‐way analysis of variance (ANOVA) was used to compare continuous variables, which were displayed as means ± SD. Data of metagenome and metabolome were presented as means ± standard error of the mean (SEM). GraphPad Prism and excel were used for statistical analysis. The statistical significance level was set at *p* value of < 0.05.

## Author Contributions

W.L. conceptualized the project and planned, executed, and prepared the work for publication. A.D.D. and A.D. initiated the study and along with Z.Z. provided supports, supervision. W.L., J.T., J.Z., Q.Y., W.D., S.Y., C.Y., and J.Z. collected samples, J.Z's. lab and X.J provided analytical support. W.L. and J.T. performed all experiments and produced all figures and tables. X.B. and K.L. helped to analyze the data.

## Conflicts of Interest

The authors declare no conflicts of interest.

## Supporting information


**Figure S1.** Illustration of chronic low back pain with fatty replacement.
**Figure S2.** Alpha diversity indices and Beta diversity of LBP + MCs, LBP‐MCs and HC control stool samples.
**Figure S3.** Differences in bacterial composition between HC, LBP + MCs and LBP‐MCs cohorts.
**Figure S4.** Metagenomic analysis for LBP, LBP + FR and HC groups.
**Figure S5.** Differences in bacterial composition between HC, LBP + MCs and LBP‐MCs cohorts.
**Figure S6.** Flow identification of BM‐MSCs.
**Figure S7.** Increase in BCAA (valine, isoleucine, leucine) dose impacts on BM‐MSCs viability in ex vivo model.
**Figure S8.** SIRT4 boosts adipogenesis on BM‐MSCs.
**Figure S9.** Random Forest models to predict LBP different types.

**Table S1.** A total of 343 discriminative bacterial species between HC, LBP + FR and LBP cohorts.
**Table S2.** Comparison of relative taxonomic abundance at family and genus level in HC, LBP + FR and LBP cohorts.
**Table S3.** Comparison of underlying disease‐correlated KEGG Orthologies (KOs) between LBP + FR, LBP and HC groups.
**Table S4.** The top 50 differential fecal metabolites and enriched pathways in serum samples from the LBP + FR group.
**Table S5.** RNA sequencing Detailed results of GO and KEGG enrichment analysis.
**Table S6.** Characteristics of the study population in Fecal Metagenomic Sequencing Analysis.
**Table S7.** Characteristics of the study population in Fecal Metabolome Analysis.

## Data Availability

The 16S rRNA amplicon and metagenomic sequencing data have been deposited in National Center for Biotechnology Information (NCBI) with the primary accession code: PRJNA822996. The mass spectrometry raw data have been deposited on the MetaboLights (ID: MTBLS4644). The remaining data are available within the article, Supporting Information or available from the authors upon request.
