# Correction to “Gut Microbiome and Metabolome Changes in Chronic Low Back Pain Patients With Vertebral Bone Marrow Lesions”

**DOI:** 10.1002/jsp2.70074

**Published:** 2025-05-15

W. Li, J. Tu, J. Zheng, et al., Gut Microbiome and Metabolome Changes in Chronic Low Back Pain Patients With Vertebral Bone Marrow Lesions. *JOR Spine* 8 (2025): e70042, https://doi.org/10.1002/jsp2.70042

In the article cited above, Affiliations 8 and 9 were mistakenly merged. This has now been corrected, and 16 author affiliations are now shown.

For Figure 6E, the image scale is mistakenly given as 100×. The image below shows the correct scale, which is 40×.

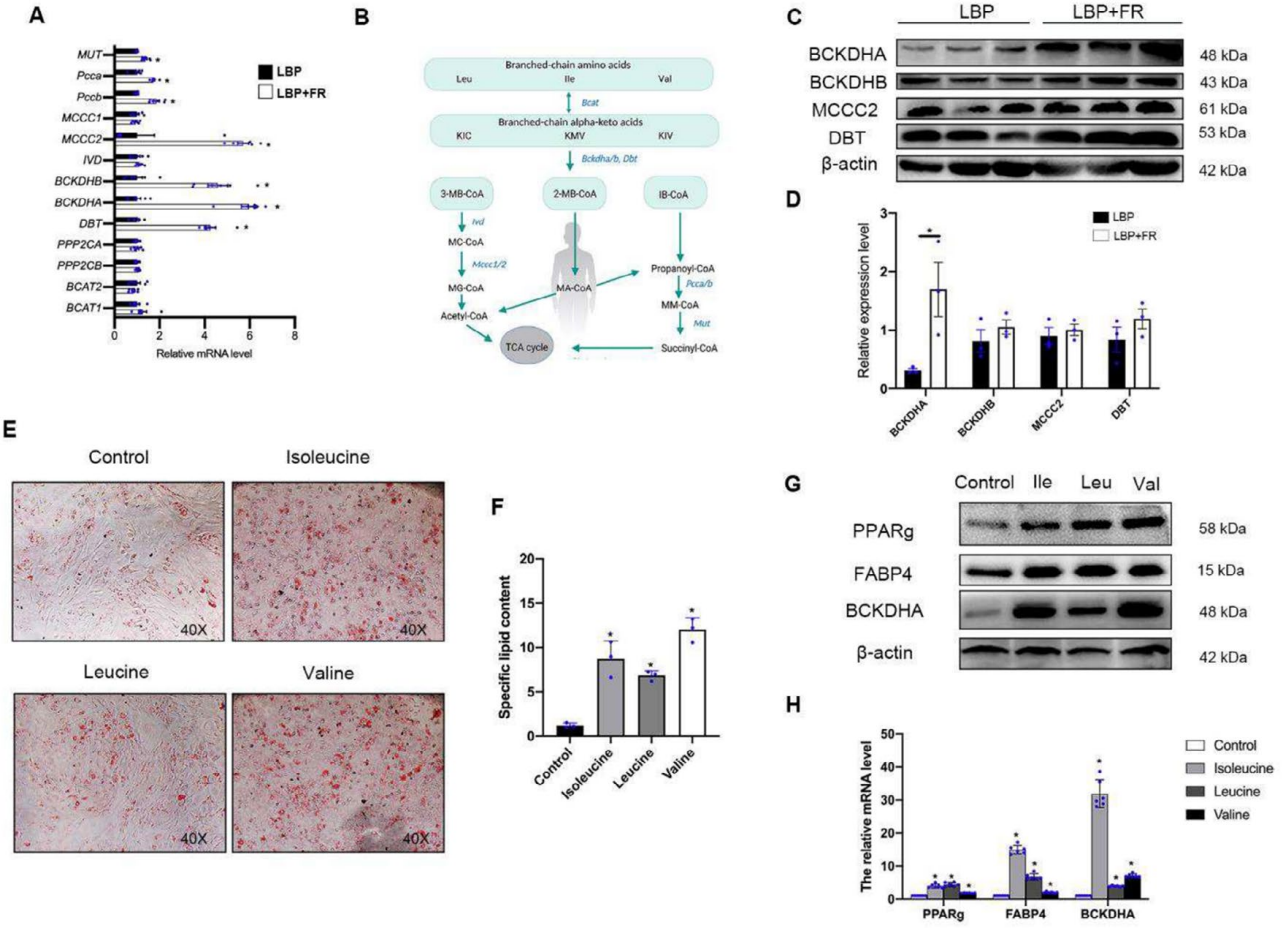

We apologize for this error.

