# Supplementary material for: Gut Microbiome and Metabolome Changes in Chronic Low Back Pain Patients With Vertebral Bone Marrow Lesions
Source: JOR Spine. 2025 Jan 27;8(1):e70042. doi: 10.1002/jsp2.70042 (PMC11772216; doi:10.1002/jsp2.70042)
Supplement: Supplementary file 1 — Figure S1. Illustration of chronic low back pain with fatty replacement. Figure S2. Alpha diversity indices and Beta diversity of LBP + MCs, LBP‐MCs and HC control stool samples. Figure S3. Differences in bacterial composition between HC, LBP + MCs and LBP‐MCs cohorts. Figure S4. Metagenomic analysis for LBP, LBP + FR and HC groups. Figure S5. Differences in bacterial composition between HC, LBP + MCs and LBP‐MCs cohorts. Figure S6. Flow identification of BM‐MSCs. Figure S7. Increase in BCAA (valine, isoleucine, leucine) dose impacts on BM‐MSCs viability in ex vivo model. Figure S8. SIRT4 boosts adipogenesis on BM‐MSCs. Figure S9. Random Forest models to predict LBP different types. [file JSP2-8-e70042-s001.docx]

**Supplementary figures**

**
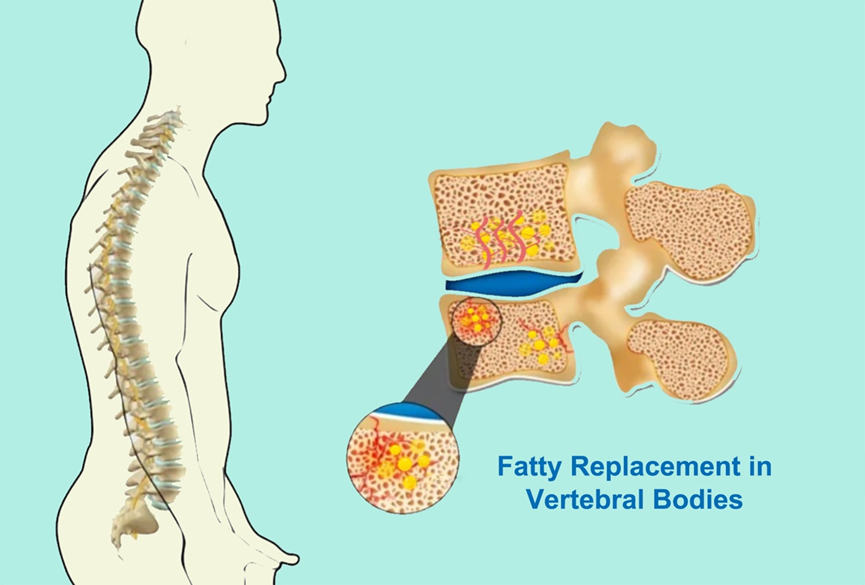
**

**Supplementary figure 1: Illustration of chronic low back pain with fatty replacement.**

**
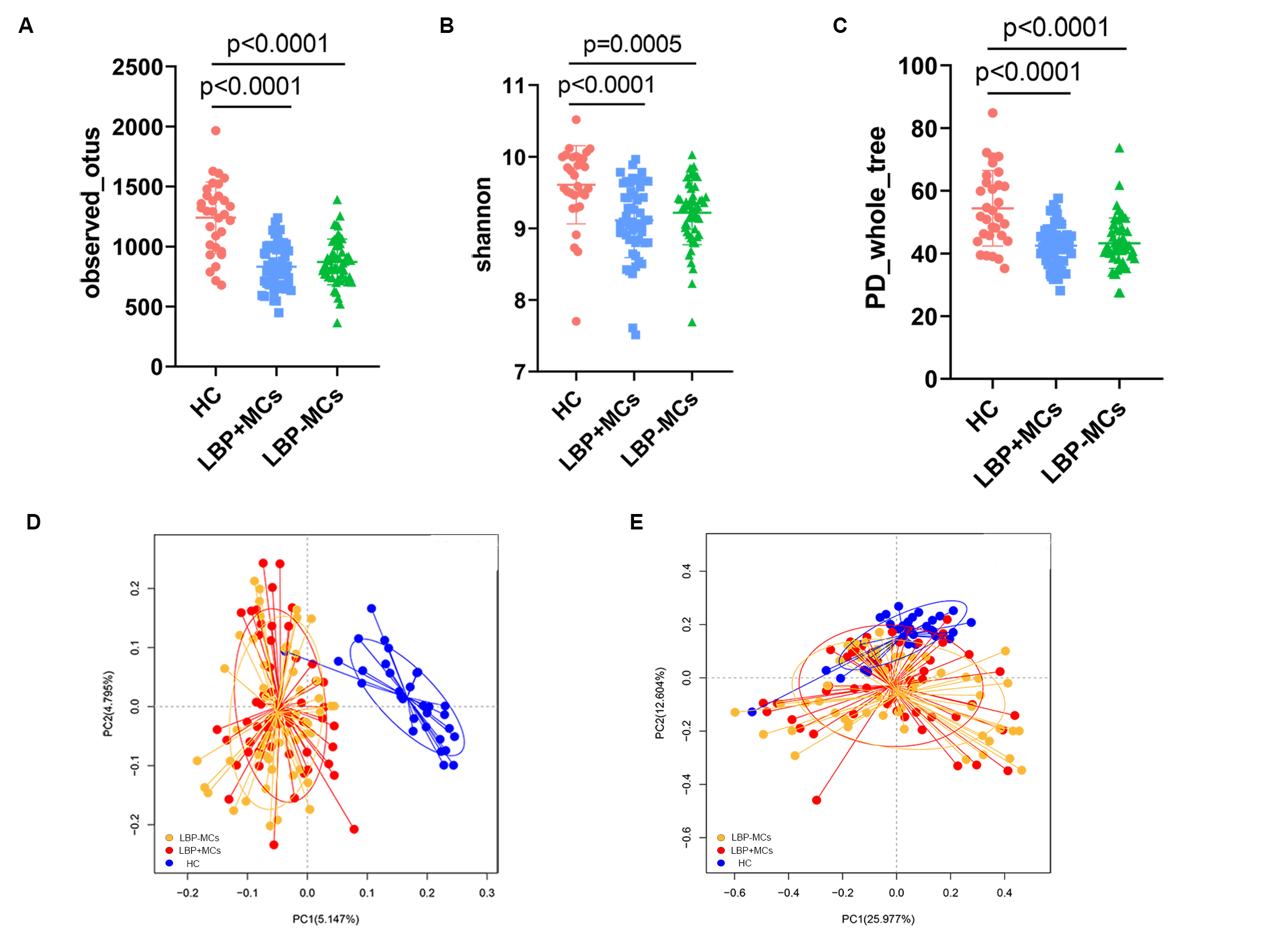
**

**Supplementary Figure 2.** **Alpha diversity indices and Beta diversity of LBP+MCs, LBP-MCs and HC control stool samples**

(A-C) Alpha diversity based on Observed-otus, Shannon’s diversity index and PD-whole-tree index of LBP+MCs, LBP-MCs and HC faecal samples.

(D-E) Beta diversity based on Unweighted_UniFrac and Weighted_Unifrac distance of LBP+MCs, LBP-MCs and HC faecal samples.

The sample size is n=31 HC, n=54 LBP+MCs, n=53 LBP-MCs as biologically independent samples.

**
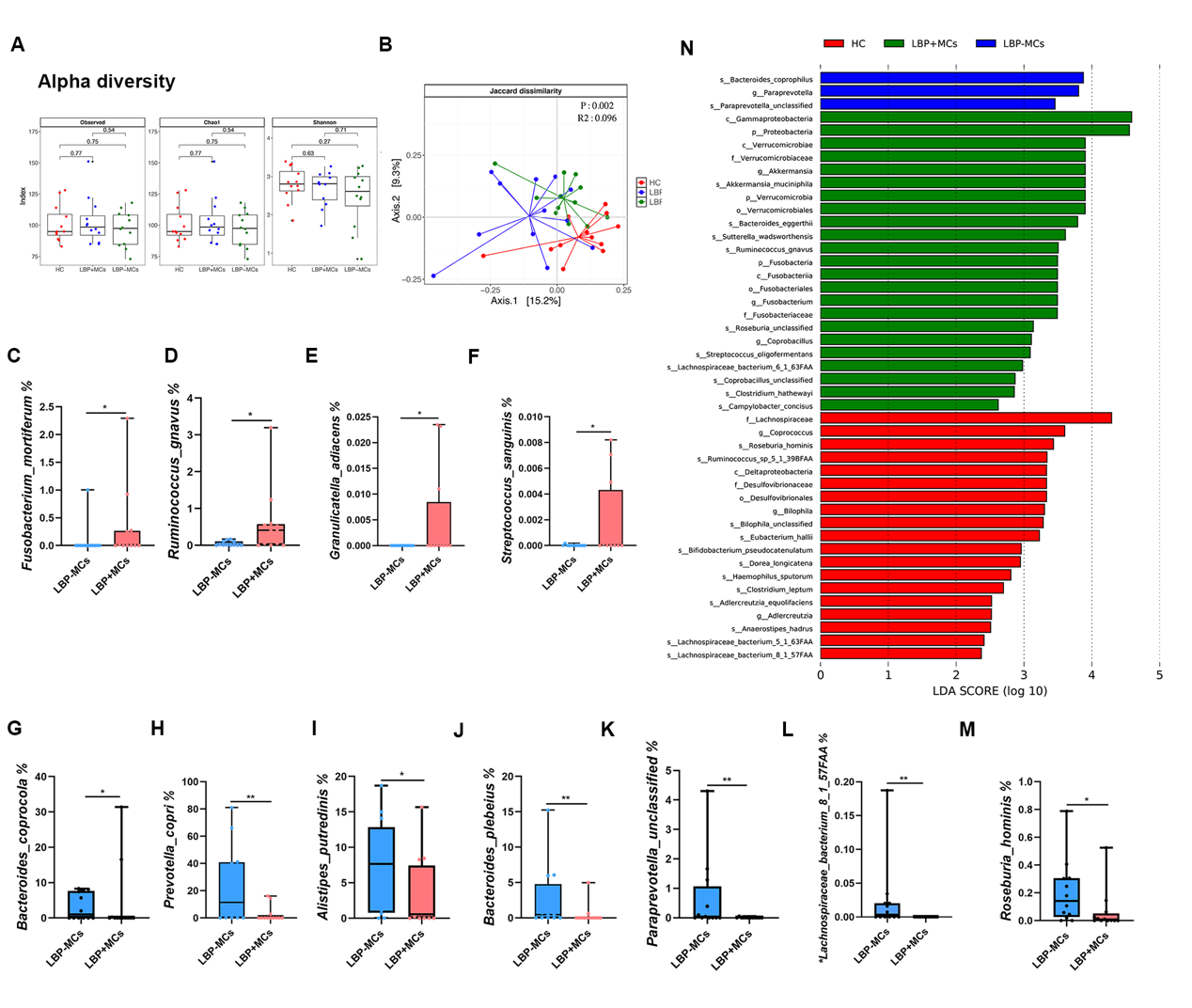
**

**Supplementary Figure 3. Differences in bacterial composition between HC, LBP+MCs and LBP-MCs cohorts.**

(A) Comparison of alpha-diversity indices (Observed-otus, Chao-1-richness index and Shannon’s diversity index) between LBP+MCs, LBP-MCs and HC groups.

(B) Principal coordinate analysis (PCoA) based on Jaccard dissimilarity distances for bacterial sequences between LBP+MCs, LBP-MCs and HC groups.

(C-M) The relative abundances of 11 genera showed significant differences at the species level between LBP+MCs and LBP-MCs groups. * indicates *p* <0.05; ** indicates *p* <0.01; and *** indicates *p* <0.001 by Wilcoxon rank-sum test.

(N) Linear discriminant analysis effect size (LEfSe) analysis identified different taxa between LBP+MCs, LBP-MCs and HC groups. The LDA scores (log_10_) > 2 are listed.


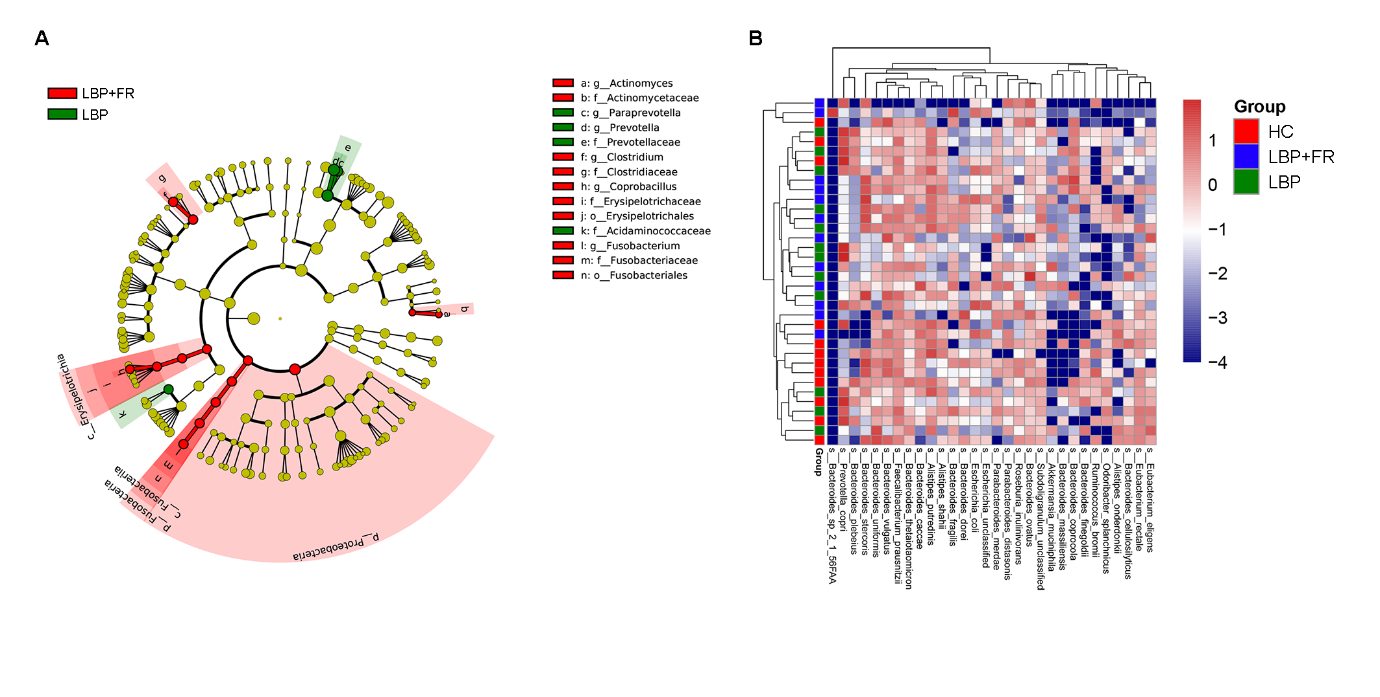


**Supplementary Figure 4. Metagenomic analysis for LBP, LBP+FR and HC groups.**

(A) Linear discriminant analysis effect size identified the most differentially abundant taxa between the LBP+MCs and LBP-MCs groups.

(B) The top 30 abundant species among the three groups.

**
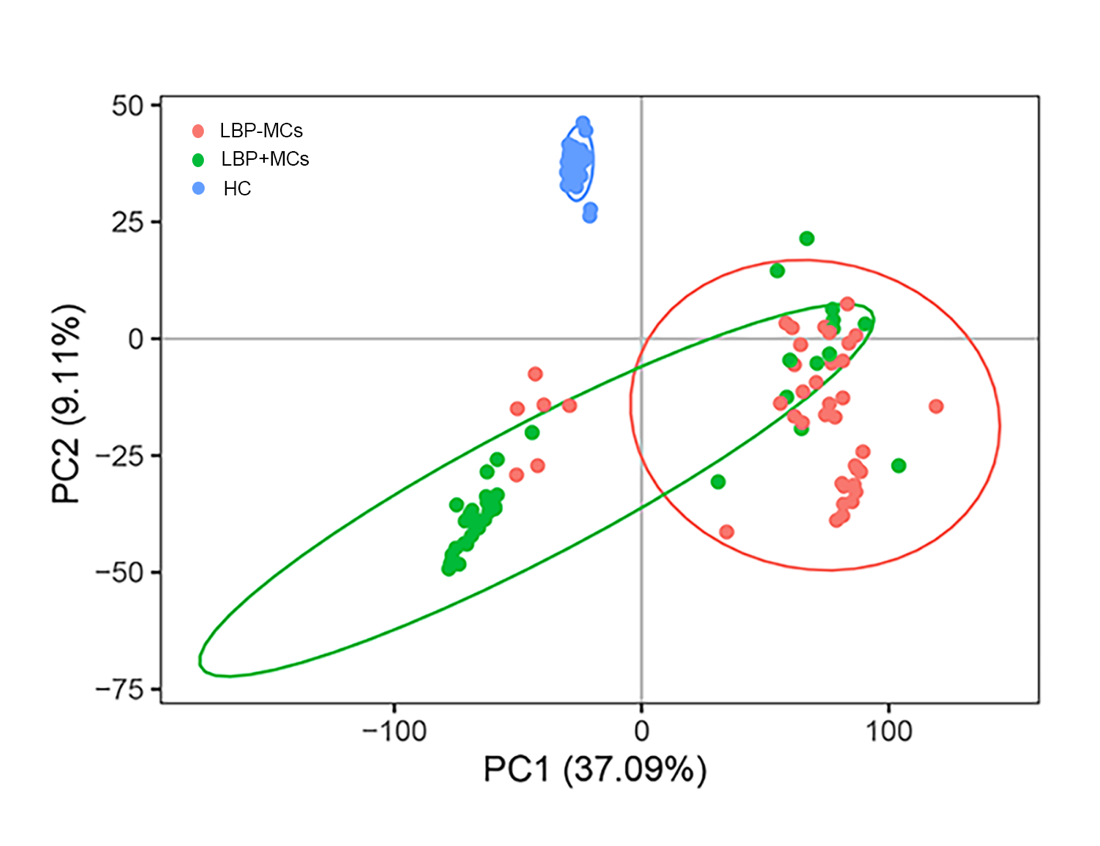
**

**Supplementary Figure 5. Differences in bacterial composition between HC, LBP+MCs and LBP-MCs cohorts.**

The PCA score plot of serum metabolites in HC, LBP+MCs and LBP-MCs groups.

**
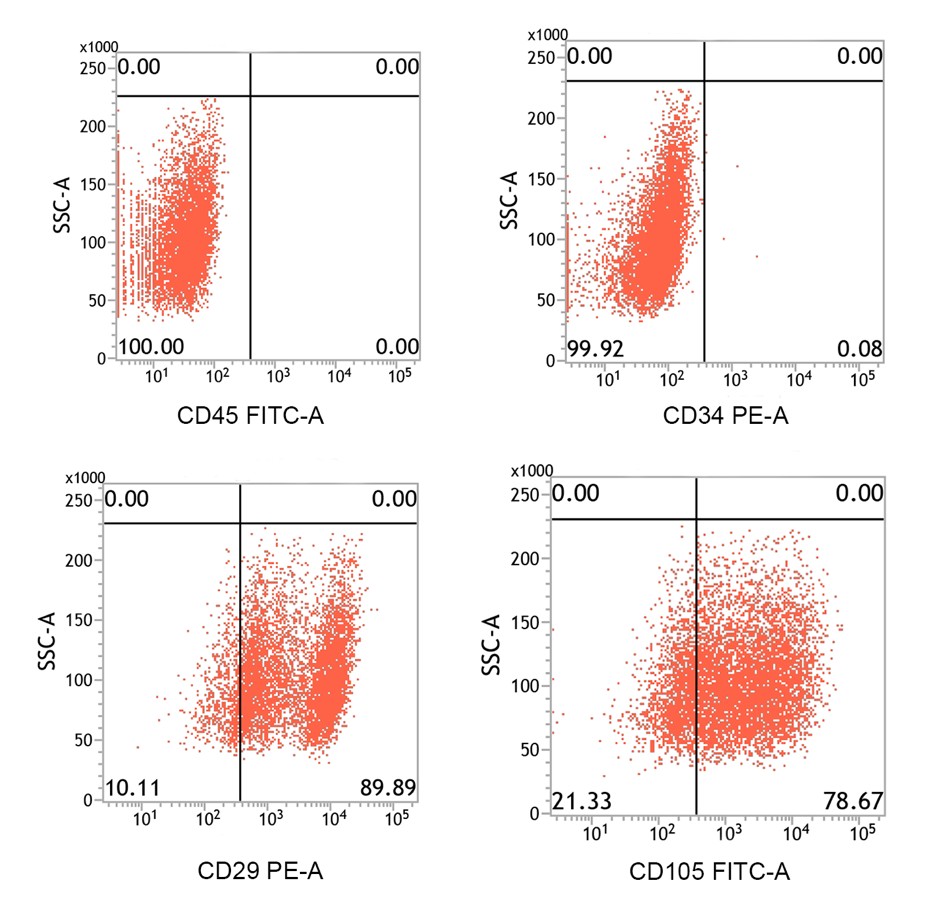
**

**Supplementary Figure 6. Flow identification of BM-MSCs.**

**
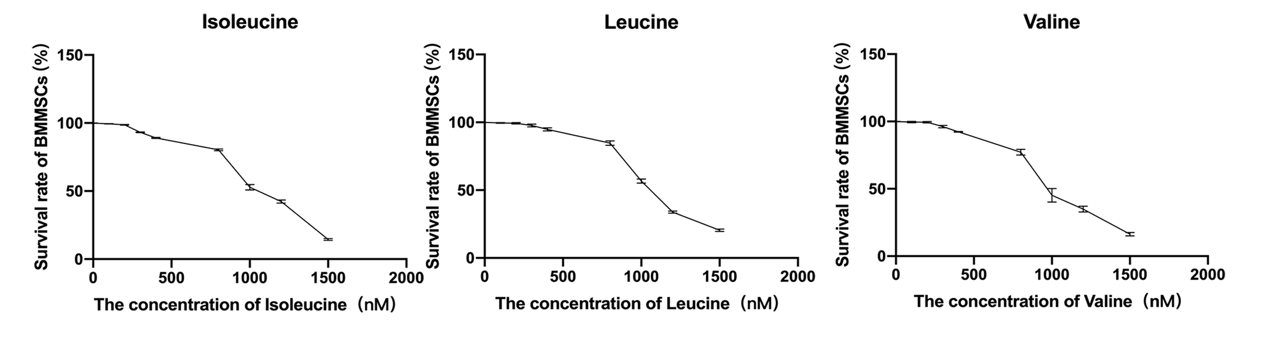
**

**Supplementary Figure 7.** **Increase in BCAA (valine, isoleucine, leucine) dose impacts on BM-MSCs viability in ex vivo model.**

Percentage of viable BM-MSCs, under the different stimulations with three kinds of BCAA demonstrating significant reduction in BM-MSCs viability (< 80%) at different doses. Valine, isoleucine and leucine were prepared to the concentration of 400 nM, 400 nM and 800 nM separately. Data are shown as mean (±SD). BM-MSCs viability was assessed by automated cell counters.

**
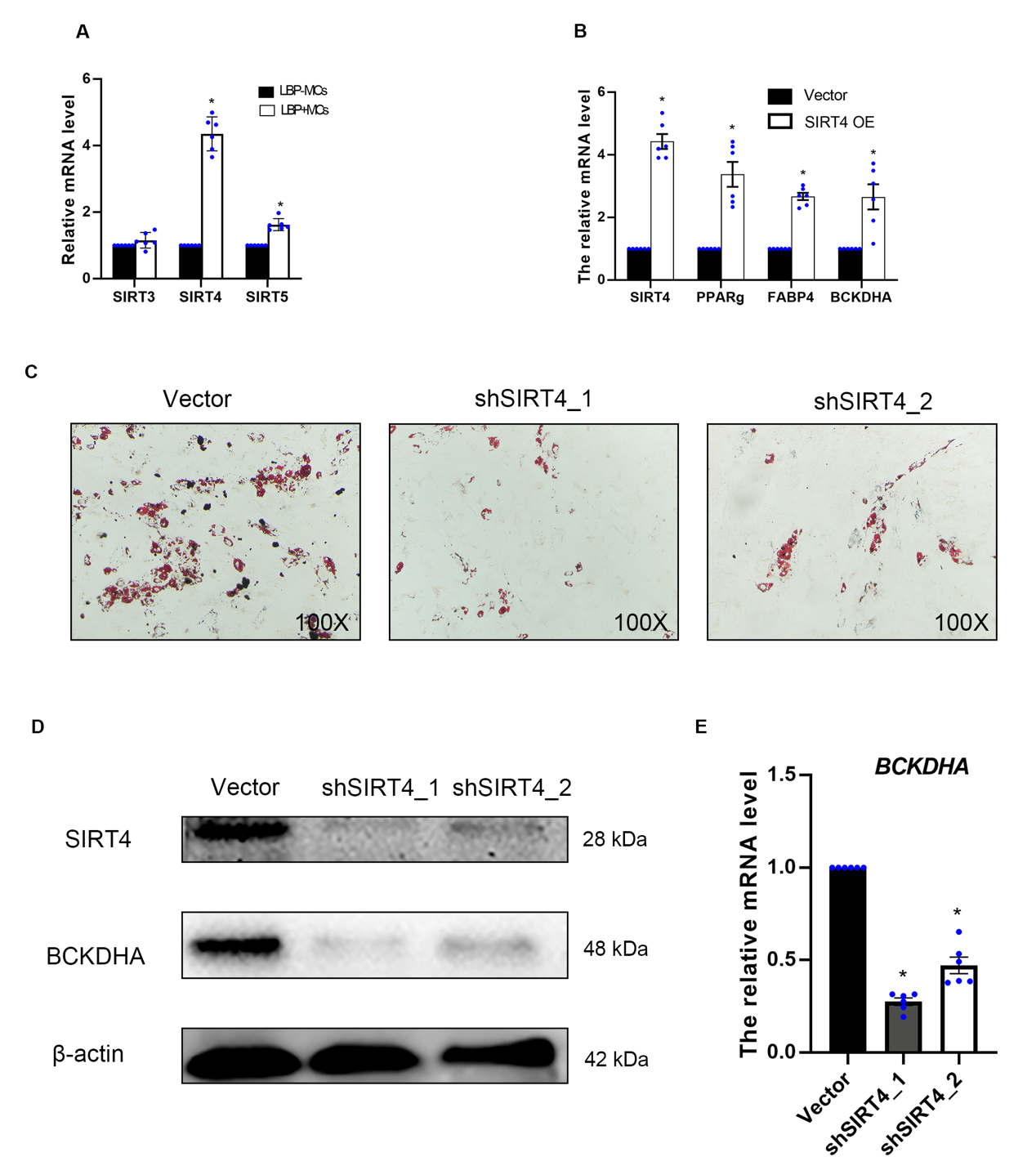
**

**Supplementary Figure 8. SIRT4 boosts adipogenesis on BM-MSCs.**

(A) RT-qPCR analysis results of SIRT3, SIRT4 and SIRT5 in BM-MSCs from LBP+MCs and LBP-MCs group. (**P* < 0.05)

(B) qPCR gene expression analysis of SIRT4, PPARg, FABP4 and BCKDHA in control BM-MSCs or SIRT4-overexpressing BM-MSCs after 14 days of differentiation. (**P* < 0.05)

(C) Representative images of Oil Red O staining of lipids in shcontrol, shSIRT4_1 and shSIRT4_2 BM-MSCs differentiated for 14 days.100× magnification.

(D) Western blot analysis of SIRT4 and BCKDHA in shcontrol, shSIRT4_1 and shSIRT4_2 BM-MSCs differentiated for 14 days.

(E) RT-qPCR analysis results of BCKDHA in shcontrol, shSIRT4_1 and shSIRT4_2 BM-MSCs differentiated for 14 days. (**P* < 0.05)


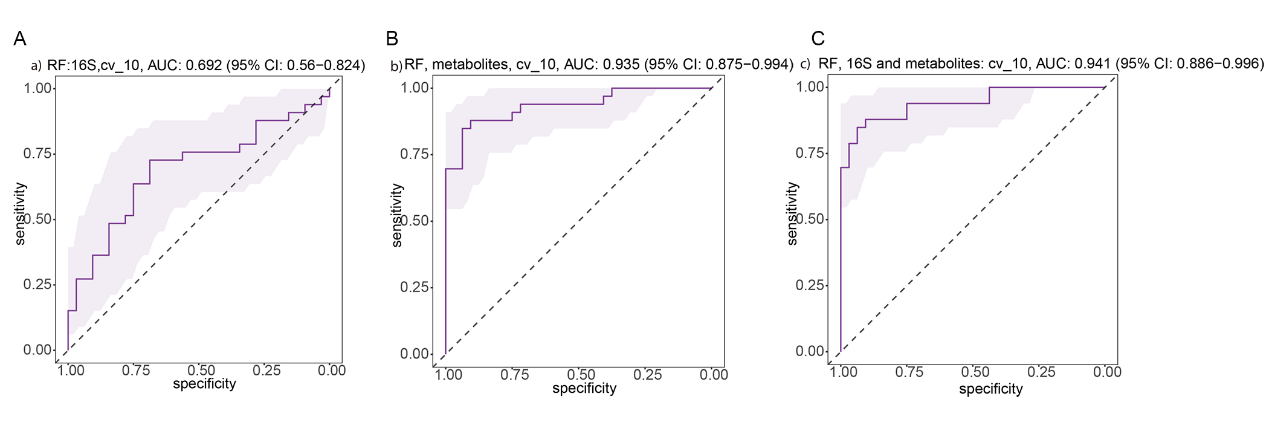


**Supplementary figure 9. Random Forest models to predict LBP different types.**

Random Forest models discriminate the two groups with area under the curve (AUC) ranging from 0.69 to 0.94 (A) all 16S genus, AUC = 0.77; (B) all 10,000 metabolites, AUC = 0.935; (C) combination of all 16S genus and metabolites, AUC = 0.941.

**Supplementary Table 1.** A total of 343 discriminative bacterial species between HC, LBP+MCs and LBP-MCs cohorts.

**Supplementary Table 2. Comparison of relative taxonomic abundance at family and genus level in HC, LBP+MCs and LBP-MCs cohorts.**

Sample size is n=31 HC, n=54 LBP+MCs, n=53 LBP-MCs as biologically independent samples. Data are shown as mean % ± standard error of the mean (SEM). Wilcoxon rank-sum Test calculated *P* values for 2 group comparisons. *P* < 0.05 considered statistically significant.

**Supplementary Table 3.** Comparison of underlying disease-correlated KEGG Orthologies (KOs) between LBP+MCs, LBP-MCs and HC groups.

**Supplementary Table 4.** The top 50 differential fecal metabolites and enriched pathways in serum samples from the LBP+MCs group.

**Supplementary Table 5.** RNA sequencing Detailed results of GO and KEGG enrichment analysis.
