# Supplementary material for: Gut Microbiome and Metabolome Changes in Chronic Low Back Pain Patients With Vertebral Bone Marrow Lesions
Source: JOR Spine. 2025 Jan 27;8(1):e70042. doi: 10.1002/jsp2.70042 (PMC11772216; doi:10.1002/jsp2.70042)
Supplement: Supplementary file 2 — Table S1. A total of 343 discriminative bacterial species between HC, LBP + FR and LBP cohorts. Table S2. Comparison of relative taxonomic abundance at family and genus level in HC, LBP + FR and LBP cohorts. Table S3. Comparison of underlying disease‐correlated KEGG Orthologies (KOs) between LBP + FR, LBP and HC groups. Table S4. The top 50 differential fecal metabolites and enriched pathways in serum samples from the LBP + FR group. Table S5. RNA sequencing Detailed results of GO and KEGG enrichment analysis. Table S6. Characteristics of the study population in Fecal Metagenomic Sequencing Analysis. Table S7. Characteristics of the study population in Fecal Metabolome Analysis. [file JSP2-8-e70042-s002.docx]

**Supplementary table**

**Supplementary Table 1. A total of 343 discriminative bacterial species between HC, LBP+FR and LBP cohorts.**

| **OTU** | **Test-Statistic** | **P** | **FDR_P** | **Bonferroni_P** | **HC_mean** | **LBP+FR_mean** | **LBP_mean** | **taxonomy** |
| --- | --- | --- | --- | --- | --- | --- | --- | --- |
| Seq317798 | 41.61461 | 9.19E-10 | 2.32E-05 | 2.32E-05 | 37.3871 | 7.962963 | 11.54717 | k__Bacteria; p__Firmicutes; c__Clostridia; o__Clostridiales; f__Ruminococcaceae; g__Faecalibacterium; s__prausnitzii |
| Seq305121 | 39.9719 | 2.09E-09 | 2.63E-05 | 5.26E-05 | 48.03226 | 11.92593 | 12.64151 | k__Bacteria; p__Firmicutes; c__Clostridia; o__Clostridiales; f__Ruminococcaceae; g__Faecalibacterium; s__prausnitzii |
| Seq305773 | 38.66106 | 4.03E-09 | 3.38E-05 | 0.000101 | 46.67742 | 10.22222 | 14.24528 | k__Bacteria; p__Firmicutes; c__Clostridia; o__Clostridiales; f__Ruminococcaceae; g__Faecalibacterium; s__prausnitzii |
| Seq282888 | 36.47009 | 1.20E-08 | 5.68E-05 | 0.000303 | 30.12903 | 5.537037 | 12.24528 | k__Bacteria; p__Firmicutes; c__Clostridia; o__Clostridiales; f__Ruminococcaceae; g__Faecalibacterium; s__prausnitzii |
| Seq292620 | 36.43121 | 1.23E-08 | 5.68E-05 | 0.000309 | 31.35484 | 8.648148 | 9.075472 | k__Bacteria; p__Firmicutes; c__Clostridia; o__Clostridiales; f__Ruminococcaceae; g__Faecalibacterium; s__prausnitzii |
| Seq316840 | 36.19376 | 1.38E-08 | 5.68E-05 | 0.000348 | 45.03226 | 10.51852 | 14.90566 | k__Bacteria; p__Firmicutes; c__Clostridia; o__Clostridiales; f__Ruminococcaceae; g__Faecalibacterium; s__prausnitzii |
| Seq309380 | 35.92926 | 1.58E-08 | 5.68E-05 | 0.000397 | 25.09677 | 6.722222 | 8.018868 | k__Bacteria; p__Firmicutes; c__Clostridia; o__Clostridiales; f__Ruminococcaceae; g__Faecalibacterium; s__prausnitzii |
| Seq313915 | 35.29677 | 2.16E-08 | 6.39E-05 | 0.000545 | 36.51613 | 9 | 12.5283 | k__Bacteria; p__Firmicutes; c__Clostridia; o__Clostridiales; f__Ruminococcaceae; g__Faecalibacterium; s__prausnitzii |
| Seq293795 | 35.10271 | 2.39E-08 | 6.39E-05 | 0.000601 | 7.516129 | 76.5 | 73 | k__Bacteria; p__Proteobacteria; c__Gammaproteobacteria; o__Enterobacteriales; f__Enterobacteriaceae; g__Escherichia; s__coli |
| Seq309646 | 34.97983 | 2.54E-08 | 6.39E-05 | 0.000639 | 61.77419 | 13.42593 | 18.81132 | k__Bacteria; p__Firmicutes; c__Clostridia; o__Clostridiales; f__Ruminococcaceae; g__Faecalibacterium; s__prausnitzii |
| Seq323665 | 34.7288 | 2.88E-08 | 6.58E-05 | 0.000724 | 4.967742 | 58.14815 | 48.58491 | k__Bacteria; p__Proteobacteria; c__Gammaproteobacteria; o__Enterobacteriales; f__Enterobacteriaceae; g__Escherichia; s__coli |
| Seq298077 | 33.77108 | 4.64E-08 | 9.74E-05 | 0.001169 | 6.354839 | 68.68519 | 59.32075 | k__Bacteria; p__Proteobacteria; c__Gammaproteobacteria; o__Enterobacteriales; f__Enterobacteriaceae; g__Escherichia; s__coli |
| Seq316232 | 33.26303 | 5.98E-08 | 0.000111 | 0.001507 | 32.80645 | 8.425926 | 12.60377 | k__Bacteria; p__Firmicutes; c__Clostridia; o__Clostridiales; f__Ruminococcaceae; g__Faecalibacterium; s__prausnitzii |
| Seq290017 | 33.19742 | 6.18E-08 | 0.000111 | 0.001557 | 48.16129 | 11.51852 | 15.62264 | k__Bacteria; p__Firmicutes; c__Clostridia; o__Clostridiales; f__Ruminococcaceae; g__Faecalibacterium; s__prausnitzii |
| Seq315250 | 32.80934 | 7.51E-08 | 0.000114 | 0.001891 | 6.516129 | 67.7963 | 61.69811 | k__Bacteria; p__Proteobacteria; c__Gammaproteobacteria; o__Enterobacteriales; f__Enterobacteriaceae; g__Escherichia; s__coli |
| Seq296300 | 32.77867 | 7.62E-08 | 0.000114 | 0.00192 | 37.58065 | 7.425926 | 14.45283 | k__Bacteria; p__Firmicutes; c__Clostridia; o__Clostridiales; f__Ruminococcaceae; g__Faecalibacterium; s__prausnitzii |
| Seq296330 | 32.38328 | 9.29E-08 | 0.000114 | 0.00234 | 38.12903 | 9.111111 | 11.66038 | k__Bacteria; p__Firmicutes; c__Clostridia; o__Clostridiales; f__Ruminococcaceae; g__Faecalibacterium; s__prausnitzii |
| Seq304352 | 32.23488 | 1.00E-07 | 0.000114 | 0.00252 | 5.387097 | 65.72222 | 54.67925 | k__Bacteria; p__Proteobacteria; c__Gammaproteobacteria; o__Enterobacteriales; f__Enterobacteriaceae; g__Escherichia; s__coli |
| Seq311845 | 32.15445 | 1.04E-07 | 0.000114 | 0.002623 | 6.870968 | 62.14815 | 49.62264 | k__Bacteria; p__Proteobacteria; c__Gammaproteobacteria; o__Enterobacteriales; f__Enterobacteriaceae; g__Escherichia; s__coli |
| Seq329487 | 32.12237 | 1.06E-07 | 0.000114 | 0.002666 | 6.83871 | 62.81481 | 49.45283 | k__Bacteria; p__Proteobacteria; c__Gammaproteobacteria; o__Enterobacteriales; f__Enterobacteriaceae; g__Escherichia; s__coli |
| Seq304964 | 32.06565 | 1.09E-07 | 0.000114 | 0.002742 | 4.806452 | 52.72222 | 47.77358 | k__Bacteria; p__Proteobacteria; c__Gammaproteobacteria; o__Enterobacteriales; f__Enterobacteriaceae; g__Escherichia; s__coli |
| Seq312826 | 32.01433 | 1.12E-07 | 0.000114 | 0.002814 | 7.096774 | 63.94444 | 55.92453 | k__Bacteria; p__Proteobacteria; c__Gammaproteobacteria; o__Enterobacteriales; f__Enterobacteriaceae; g__Escherichia; s__coli |
| Seq310979 | 31.86382 | 1.20E-07 | 0.000114 | 0.003033 | 11.70968 | 89.62963 | 79.90566 | k__Bacteria; p__Proteobacteria; c__Gammaproteobacteria; o__Enterobacteriales; f__Enterobacteriaceae; g__Escherichia; s__coli |
| Seq288321 | 31.84925 | 1.21E-07 | 0.000114 | 0.003056 | 37.87097 | 9.87037 | 11.69811 | k__Bacteria; p__Firmicutes; c__Clostridia; o__Clostridiales; f__Ruminococcaceae; g__Faecalibacterium; s__prausnitzii |
| Seq285261 | 31.81988 | 1.23E-07 | 0.000114 | 0.003101 | 35.6129 | 8.777778 | 11.32075 | k__Bacteria; p__Firmicutes; c__Clostridia; o__Clostridiales; f__Ruminococcaceae; g__Faecalibacterium; s__prausnitzii |
| Seq319189 | 31.81147 | 1.24E-07 | 0.000114 | 0.003114 | 25.6129 | 5.777778 | 10.15094 | k__Bacteria; p__Firmicutes; c__Clostridia; o__Clostridiales; f__Ruminococcaceae; g__Faecalibacterium; s__prausnitzii |
| Seq303973 | 31.66734 | 1.33E-07 | 0.000114 | 0.003347 | 5.419355 | 63.53704 | 48.39623 | k__Bacteria; p__Proteobacteria; c__Gammaproteobacteria; o__Enterobacteriales; f__Enterobacteriaceae; g__Escherichia; s__coli |
| Seq308098 | 31.55619 | 1.40E-07 | 0.000114 | 0.003538 | 49.48387 | 11 | 14.77358 | k__Bacteria; p__Firmicutes; c__Clostridia; o__Clostridiales; f__Ruminococcaceae; g__Faecalibacterium; s__prausnitzii |
| Seq321655 | 31.53832 | 1.42E-07 | 0.000114 | 0.00357 | 9 | 77.5 | 69.66038 | k__Bacteria; p__Proteobacteria; c__Gammaproteobacteria; o__Enterobacteriales; f__Enterobacteriaceae; g__Escherichia; s__coli |
| Seq286104 | 31.51185 | 1.44E-07 | 0.000114 | 0.003617 | 6.16129 | 54.64815 | 48.16981 | k__Bacteria; p__Proteobacteria; c__Gammaproteobacteria; o__Enterobacteriales; f__Enterobacteriaceae; g__Escherichia; s__coli |
| Seq308817 | 31.47631 | 1.46E-07 | 0.000114 | 0.003682 | 34.67742 | 8.962963 | 12.49057 | k__Bacteria; p__Firmicutes; c__Clostridia; o__Clostridiales; f__Ruminococcaceae; g__Faecalibacterium; s__prausnitzii |
| Seq316782 | 31.41136 | 1.51E-07 | 0.000114 | 0.003803 | 45.19355 | 11.35185 | 15.49057 | k__Bacteria; p__Firmicutes; c__Clostridia; o__Clostridiales; f__Ruminococcaceae; g__Faecalibacterium; s__prausnitzii |
| Seq292891 | 31.40396 | 1.52E-07 | 0.000114 | 0.003818 | 45.45161 | 12.46296 | 16.54717 | k__Bacteria; p__Firmicutes; c__Clostridia; o__Clostridiales; f__Ruminococcaceae; g__Faecalibacterium; s__prausnitzii |
| Seq291590 | 31.38194 | 1.53E-07 | 0.000114 | 0.00386 | 7.258065 | 0 | 1.018868 | k__Bacteria; p__Firmicutes; c__Clostridia; o__Clostridiales; f__Lachnospiraceae; g__; s__ |
| Seq326799 | 31.21597 | 1.67E-07 | 0.000116 | 0.004194 | 28.80645 | 6.740741 | 10.9434 | k__Bacteria; p__Firmicutes; c__Clostridia; o__Clostridiales; f__Ruminococcaceae; g__Faecalibacterium; s__prausnitzii |
| Seq287878 | 31.17917 | 1.70E-07 | 0.000116 | 0.004272 | 9.870968 | 73.27778 | 66.86792 | k__Bacteria; p__Proteobacteria; c__Gammaproteobacteria; o__Enterobacteriales; f__Enterobacteriaceae; g__Escherichia; s__coli |
| Seq293837 | 31.16932 | 1.70E-07 | 0.000116 | 0.004293 | 6.096774 | 66.5 | 53.0566 | k__Bacteria; p__Proteobacteria; c__Gammaproteobacteria; o__Enterobacteriales; f__Enterobacteriaceae; g__Escherichia; s__coli |
| Seq324122 | 30.85796 | 1.99E-07 | 0.000132 | 0.005016 | 37 | 9.5 | 13.20755 | k__Bacteria; p__Firmicutes; c__Clostridia; o__Clostridiales; f__Ruminococcaceae; g__Faecalibacterium; s__prausnitzii |
| Seq315237 | 30.75605 | 2.10E-07 | 0.000135 | 0.005278 | 11.19355 | 2 | 0.943396 | k__Bacteria; p__Firmicutes; c__Clostridia; o__Clostridiales; f__Lachnospiraceae; g__; s__ |
| Seq303197 | 30.62184 | 2.24E-07 | 0.000138 | 0.005644 | 6.16129 | 62.85185 | 57.28302 | k__Bacteria; p__Proteobacteria; c__Gammaproteobacteria; o__Enterobacteriales; f__Enterobacteriaceae; g__Escherichia; s__coli |
| Seq307898 | 30.54504 | 2.33E-07 | 0.000138 | 0.005865 | 7.516129 | 75.77778 | 65.13208 | k__Bacteria; p__Proteobacteria; c__Gammaproteobacteria; o__Enterobacteriales; f__Enterobacteriaceae; g__Escherichia; s__coli |
| Seq308822 | 30.52695 | 2.35E-07 | 0.000138 | 0.005919 | 32.58065 | 9.018519 | 13.15094 | k__Bacteria; p__Firmicutes; c__Clostridia; o__Clostridiales; f__Ruminococcaceae; g__Faecalibacterium; s__prausnitzii |
| Seq305845 | 30.50814 | 2.37E-07 | 0.000138 | 0.005975 | 24.41935 | 5.907407 | 10.37736 | k__Bacteria; p__Firmicutes; c__Clostridia; o__Clostridiales; f__Ruminococcaceae; g__Faecalibacterium; s__prausnitzii |
| Seq310306 | 30.46692 | 2.42E-07 | 0.000138 | 0.006099 | 13.51613 | 0.962963 | 1.943396 | k__Bacteria; p__Firmicutes; c__Clostridia; o__Clostridiales; f__Lachnospiraceae; g__; s__ |
| Seq285801 | 30.42631 | 2.47E-07 | 0.000138 | 0.006224 | 8.580645 | 75.96296 | 63.39623 | k__Bacteria; p__Proteobacteria; c__Gammaproteobacteria; o__Enterobacteriales; f__Enterobacteriaceae; g__Escherichia; s__coli |
| Seq318412 | 30.15895 | 2.83E-07 | 0.000152 | 0.007114 | 7.645161 | 0.648148 | 1.396226 | k__Bacteria; p__Firmicutes; c__Clostridia; o__Clostridiales; f__Lachnospiraceae; g__; s__ |
| Seq293948 | 30.1552 | 2.83E-07 | 0.000152 | 0.007128 | 4.612903 | 1.203704 | 1.113208 | k__Bacteria; p__Firmicutes; c__Clostridia; o__Clostridiales; f__Lachnospiraceae; g__Lachnospira; s__ |
| Seq299365 | 30.08366 | 2.93E-07 | 0.000153 | 0.007387 | 4.935484 | 56.87037 | 46.60377 | k__Bacteria; p__Proteobacteria; c__Gammaproteobacteria; o__Enterobacteriales; f__Enterobacteriaceae; g__Escherichia; s__coli |
| Seq315616 | 30.05666 | 2.97E-07 | 0.000153 | 0.007488 | 35.32258 | 8.796296 | 10.43396 | k__Bacteria; p__Firmicutes; c__Clostridia; o__Clostridiales; f__Ruminococcaceae; g__Faecalibacterium; s__prausnitzii |
| Seq317975 | 29.88147 | 3.25E-07 | 0.000161 | 0.008173 | 8.193548 | 81.48148 | 66.5283 | k__Bacteria; p__Proteobacteria; c__Gammaproteobacteria; o__Enterobacteriales; f__Enterobacteriaceae; g__Escherichia; s__coli |
| Seq295603 | 29.86455 | 3.27E-07 | 0.000161 | 0.008243 | 9.419355 | 2.203704 | 4.320755 | k__Bacteria; p__Firmicutes; c__Clostridia; o__Clostridiales; f__Lachnospiraceae; g__Lachnospira; s__ |
| Seq284658 | 29.831 | 3.33E-07 | 0.000161 | 0.008382 | 15.51613 | 1.074074 | 2.792453 | k__Bacteria; p__Firmicutes; c__Clostridia; o__Clostridiales; f__Lachnospiraceae; g__; s__ |
| Seq287546 | 29.44597 | 4.04E-07 | 0.000192 | 0.010162 | 28.83871 | 8.444444 | 10.0566 | k__Bacteria; p__Firmicutes; c__Clostridia; o__Clostridiales; f__Ruminococcaceae; g__Faecalibacterium; s__prausnitzii |
| Seq291655 | 29.33899 | 4.26E-07 | 0.000195 | 0.01072 | 13.70968 | 3 | 1.188679 | k__Bacteria; p__Firmicutes; c__Clostridia; o__Clostridiales; f__Lachnospiraceae; g__; s__ |
| Seq316810 | 29.33706 | 4.26E-07 | 0.000195 | 0.01073 | 7.16129 | 67.64815 | 52.84906 | k__Bacteria; p__Proteobacteria; c__Gammaproteobacteria; o__Enterobacteriales; f__Enterobacteriaceae; g__Escherichia; s__coli |
| Seq294088 | 29.24587 | 4.46E-07 | 0.000201 | 0.011231 | 33.93548 | 8.574074 | 11.50943 | k__Bacteria; p__Firmicutes; c__Clostridia; o__Clostridiales; f__Ruminococcaceae; g__Faecalibacterium; s__prausnitzii |
| Seq329211 | 29.06573 | 4.88E-07 | 0.000213 | 0.012289 | 8.677419 | 0 | 0 | k__Bacteria; p__Firmicutes; c__Clostridia; o__Clostridiales; f__Lachnospiraceae; g__Clostridium; s__colinum |
| Seq292014 | 29.05756 | 4.90E-07 | 0.000213 | 0.01234 | 8.709677 | 2.092593 | 0.301887 | k__Bacteria; p__Firmicutes; c__Clostridia; o__Clostridiales; f__Lachnospiraceae; g__Lachnospira; s__ |
| Seq308591 | 28.81014 | 5.55E-07 | 0.000237 | 0.013965 | 8.967742 | 74.59259 | 62.13208 | k__Bacteria; p__Proteobacteria; c__Gammaproteobacteria; o__Enterobacteriales; f__Enterobacteriaceae; g__Escherichia; s__coli |
| Seq300398 | 28.71381 | 5.82E-07 | 0.000242 | 0.014654 | 8.16129 | 77.7037 | 59.58491 | k__Bacteria; p__Proteobacteria; c__Gammaproteobacteria; o__Enterobacteriales; f__Enterobacteriaceae; g__Escherichia; s__coli |
| Seq324387 | 28.69519 | 5.87E-07 | 0.000242 | 0.014791 | 8.096774 | 65.72222 | 55.18868 | k__Bacteria; p__Proteobacteria; c__Gammaproteobacteria; o__Enterobacteriales; f__Enterobacteriaceae; g__Escherichia; s__coli |
| Seq328301 | 28.57421 | 6.24E-07 | 0.000251 | 0.015713 | 6.032258 | 56.68519 | 42.73585 | k__Bacteria; p__Proteobacteria; c__Gammaproteobacteria; o__Enterobacteriales; f__Enterobacteriaceae; g__Escherichia; s__coli |
| Seq319271 | 28.55774 | 6.29E-07 | 0.000251 | 0.015843 | 6.741935 | 63.90741 | 53.75472 | k__Bacteria; p__Proteobacteria; c__Gammaproteobacteria; o__Enterobacteriales; f__Enterobacteriaceae; g__Escherichia; s__coli |
| Seq303440 | 28.40885 | 6.78E-07 | 0.000267 | 0.017068 | 8.387097 | 75.09259 | 64.84906 | k__Bacteria; p__Proteobacteria; c__Gammaproteobacteria; o__Enterobacteriales; f__Enterobacteriaceae; g__Escherichia; s__coli |
| Seq319413 | 28.16971 | 7.64E-07 | 0.000296 | 0.019235 | 3.967742 | 0.203704 | 0 | k__Bacteria; p__Firmicutes; c__Clostridia; o__Clostridiales; f__Lachnospiraceae; g__; s__ |
| Seq307150 | 28.13046 | 7.79E-07 | 0.000297 | 0.019616 | 6.83871 | 0 | 2.490566 | k__Bacteria; p__Firmicutes; c__Clostridia; o__Clostridiales; f__Lachnospiraceae; g__; s__ |
| Seq317906 | 27.99644 | 8.33E-07 | 0.000313 | 0.020976 | 38.3871 | 10.11111 | 15.11321 | k__Bacteria; p__Firmicutes; c__Clostridia; o__Clostridiales; f__Ruminococcaceae; g__Faecalibacterium; s__prausnitzii |
| Seq316945 | 27.73645 | 9.49E-07 | 0.000351 | 0.023888 | 6.967742 | 0.796296 | 0.150943 | k__Bacteria; p__Firmicutes; c__Clostridia; o__Clostridiales; f__Lachnospiraceae; g__; s__ |
| Seq309607 | 27.62645 | 1.00E-06 | 0.000366 | 0.025239 | 7.129032 | 0.203704 | 0.698113 | k__Bacteria; p__Firmicutes; c__Clostridia; o__Clostridiales; f__Lachnospiraceae; g__; s__ |
| Seq301136 | 27.59179 | 1.02E-06 | 0.000367 | 0.02568 | 4.774194 | 0.888889 | 0 | k__Bacteria; p__Firmicutes; c__Clostridia; o__Clostridiales; f__Lachnospiraceae; g__; s__ |
| Seq319325 | 27.44633 | 1.10E-06 | 0.000388 | 0.027617 | 24.48387 | 7.5 | 8.415094 | k__Bacteria; p__Firmicutes; c__Clostridia; o__Clostridiales; f__Ruminococcaceae; g__Faecalibacterium; s__prausnitzii |
| Seq298623 | 27.42495 | 1.11E-06 | 0.000388 | 0.027914 | 12.74194 | 3.185185 | 0.830189 | k__Bacteria; p__Firmicutes; c__Clostridia; o__Clostridiales; f__Ruminococcaceae; g__Oscillospira; s__ |
| Seq310480 | 27.30943 | 1.17E-06 | 0.000405 | 0.029574 | 10.19355 | 1.814815 | 4.075472 | k__Bacteria; p__Firmicutes; c__Clostridia; o__Clostridiales; f__Lachnospiraceae; g__Lachnospira; s__ |
| Seq329384 | 27.05867 | 1.33E-06 | 0.000453 | 0.033524 | 5.677419 | 57.98148 | 49.50943 | k__Bacteria; p__Proteobacteria; c__Gammaproteobacteria; o__Enterobacteriales; f__Enterobacteriaceae; g__Escherichia; s__coli |
| Seq313973 | 26.9776 | 1.39E-06 | 0.000465 | 0.034911 | 7.870968 | 2.055556 | 1.169811 | k__Bacteria; p__Firmicutes; c__Clostridia; o__Clostridiales; f__Lachnospiraceae; g__; s__ |
| Seq326659 | 26.9532 | 1.40E-06 | 0.000465 | 0.035339 | 47.35484 | 12.35185 | 19.11321 | k__Bacteria; p__Firmicutes; c__Clostridia; o__Clostridiales; f__Ruminococcaceae; g__Faecalibacterium; s__prausnitzii |
| Seq302853 | 26.89465 | 1.45E-06 | 0.000473 | 0.036389 | 13.54839 | 1.481481 | 2.528302 | k__Bacteria; p__Firmicutes; c__Clostridia; o__Clostridiales; f__Lachnospiraceae; g__; s__ |
| Seq312897 | 26.83411 | 1.49E-06 | 0.00048 | 0.037508 | 4.258065 | 52.83333 | 38.62264 | k__Bacteria; p__Proteobacteria; c__Gammaproteobacteria; o__Enterobacteriales; f__Enterobacteriaceae; g__Escherichia; s__coli |
| Seq316620 | 26.79632 | 1.52E-06 | 0.00048 | 0.038223 | 5.580645 | 64.62963 | 48.5283 | k__Bacteria; p__Proteobacteria; c__Gammaproteobacteria; o__Enterobacteriales; f__Enterobacteriaceae; g__Escherichia; s__coli |
| Seq318439 | 26.78913 | 1.52E-06 | 0.00048 | 0.038361 | 7.258065 | 2.555556 | 1.056604 | k__Bacteria; p__Firmicutes; c__Clostridia; o__Clostridiales; f__Lachnospiraceae; g__; s__ |
| Seq310605 | 26.68698 | 1.60E-06 | 0.000498 | 0.040371 | 6.580645 | 1.277778 | 1.301887 | k__Bacteria; p__Firmicutes; c__Clostridia; o__Clostridiales; f__Lachnospiraceae; g__; s__ |
| Seq310146 | 26.48072 | 1.78E-06 | 0.000546 | 0.044757 | 7.870968 | 1.833333 | 1.471698 | k__Bacteria; p__Firmicutes; c__Clostridia; o__Clostridiales; f__Lachnospiraceae; g__; s__ |
| Seq308454 | 26.44387 | 1.81E-06 | 0.000549 | 0.045589 | 10.93548 | 0.925926 | 5.433962 | k__Bacteria; p__Firmicutes; c__Clostridia; o__Clostridiales; f__Lachnospiraceae; g__Roseburia; s__ |
| Seq317064 | 26.41616 | 1.84E-06 | 0.00055 | 0.046225 | 6.548387 | 0.592593 | 3.773585 | k__Bacteria; p__Firmicutes; c__Clostridia; o__Clostridiales; f__Lachnospiraceae; g__; s__ |
| Seq305785 | 26.35464 | 1.89E-06 | 0.000558 | 0.047669 | 35.51613 | 10.88889 | 15.67925 | k__Bacteria; p__Firmicutes; c__Clostridia; o__Clostridiales; f__Ruminococcaceae; g__Faecalibacterium; s__prausnitzii |
| Seq307469 | 26.34126 | 1.91E-06 | 0.000558 | 0.047989 | 5.225806 | 1.37037 | 4.566038 | k__Bacteria; p__Firmicutes; c__Clostridia; o__Clostridiales; f__Lachnospiraceae; g__Lachnospira; s__ |
| Seq291588 | 25.93222 | 2.34E-06 | 0.000677 | 0.058879 | 8 | 64.85185 | 55.90566 | k__Bacteria; p__Proteobacteria; c__Gammaproteobacteria; o__Enterobacteriales; f__Enterobacteriaceae; g__Escherichia; s__coli |
| Seq293295 | 25.32796 | 3.16E-06 | 0.000905 | 0.079648 | 5.290323 | 48.31481 | 41.43396 | k__Bacteria; p__Proteobacteria; c__Gammaproteobacteria; o__Enterobacteriales; f__Enterobacteriaceae; g__Escherichia; s__coli |
| Seq307374 | 25.24597 | 3.30E-06 | 0.000905 | 0.082981 | 4.451613 | 0 | 0 | k__Bacteria; p__Firmicutes; c__Clostridia; o__Clostridiales; f__Lachnospiraceae; g__; s__ |
| Seq313540 | 25.24557 | 3.30E-06 | 0.000905 | 0.082998 | 4.129032 | 0 | 0 | k__Bacteria; p__Firmicutes; c__Clostridia; o__Clostridiales; f__Lachnospiraceae; g__; s__ |
| Seq296055 | 25.24557 | 3.30E-06 | 0.000905 | 0.082998 | 4.967742 | 0 | 0 | k__Bacteria; p__Firmicutes; c__Clostridia; o__Clostridiales; f__Lachnospiraceae; g__Lachnospira; s__ |
| Seq293242 | 25.23866 | 3.31E-06 | 0.000905 | 0.083285 | 34.16129 | 10.48148 | 13.24528 | k__Bacteria; p__Firmicutes; c__Clostridia; o__Clostridiales; f__Ruminococcaceae; g__Faecalibacterium; s__prausnitzii |
| Seq314491 | 25.03004 | 3.67E-06 | 0.000994 | 0.092442 | 11.67742 | 0.333333 | 2.150943 | k__Bacteria; p__Firmicutes; c__Clostridia; o__Clostridiales; f__Lachnospiraceae; g__; s__ |
| Seq293121 | 24.9115 | 3.90E-06 | 0.001043 | 0.098086 | 10.22581 | 67.2963 | 58.73585 | k__Bacteria; p__Proteobacteria; c__Gammaproteobacteria; o__Enterobacteriales; f__Enterobacteriaceae; g__Escherichia; s__coli |
| Seq283413 | 24.87938 | 3.96E-06 | 0.001049 | 0.099674 | 4.645161 | 49.48148 | 40.37736 | k__Bacteria; p__Proteobacteria; c__Gammaproteobacteria; o__Enterobacteriales; f__Enterobacteriaceae; g__Escherichia; s__coli |
| Seq316091 | 24.77575 | 4.17E-06 | 0.001093 | 0.104975 | 27.09677 | 8.111111 | 12.33962 | k__Bacteria; p__Firmicutes; c__Clostridia; o__Clostridiales; f__Ruminococcaceae; g__Faecalibacterium; s__prausnitzii |
| Seq315518 | 24.55541 | 4.65E-06 | 0.001208 | 0.117202 | 11.19355 | 77.5 | 70.50943 | k__Bacteria; p__Proteobacteria; c__Gammaproteobacteria; o__Enterobacteriales; f__Enterobacteriaceae; g__Escherichia; s__coli |
| Seq296727 | 24.32057 | 5.23E-06 | 0.001345 | 0.131804 | 11.48387 | 1.703704 | 2.45283 | k__Bacteria; p__Firmicutes; c__Clostridia; o__Clostridiales; f__Lachnospiraceae; g__; s__ |
| Seq297608 | 24.22596 | 5.49E-06 | 0.001387 | 0.138188 | 23.03226 | 2.425926 | 6.528302 | k__Bacteria; p__Firmicutes; c__Clostridia; o__Clostridiales; f__Lachnospiraceae; g__Roseburia; s__ |
| Seq316101 | 24.21917 | 5.51E-06 | 0.001387 | 0.138658 | 6.064516 | 0 | 0.471698 | k__Bacteria; p__Firmicutes; c__Clostridia; o__Clostridiales; f__Lachnospiraceae; g__; s__ |
| Seq309701 | 24.16556 | 5.66E-06 | 0.00141 | 0.142425 | 5.806452 | 0 | 0.830189 | k__Bacteria; p__Firmicutes; c__Clostridia; o__Clostridiales; f__Lachnospiraceae; g__; s__ |
| Seq323739 | 24.10697 | 5.82E-06 | 0.001438 | 0.14666 | 9.387097 | 0 | 1 | k__Bacteria; p__Firmicutes; c__Clostridia; o__Clostridiales; f__Lachnospiraceae; g__Clostridium; s__colinum |
| Seq302728 | 24.08303 | 5.89E-06 | 0.001441 | 0.148426 | 17.03226 | 3.055556 | 3.45283 | k__Bacteria; p__Firmicutes; c__Clostridia; o__Clostridiales; f__Lachnospiraceae; g__; s__ |
| Seq299797 | 24.05322 | 5.98E-06 | 0.001449 | 0.150655 | 6.451613 | 0.481481 | 0.471698 | k__Bacteria; p__Firmicutes; c__Clostridia; o__Clostridiales; f__Lachnospiraceae; g__; s__ |
| Seq290407 | 23.9215 | 6.39E-06 | 0.001509 | 0.160911 | 7.451613 | 0 | 1.830189 | k__Bacteria; p__Firmicutes; c__Clostridia; o__Clostridiales; f__Lachnospiraceae; g__; s__ |
| Seq317605 | 23.88359 | 6.51E-06 | 0.001509 | 0.16399 | 5 | 0 | 2.018868 | k__Bacteria; p__Firmicutes; c__Clostridia; o__Clostridiales; f__Lachnospiraceae; g__; s__ |
| Seq284541 | 23.87872 | 6.53E-06 | 0.001509 | 0.16439 | 3.612903 | 1.074074 | 0 | k__Bacteria; p__Firmicutes; c__Clostridia; o__Clostridiales; f__Lachnospiraceae; g__Dorea; s__ |
| Seq314593 | 23.87753 | 6.53E-06 | 0.001509 | 0.164487 | 5.064516 | 0.814815 | 0 | k__Bacteria; p__Firmicutes; c__Clostridia; o__Clostridiales; f__Lachnospiraceae; g__Dorea; s__ |
| Seq309869 | 23.87753 | 6.53E-06 | 0.001509 | 0.164487 | 4.354839 | 1.407407 | 0 | k__Bacteria; p__Firmicutes; c__Clostridia; o__Clostridiales; f__Lachnospiraceae; g__; s__ |
| Seq306252 | 23.8279 | 6.70E-06 | 0.001527 | 0.168621 | 54.41935 | 9.462963 | 20.98113 | k__Bacteria; p__Firmicutes; c__Clostridia; o__Clostridiales; f__Ruminococcaceae; g__Faecalibacterium; s__prausnitzii |
| Seq296733 | 23.81752 | 6.73E-06 | 0.001527 | 0.169498 | 13.29032 | 6.537037 | 0.433962 | k__Bacteria; p__Firmicutes; c__Clostridia; o__Clostridiales; f__Lachnospiraceae; g__; s__ |
| Seq284832 | 23.75821 | 6.93E-06 | 0.001559 | 0.1746 | 42.48387 | 14.51852 | 14.96226 | k__Bacteria; p__Firmicutes; c__Clostridia; o__Clostridiales; f__Ruminococcaceae; g__Faecalibacterium; s__prausnitzii |
| Seq304308 | 23.73244 | 7.02E-06 | 0.001565 | 0.176864 | 5.741935 | 54.40741 | 45.71698 | k__Bacteria; p__Proteobacteria; c__Gammaproteobacteria; o__Enterobacteriales; f__Enterobacteriaceae |
| Seq327872 | 23.6795 | 7.21E-06 | 0.001593 | 0.181608 | 5.225806 | 0 | 1.490566 | k__Bacteria; p__Firmicutes; c__Clostridia; o__Clostridiales; f__Lachnospiraceae; g__; s__ |
| Seq310968 | 23.51731 | 7.82E-06 | 0.001713 | 0.196949 | 8.290323 | 2.12963 | 0.849057 | k__Bacteria; p__Firmicutes; c__Clostridia; o__Clostridiales; f__Lachnospiraceae; g__; s__ |
| Seq304551 | 23.31123 | 8.67E-06 | 0.001882 | 0.218325 | 41.54839 | 8.111111 | 12.77358 | k__Bacteria; p__Firmicutes; c__Clostridia; o__Clostridiales; f__Ruminococcaceae; g__Faecalibacterium; s__prausnitzii |
| Seq284082 | 23.17418 | 9.29E-06 | 0.001998 | 0.23381 | 14.58065 | 2.055556 | 5.754717 | k__Bacteria; p__Firmicutes; c__Clostridia; o__Clostridiales; f__Lachnospiraceae; g__Roseburia; s__ |
| Seq327237 | 23.12363 | 9.52E-06 | 0.002032 | 0.239795 | 6.322581 | 58.92593 | 50.09434 | k__Bacteria; p__Proteobacteria; c__Gammaproteobacteria; o__Enterobacteriales; f__Enterobacteriaceae; g__Escherichia; s__coli |
| Seq287831 | 23.00025 | 1.01E-05 | 0.002143 | 0.255054 | 3.516129 | 1.166667 | 0.811321 | k__Bacteria; p__Firmicutes; c__Clostridia; o__Clostridiales; f__Lachnospiraceae; g__; s__ |
| Seq314828 | 22.84178 | 1.10E-05 | 0.002286 | 0.276085 | 5.677419 | 3.351852 | 1.584906 | k__Bacteria; p__Firmicutes; c__Clostridia; o__Clostridiales; f__Lachnospiraceae; g__; s__ |
| Seq307420 | 22.83808 | 1.10E-05 | 0.002286 | 0.276597 | 4.548387 | 1.5 | 0.735849 | k__Bacteria; p__Firmicutes; c__Clostridia; o__Clostridiales; f__Lachnospiraceae; g__Lachnospira; s__ |
| Seq303006 | 22.7384 | 1.15E-05 | 0.002383 | 0.290732 | 15.87097 | 2.018519 | 2.075472 | k__Bacteria; p__Firmicutes; c__Clostridia; o__Clostridiales; f__Lachnospiraceae; g__; s__ |
| Seq321779 | 22.01985 | 1.65E-05 | 0.003367 | 0.416412 | 17.77419 | 1.944444 | 4.660377 | k__Bacteria; p__Firmicutes; c__Clostridia; o__Clostridiales; f__Lachnospiraceae; g__Roseburia; s__ |
| Seq318101 | 22.01431 | 1.66E-05 | 0.003367 | 0.417568 | 5.870968 | 2.12963 | 1.660377 | k__Bacteria; p__Firmicutes; c__Clostridia; o__Clostridiales; f__Lachnospiraceae; g__Lachnospira; s__ |
| Seq328651 | 21.67307 | 1.97E-05 | 0.003962 | 0.495252 | 9.354839 | 2.296296 | 2.339623 | k__Bacteria; p__Firmicutes; c__Clostridia; o__Clostridiales; f__Lachnospiraceae; g__; s__ |
| Seq283325 | 21.52712 | 2.12E-05 | 0.004228 | 0.532745 | 13.80645 | 3.703704 | 8.735849 | k__Bacteria; p__Firmicutes; c__Clostridia; o__Clostridiales; f__Lachnospiraceae; g__Roseburia; s__faecis |
| Seq317535 | 21.48038 | 2.17E-05 | 0.00426 | 0.545341 | 3.870968 | 0 | 0 | k__Bacteria; p__Firmicutes; c__Clostridia; o__Clostridiales; f__Lachnospiraceae; g__Lachnospira; s__ |
| Seq286788 | 21.48038 | 2.17E-05 | 0.00426 | 0.545341 | 3.709677 | 0 | 0 | k__Bacteria; p__Firmicutes; c__Clostridia; o__Clostridiales; f__Lachnospiraceae; g__Lachnospira; s__ |
| Seq318306 | 21.46066 | 2.19E-05 | 0.004269 | 0.550744 | 13.25806 | 2.518519 | 3.037736 | k__Bacteria; p__Firmicutes; c__Clostridia; o__Clostridiales; f__Lachnospiraceae; g__; s__ |
| Seq286864 | 21.41867 | 2.23E-05 | 0.004326 | 0.562428 | 13.70968 | 5.351852 | 2.679245 | k__Bacteria; p__Firmicutes; c__Clostridia; o__Clostridiales; f__Lachnospiraceae; g__; s__ |
| Seq304093 | 21.3654 | 2.29E-05 | 0.004409 | 0.57761 | 4.580645 | 1.592593 | 1.641509 | k__Bacteria; p__Firmicutes; c__Clostridia; o__Clostridiales; f__Lachnospiraceae; g__Lachnospira; s__ |
| Seq289366 | 21.31086 | 2.36E-05 | 0.004466 | 0.593579 | 13.45161 | 4.259259 | 5.679245 | k__Bacteria; p__Firmicutes; c__Clostridia; o__Clostridiales; f__Lachnospiraceae; g__Roseburia; s__faecis |
| Seq300564 | 21.30962 | 2.36E-05 | 0.004466 | 0.593947 | 31.64516 | 9.851852 | 15.86792 | k__Bacteria; p__Firmicutes; c__Clostridia; o__Clostridiales; f__Ruminococcaceae; g__Faecalibacterium; s__prausnitzii |
| Seq318661 | 21.24603 | 2.43E-05 | 0.004576 | 0.613136 | 9.290323 | 0.888889 | 1.981132 | k__Bacteria; p__Firmicutes; c__Clostridia; o__Clostridiales; f__Lachnospiraceae; g__; s__ |
| Seq312966 | 21.14588 | 2.56E-05 | 0.004768 | 0.644618 | 4.322581 | 39.53704 | 37.50943 | k__Bacteria; p__Proteobacteria; c__Gammaproteobacteria; o__Enterobacteriales; f__Enterobacteriaceae |
| Seq327272 | 21.12158 | 2.59E-05 | 0.004768 | 0.652498 | 41.48387 | 10.51852 | 16.39623 | k__Bacteria; p__Firmicutes; c__Clostridia; o__Clostridiales; f__Ruminococcaceae; g__Faecalibacterium; s__prausnitzii |
| Seq318115 | 21.1195 | 2.59E-05 | 0.004768 | 0.653179 | 5.483871 | 4.962963 | 0.245283 | k__Bacteria; p__Firmicutes; c__Clostridia; o__Clostridiales; f__Lachnospiraceae; g__Lachnospira; s__ |
| Seq318739 | 21.02253 | 2.72E-05 | 0.004968 | 0.685629 | 29.67742 | 7.018519 | 11.92453 | k__Bacteria; p__Firmicutes; c__Clostridia; o__Clostridiales; f__Ruminococcaceae; g__Faecalibacterium; s__prausnitzii |
| Seq314201 | 20.96073 | 2.81E-05 | 0.005087 | 0.707145 | 6.548387 | 0 | 0.698113 | k__Bacteria; p__Firmicutes; c__Clostridia; o__Clostridiales; f__Lachnospiraceae; g__; s__ |
| Seq313319 | 20.94537 | 2.83E-05 | 0.00509 | 0.712595 | 5.580645 | 0.166667 | 0 | k__Bacteria; p__Firmicutes; c__Clostridia; o__Clostridiales; f__Ruminococcaceae; g__Butyricicoccus; s__pullicaecorum |
| Seq306058 | 20.84838 | 2.97E-05 | 0.005274 | 0.748006 | 2.83871 | 39.01852 | 37.79245 | k__Bacteria; p__Proteobacteria; c__Gammaproteobacteria; o__Enterobacteriales; f__Enterobacteriaceae |
| Seq324248 | 20.84581 | 2.97E-05 | 0.005274 | 0.748966 | 8.677419 | 0.740741 | 0 | k__Bacteria; p__Firmicutes; c__Clostridia; o__Clostridiales; f__Lachnospiraceae; g__Clostridium; s__colinum |
| Seq293471 | 20.77242 | 3.09E-05 | 0.005433 | 0.77696 | 5.483871 | 0 | 1.056604 | k__Bacteria; p__Firmicutes; c__Clostridia; o__Clostridiales; f__Lachnospiraceae; g__Dorea; s__formicigenerans |
| Seq326200 | 20.62411 | 3.32E-05 | 0.005811 | 0.836768 | 2.258065 | 34.07407 | 28.35849 | k__Bacteria; p__Proteobacteria; c__Gammaproteobacteria; o__Enterobacteriales; f__Enterobacteriaceae |
| Seq314289 | 20.60262 | 3.36E-05 | 0.005833 | 0.845805 | 10.16129 | 1.814815 | 0.320755 | k__Bacteria; p__Firmicutes; c__Clostridia; o__Clostridiales; f__Lachnospiraceae; g__Clostridium; s__colinum |
| Seq307475 | 20.52776 | 3.49E-05 | 0.005983 | 0.878063 | 25.80645 | 3.222222 | 6.698113 | k__Bacteria; p__Firmicutes; c__Clostridia; o__Clostridiales; f__Lachnospiraceae; g__Roseburia; s__ |
| Seq327441 | 20.52438 | 3.49E-05 | 0.005983 | 0.87955 | 5.354839 | 1.962963 | 1.54717 | k__Bacteria; p__Firmicutes; c__Clostridia; o__Clostridiales; f__Lachnospiraceae; g__Lachnospira; s__ |
| Seq286588 | 20.49307 | 3.55E-05 | 0.006037 | 0.893425 | 25.64516 | 10.05556 | 11.35849 | k__Bacteria; p__Firmicutes; c__Clostridia; o__Clostridiales; f__Ruminococcaceae; g__Faecalibacterium; s__prausnitzii |
| Seq329005 | 20.45569 | 3.61E-05 | 0.006075 | 0.910285 | 2.967742 | 0.333333 | 0 | k__Bacteria; p__Bacteroidetes; c__Bacteroidia; o__Bacteroidales; f__Bacteroidaceae; g__Bacteroides; s__plebeius |
| Seq315837 | 20.45341 | 3.62E-05 | 0.006075 | 0.911322 | 4.064516 | 0.425926 | 0 | k__Bacteria; p__Bacteroidetes; c__Bacteroidia; o__Bacteroidales; f__Bacteroidaceae; g__Bacteroides; s__plebeius |
| Seq287773 | 20.38743 | 3.74E-05 | 0.006238 | 0.941888 | 6.774194 | 1.981481 | 0.226415 | k__Bacteria; p__Firmicutes; c__Clostridia; o__Clostridiales; f__Ruminococcaceae; g__Butyricicoccus; s__pullicaecorum |
| Seq307608 | 20.35133 | 3.81E-05 | 0.00629 | 0.959043 | 6.064516 | 0 | 1.679245 | k__Bacteria; p__Firmicutes; c__Clostridia; o__Clostridiales; f__Lachnospiraceae; g__; s__ |
| Seq328467 | 20.34452 | 3.82E-05 | 0.00629 | 0.962315 | 5.645161 | 1.203704 | 0 | k__Bacteria; p__Firmicutes; c__Clostridia; o__Clostridiales; f__Lachnospiraceae; g__; s__ |
| Seq293291 | 20.26801 | 3.97E-05 | 0.006468 | 0.999842 | 2.677419 | 40.40741 | 32.32075 | k__Bacteria; p__Proteobacteria; c__Gammaproteobacteria; o__Enterobacteriales; f__Enterobacteriaceae |
| Seq307645 | 20.2398 | 4.03E-05 | 0.006468 | 1 | 12.19355 | 5.574074 | 4.924528 | k__Bacteria; p__Firmicutes; c__Clostridia; o__Clostridiales; f__Lachnospiraceae; g__Roseburia; s__faecis |
| Seq314637 | 20.23714 | 4.03E-05 | 0.006468 | 1 | 2.419355 | 0.648148 | 0 | k__Bacteria; p__Firmicutes; c__Clostridia; o__Clostridiales; f__Lachnospiraceae; g__; s__ |
| Seq327077 | 20.23686 | 4.03E-05 | 0.006468 | 1 | 2.548387 | 0.648148 | 0 | k__Bacteria; p__Firmicutes; c__Clostridia; o__Clostridiales; f__Lachnospiraceae; g__; s__ |
| Seq317503 | 20.20422 | 4.10E-05 | 0.006533 | 1 | 8.451613 | 0.259259 | 0.660377 | k__Bacteria; p__Firmicutes; c__Clostridia; o__Clostridiales; f__Lachnospiraceae; g__Clostridium; s__colinum |
| Seq323272 | 20.13031 | 4.25E-05 | 0.006737 | 1 | 20.41935 | 3.592593 | 5.283019 | k__Bacteria; p__Firmicutes; c__Clostridia; o__Clostridiales; f__Lachnospiraceae; g__Roseburia; s__ |
| Seq323464 | 20.10158 | 4.32E-05 | 0.006754 | 1 | 5.580645 | 0.62963 | 0.339623 | k__Bacteria; p__Firmicutes; c__Clostridia; o__Clostridiales; f__Lachnospiraceae; g__; s__ |
| Seq307944 | 20.10026 | 4.32E-05 | 0.006754 | 1 | 4.16129 | 1.574074 | 0 | k__Bacteria; p__Firmicutes; c__Clostridia; o__Clostridiales; f__Lachnospiraceae; g__Dorea; s__ |
| Seq291152 | 20.03765 | 4.46E-05 | 0.006925 | 1 | 4.935484 | 2.166667 | 2.45283 | k__Bacteria; p__Firmicutes; c__Clostridia; o__Clostridiales; f__Lachnospiraceae; g__Lachnospira; s__ |
| Seq284820 | 20.0007 | 4.54E-05 | 0.00693 | 1 | 2.870968 | 0.740741 | 0.188679 | k__Bacteria; p__Firmicutes; c__Clostridia; o__Clostridiales; f__Lachnospiraceae; g__Dorea; s__ |
| Seq325274 | 19.99976 | 4.54E-05 | 0.00693 | 1 | 3.83871 | 0.240741 | 0.415094 | k__Bacteria; p__Firmicutes; c__Clostridia; o__Clostridiales; f__Lachnospiraceae; g__Dorea; s__ |
| Seq283537 | 19.99948 | 4.54E-05 | 0.00693 | 1 | 5.483871 | 0.259259 | 0.509434 | k__Bacteria; p__Firmicutes; c__Clostridia; o__Clostridiales; f__Lachnospiraceae; g__; s__ |
| Seq282967 | 19.9478 | 4.66E-05 | 0.007069 | 1 | 10.45161 | 3.722222 | 5.415094 | k__Bacteria; p__Firmicutes; c__Clostridia; o__Clostridiales; f__Lachnospiraceae; g__Roseburia; s__faecis |
| Seq324831 | 19.92057 | 4.72E-05 | 0.007123 | 1 | 5.064516 | 2.592593 | 3.169811 | k__Bacteria; p__Firmicutes; c__Clostridia; o__Clostridiales; f__Lachnospiraceae; g__; s__ |
| Seq314318 | 19.87572 | 4.83E-05 | 0.007241 | 1 | 15.83871 | 4.148148 | 3.037736 | k__Bacteria; p__Firmicutes; c__Clostridia; o__Clostridiales; f__Lachnospiraceae; g__Roseburia; s__ |
| Seq289582 | 19.73665 | 5.18E-05 | 0.007717 | 1 | 38.48387 | 7.518519 | 17.67925 | k__Bacteria; p__Firmicutes; c__Clostridia; o__Clostridiales; f__Ruminococcaceae; g__Faecalibacterium; s__prausnitzii |
| Seq325596 | 19.6004 | 5.54E-05 | 0.008174 | 1 | 13.03226 | 3.092593 | 2.169811 | k__Bacteria; p__Firmicutes; c__Clostridia; o__Clostridiales; f__Lachnospiraceae; g__; s__ |
| Seq311729 | 19.59392 | 5.56E-05 | 0.008174 | 1 | 15.67742 | 1.425926 | 6.150943 | k__Bacteria; p__Firmicutes; c__Clostridia; o__Clostridiales; f__Lachnospiraceae; g__Roseburia; s__ |
| Seq288338 | 19.58617 | 5.58E-05 | 0.008174 | 1 | 3.451613 | 0.796296 | 1.320755 | k__Bacteria; p__Firmicutes; c__Clostridia; o__Clostridiales; f__Lachnospiraceae; g__Lachnospira; s__ |
| Seq287801 | 19.55155 | 5.68E-05 | 0.008269 | 1 | 10.25806 | 3.351852 | 6.339623 | k__Bacteria; p__Firmicutes; c__Clostridia; o__Clostridiales; f__Lachnospiraceae; g__Roseburia; s__faecis |
| Seq329287 | 19.38087 | 6.19E-05 | 0.008954 | 1 | 14.32258 | 3.314815 | 4.849057 | k__Bacteria; p__Firmicutes; c__Clostridia; o__Clostridiales; f__Lachnospiraceae; g__; s__ |
| Seq291440 | 19.3315 | 6.34E-05 | 0.009125 | 1 | 13.16129 | 10.59259 | 37.86792 | k__Bacteria; p__Bacteroidetes; c__Bacteroidia; o__Bacteroidales; f__Bacteroidaceae; g__Bacteroides; s__caccae |
| Seq327846 | 19.23106 | 6.67E-05 | 0.009493 | 1 | 11.96774 | 2.944444 | 4.773585 | k__Bacteria; p__Firmicutes; c__Clostridia; o__Clostridiales; f__Lachnospiraceae; g__; s__ |
| Seq310559 | 19.22982 | 6.67E-05 | 0.009493 | 1 | 15.54839 | 6.907407 | 3.509434 | k__Bacteria; p__Firmicutes; c__Clostridia; o__Clostridiales; f__Lachnospiraceae; g__; s__ |
| Seq294136 | 19.21659 | 6.72E-05 | 0.009495 | 1 | 5.451613 | 1.37037 | 0.490566 | k__Bacteria; p__Firmicutes; c__Clostridia; o__Clostridiales; f__Ruminococcaceae; g__Oscillospira; s__ |
| Seq286244 | 19.20683 | 6.75E-05 | 0.009495 | 1 | 3.16129 | 41.25926 | 34.30189 | k__Bacteria; p__Proteobacteria; c__Gammaproteobacteria; o__Enterobacteriales; f__Enterobacteriaceae |
| Seq286966 | 19.12839 | 7.02E-05 | 0.009753 | 1 | 15.58065 | 8.685185 | 2.566038 | k__Bacteria; p__Firmicutes; c__Clostridia; o__Clostridiales; f__Lachnospiraceae; g__; s__ |
| Seq294851 | 19.12727 | 7.02E-05 | 0.009753 | 1 | 7.645161 | 3.388889 | 0.830189 | k__Bacteria; p__Firmicutes; c__Clostridia; o__Clostridiales; f__Lachnospiraceae; g__Lachnospira; s__ |
| Seq324785 | 19.12007 | 7.05E-05 | 0.009753 | 1 | 52.87097 | 14.31481 | 22.01887 | k__Bacteria; p__Firmicutes; c__Clostridia; o__Clostridiales; f__Ruminococcaceae; g__Faecalibacterium; s__prausnitzii |
| Seq299907 | 19.04335 | 7.32E-05 | 0.010079 | 1 | 8.322581 | 3.018519 | 4.245283 | k__Bacteria; p__Firmicutes; c__Clostridia; o__Clostridiales; f__Lachnospiraceae; g__Roseburia; s__faecis |
| Seq319249 | 18.99395 | 7.51E-05 | 0.010275 | 1 | 14.19355 | 2.333333 | 3.773585 | k__Bacteria; p__Firmicutes; c__Clostridia; o__Clostridiales; f__Lachnospiraceae; g__Roseburia; s__ |
| Seq320822 | 18.94466 | 7.70E-05 | 0.010474 | 1 | 12.45161 | 4.574074 | 4.924528 | k__Bacteria; p__Firmicutes; c__Clostridia; o__Clostridiales; f__Lachnospiraceae; g__Roseburia |
| Seq328618 | 18.89731 | 7.88E-05 | 0.010667 | 1 | 10.77419 | 1.425926 | 4.226415 | k__Bacteria; p__Firmicutes; c__Clostridia; o__Clostridiales; f__Lachnospiraceae; g__; s__ |
| Seq327427 | 18.84586 | 8.08E-05 | 0.010887 | 1 | 10.54839 | 2.333333 | 2.679245 | k__Bacteria; p__Firmicutes; c__Clostridia; o__Clostridiales; f__Lachnospiraceae; g__Roseburia; s__ |
| Seq297405 | 18.82337 | 8.18E-05 | 0.010951 | 1 | 10.80645 | 2.407407 | 2.113208 | k__Bacteria; p__Firmicutes; c__Clostridia; o__Clostridiales; f__Lachnospiraceae; g__; s__ |
| Seq292015 | 18.79011 | 8.31E-05 | 0.011021 | 1 | 11.77419 | 2 | 3.245283 | k__Bacteria; p__Firmicutes; c__Clostridia; o__Clostridiales; f__Lachnospiraceae; g__; s__ |
| Seq303164 | 18.78947 | 8.32E-05 | 0.011021 | 1 | 5.548387 | 3.833333 | 0.566038 | k__Bacteria; p__Firmicutes; c__Clostridia; o__Clostridiales; f__Lachnospiraceae; g__Lachnospira; s__ |
| Seq290688 | 18.76665 | 8.41E-05 | 0.01109 | 1 | 12 | 3.462963 | 3.226415 | k__Bacteria; p__Firmicutes; c__Clostridia; o__Clostridiales; f__Lachnospiraceae; g__; s__ |
| Seq322024 | 18.74891 | 8.49E-05 | 0.011126 | 1 | 48.6129 | 11.51852 | 19.88679 | k__Bacteria; p__Firmicutes; c__Clostridia; o__Clostridiales; f__Ruminococcaceae; g__Faecalibacterium; s__prausnitzii |
| Seq295183 | 18.73921 | 8.53E-05 | 0.011126 | 1 | 7.193548 | 0.888889 | 0.792453 | k__Bacteria; p__Firmicutes; c__Clostridia; o__Clostridiales; f__Lachnospiraceae; g__Lachnospira; s__ |
| Seq314703 | 18.70206 | 8.69E-05 | 0.011276 | 1 | 17.74194 | 4.722222 | 7.679245 | k__Bacteria; p__Firmicutes; c__Clostridia; o__Clostridiales; f__Lachnospiraceae; g__; s__ |
| Seq326122 | 18.46829 | 9.76E-05 | 0.01261 | 1 | 67 | 14.77778 | 21.56604 | k__Bacteria; p__Firmicutes; c__Clostridia; o__Clostridiales; f__Ruminococcaceae; g__Faecalibacterium; s__prausnitzii |
| Seq289579 | 18.40212 | 0.000101 | 0.012967 | 1 | 13.70968 | 3.537037 | 4.056604 | k__Bacteria; p__Firmicutes; c__Clostridia; o__Clostridiales; f__Lachnospiraceae; g__; s__ |
| Seq308510 | 18.36947 | 0.000103 | 0.013114 | 1 | 7.967742 | 0 | 1.09434 | k__Bacteria; p__Firmicutes; c__Clostridia; o__Clostridiales; f__Lachnospiraceae; g__Clostridium; s__colinum |
| Seq309809 | 18.27775 | 0.000107 | 0.01366 | 1 | 3.096774 | 39.57407 | 30.58491 | k__Bacteria; p__Proteobacteria; c__Gammaproteobacteria; o__Enterobacteriales; f__Enterobacteriaceae |
| Seq314214 | 18.25729 | 0.000109 | 0.013705 | 1 | 15.93548 | 6.259259 | 7.849057 | k__Bacteria; p__Firmicutes; c__Clostridia; o__Clostridiales; f__Lachnospiraceae; g__Roseburia; s__faecis |
| Seq289347 | 18.25103 | 0.000109 | 0.013705 | 1 | 15.25806 | 7.444444 | 7.056604 | k__Bacteria; p__Firmicutes; c__Clostridia; o__Clostridiales; f__Lachnospiraceae; g__Roseburia; s__faecis |
| Seq313282 | 18.21122 | 0.000111 | 0.013839 | 1 | 6.096774 | 1.611111 | 0.056604 | k__Bacteria; p__Firmicutes; c__Clostridia; o__Clostridiales; f__Lachnospiraceae; g__Dorea; s__ |
| Seq306920 | 18.20657 | 0.000111 | 0.013839 | 1 | 7.580645 | 1.351852 | 1.566038 | k__Bacteria; p__Firmicutes; c__Clostridia; o__Clostridiales; f__Lachnospiraceae; g__; s__ |
| Seq301665 | 18.20184 | 0.000112 | 0.013839 | 1 | 13.09677 | 3.611111 | 5.283019 | k__Bacteria; p__Firmicutes; c__Clostridia; o__Clostridiales; f__Lachnospiraceae; g__; s__ |
| Seq288524 | 18.1737 | 0.000113 | 0.013966 | 1 | 2.064516 | 30.92593 | 25.37736 | k__Bacteria; p__Proteobacteria; c__Gammaproteobacteria; o__Enterobacteriales; f__Enterobacteriaceae; g__Klebsiella; s__ |
| Seq321723 | 18.1296 | 0.000116 | 0.014208 | 1 | 5.903226 | 1.12963 | 0 | k__Bacteria; p__Firmicutes; c__Clostridia; o__Clostridiales; f__Lachnospiraceae; g__Dorea; s__ |
| Seq288890 | 18.01846 | 0.000122 | 0.014947 | 1 | 5.290323 | 2.111111 | 2.603774 | k__Bacteria; p__Firmicutes; c__Clostridia; o__Clostridiales; f__Lachnospiraceae; g__Lachnospira; s__ |
| Seq322645 | 17.93848 | 0.000127 | 0.015481 | 1 | 9.290323 | 0.351852 | 3.283019 | k__Bacteria; p__Firmicutes; c__Clostridia; o__Clostridiales; f__Ruminococcaceae; g__; s__ |
| Seq328738 | 17.91699 | 0.000129 | 0.015573 | 1 | 1.774194 | 33.24074 | 27.41509 | k__Bacteria; p__Proteobacteria; c__Gammaproteobacteria; o__Enterobacteriales; f__Enterobacteriaceae |
| Seq295359 | 17.86002 | 0.000132 | 0.015947 | 1 | 50.16129 | 11.92593 | 22.26415 | k__Bacteria; p__Firmicutes; c__Clostridia; o__Clostridiales; f__Ruminococcaceae; g__Faecalibacterium; s__prausnitzii |
| Seq293002 | 17.8456 | 0.000133 | 0.015978 | 1 | 2.483871 | 32.57407 | 26.4717 | k__Bacteria; p__Proteobacteria; c__Gammaproteobacteria; o__Enterobacteriales; f__Enterobacteriaceae; g__Klebsiella; s__ |
| Seq320209 | 17.83709 | 0.000134 | 0.015978 | 1 | 4.709677 | 0.407407 | 0.735849 | k__Bacteria; p__Firmicutes; c__Clostridia; o__Clostridiales; f__Lachnospiraceae; g__Dorea; s__ |
| Seq284399 | 17.80333 | 0.000136 | 0.015994 | 1 | 6.032258 | 0.425926 | 2.622642 | k__Bacteria; p__Firmicutes; c__Clostridia; o__Clostridiales; f__Lachnospiraceae; g__; s__ |
| Seq328226 | 17.77091 | 0.000138 | 0.015994 | 1 | 2.580645 | 0 | 0 | k__Bacteria; p__Firmicutes; c__Clostridia; o__Clostridiales; f__Lachnospiraceae; g__; s__ |
| Seq314656 | 17.76975 | 0.000138 | 0.015994 | 1 | 1.387097 | 0 | 0 | k__Bacteria; p__Firmicutes; c__Clostridia; o__Clostridiales; f__Lachnospiraceae; g__Lachnospira; s__ |
| Seq309844 | 17.76936 | 0.000138 | 0.015994 | 1 | 4.741935 | 0 | 0 | k__Bacteria; p__Bacteroidetes; c__Bacteroidia; o__Bacteroidales; f__Bacteroidaceae; g__Bacteroides; s__plebeius |
| Seq285606 | 17.76936 | 0.000138 | 0.015994 | 1 | 5.451613 | 0 | 0 | k__Bacteria; p__Firmicutes; c__Clostridia; o__Clostridiales; f__Lachnospiraceae; g__; s__ |
| Seq295108 | 17.76936 | 0.000138 | 0.015994 | 1 | 3.774194 | 0 | 0 | k__Bacteria; p__Firmicutes; c__Clostridia; o__Clostridiales; f__Ruminococcaceae; g__Oscillospira; s__ |
| Seq290740 | 17.76936 | 0.000138 | 0.015994 | 1 | 4.225806 | 0 | 0 | k__Bacteria; p__Firmicutes; c__Clostridia; o__Clostridiales; f__Lachnospiraceae; g__Lachnospira; s__ |
| Seq328162 | 17.76059 | 0.000139 | 0.015994 | 1 | 10.70968 | 5.666667 | 27.20755 | k__Bacteria; p__Bacteroidetes; c__Bacteroidia; o__Bacteroidales; f__Bacteroidaceae; g__Bacteroides; s__caccae |
| Seq297052 | 17.74927 | 0.00014 | 0.016012 | 1 | 7.290323 | 0.907407 | 0.924528 | k__Bacteria; p__Firmicutes; c__Clostridia; o__Clostridiales; f__Lachnospiraceae; g__Dorea; s__ |
| Seq320707 | 17.69177 | 0.000144 | 0.016404 | 1 | 7.677419 | 0.055556 | 0.45283 | k__Bacteria; p__Firmicutes; c__Clostridia; o__Clostridiales; f__Lachnospiraceae; g__Clostridium; s__colinum |
| Seq325805 | 17.67632 | 0.000145 | 0.016457 | 1 | 7.032258 | 0.537037 | 0.113208 | k__Bacteria; p__Firmicutes; c__Clostridia; o__Clostridiales; f__Lachnospiraceae; g__Clostridium; s__colinum |
| Seq294388 | 17.63625 | 0.000148 | 0.016699 | 1 | 4.354839 | 0.425926 | 2.849057 | k__Bacteria; p__Firmicutes; c__Clostridia; o__Clostridiales; f__Lachnospiraceae; g__; s__ |
| Seq301266 | 17.62919 | 0.000149 | 0.016699 | 1 | 8.032258 | 6.574074 | 29.77358 | k__Bacteria; p__Bacteroidetes; c__Bacteroidia; o__Bacteroidales; f__Bacteroidaceae; g__Bacteroides |
| Seq290303 | 17.58888 | 0.000152 | 0.016963 | 1 | 11.06452 | 2.5 | 3.207547 | k__Bacteria; p__Firmicutes; c__Clostridia; o__Clostridiales; f__Lachnospiraceae; g__; s__ |
| Seq295754 | 17.55012 | 0.000155 | 0.017219 | 1 | 5.870968 | 0.185185 | 0.773585 | k__Bacteria; p__Firmicutes; c__Clostridia; o__Clostridiales; f__Ruminococcaceae; g__Butyricicoccus; s__pullicaecorum |
| Seq291853 | 17.49966 | 0.000158 | 0.017581 | 1 | 10.87097 | 4.407407 | 3.056604 | k__Bacteria; p__Firmicutes; c__Clostridia; o__Clostridiales; f__Lachnospiraceae; g__; s__ |
| Seq308375 | 17.47323 | 0.000161 | 0.017737 | 1 | 9.870968 | 0 | 0.849057 | k__Bacteria; p__Firmicutes; c__Clostridia; o__Clostridiales; f__Lachnospiraceae; g__Clostridium; s__colinum |
| Seq284326 | 17.45084 | 0.000162 | 0.017858 | 1 | 8.258065 | 0.796296 | 0 | k__Bacteria; p__Firmicutes; c__Clostridia; o__Clostridiales; f__Lachnospiraceae; g__Clostridium; s__colinum |
| Seq324697 | 17.41293 | 0.000166 | 0.018089 | 1 | 10 | 8.518519 | 34.58491 | k__Bacteria; p__Bacteroidetes; c__Bacteroidia; o__Bacteroidales; f__Bacteroidaceae; g__Bacteroides; s__ |
| Seq311900 | 17.40778 | 0.000166 | 0.018089 | 1 | 3.225806 | 31.35185 | 27.73585 | k__Bacteria; p__Proteobacteria; c__Gammaproteobacteria; o__Enterobacteriales; f__Enterobacteriaceae |
| Seq285780 | 17.37293 | 0.000169 | 0.018273 | 1 | 20.96774 | 5.444444 | 9.113208 | k__Bacteria; p__Firmicutes; c__Clostridia; o__Clostridiales; f__Lachnospiraceae; g__; s__ |
| Seq319781 | 17.36191 | 0.00017 | 0.018273 | 1 | 4.290323 | 1.5 | 0 | k__Bacteria; p__Firmicutes; c__Clostridia; o__Clostridiales; f__Ruminococcaceae; g__Butyricicoccus; s__pullicaecorum |
| Seq314520 | 17.36169 | 0.00017 | 0.018273 | 1 | 5.064516 | 0.981481 | 0 | k__Bacteria; p__Firmicutes; c__Clostridia; o__Clostridiales; f__Lachnospiraceae; g__Clostridium; s__colinum |
| Seq309093 | 17.31385 | 0.000174 | 0.018636 | 1 | 5.677419 | 0.388889 | 1.207547 | k__Bacteria; p__Firmicutes; c__Clostridia; o__Clostridiales; f__Lachnospiraceae; g__; s__ |
| Seq292719 | 17.29617 | 0.000175 | 0.018722 | 1 | 3.612903 | 0 | 0.490566 | k__Bacteria; p__Firmicutes; c__Clostridia; o__Clostridiales; f__Ruminococcaceae; g__Butyricicoccus; s__pullicaecorum |
| Seq296470 | 17.28572 | 0.000176 | 0.01874 | 1 | 17.3871 | 2.666667 | 5.150943 | k__Bacteria; p__Firmicutes; c__Clostridia; o__Clostridiales; f__Lachnospiraceae; g__Roseburia; s__ |
| Seq315998 | 17.25178 | 0.000179 | 0.018981 | 1 | 9 | 3.666667 | 4.773585 | k__Bacteria; p__Firmicutes; c__Clostridia; o__Clostridiales; f__Lachnospiraceae; g__Roseburia; s__faecis |
| Seq313929 | 17.21934 | 0.000182 | 0.019211 | 1 | 17.19355 | 7.833333 | 6.735849 | k__Bacteria; p__Firmicutes; c__Clostridia; o__Clostridiales; f__Lachnospiraceae; g__Roseburia; s__faecis |
| Seq319449 | 17.1982 | 0.000184 | 0.019279 | 1 | 11.87097 | 0.796296 | 0.943396 | k__Bacteria; p__Firmicutes; c__Clostridia; o__Clostridiales; f__Lachnospiraceae; g__Clostridium; s__colinum |
| Seq301429 | 17.19553 | 0.000185 | 0.019279 | 1 | 10.58065 | 3.12963 | 4.09434 | k__Bacteria; p__Firmicutes; c__Clostridia; o__Clostridiales; f__Lachnospiraceae; g__Roseburia; s__ |
| Seq315640 | 17.16504 | 0.000187 | 0.019495 | 1 | 14.03226 | 9.740741 | 5.584906 | k__Bacteria; p__Firmicutes; c__Clostridia; o__Clostridiales; f__Lachnospiraceae; g__Roseburia; s__faecis |
| Seq327345 | 17.11859 | 0.000192 | 0.019871 | 1 | 35.48387 | 8.703704 | 16.54717 | k__Bacteria; p__Firmicutes; c__Clostridia; o__Clostridiales; f__Ruminococcaceae; g__Faecalibacterium; s__prausnitzii |
| Seq283884 | 17.08194 | 0.000195 | 0.020155 | 1 | 15.41935 | 4.851852 | 7.169811 | k__Bacteria; p__Bacteroidetes; c__Bacteroidia; o__Bacteroidales; f__Bacteroidaceae; g__Bacteroides; s__ |
| Seq294111 | 17.06048 | 0.000197 | 0.02029 | 1 | 6.516129 | 0.092593 | 0.45283 | k__Bacteria; p__Firmicutes; c__Clostridia; o__Clostridiales; f__Lachnospiraceae; g__; s__ |
| Seq287220 | 17.03593 | 0.0002 | 0.020303 | 1 | 6.548387 | 1.907407 | 1.226415 | k__Bacteria; p__Firmicutes; c__Clostridia; o__Clostridiales; f__Lachnospiraceae; g__Lachnospira; s__ |
| Seq305258 | 17.03108 | 0.0002 | 0.020303 | 1 | 15.74194 | 5.444444 | 6.735849 | k__Bacteria; p__Firmicutes; c__Clostridia; o__Clostridiales; f__Lachnospiraceae; g__Roseburia; s__faecis |
| Seq287402 | 17.00945 | 0.000203 | 0.020303 | 1 | 4.516129 | 1.240741 | 0 | k__Bacteria; p__Firmicutes; c__Clostridia; o__Clostridiales; f__Lachnospiraceae; g__Dorea; s__formicigenerans |
| Seq310381 | 17.00936 | 0.000203 | 0.020303 | 1 | 31.16129 | 7.703704 | 13.30189 | k__Bacteria; p__Firmicutes; c__Clostridia; o__Clostridiales; f__Ruminococcaceae; g__Faecalibacterium; s__prausnitzii |
| Seq298208 | 17.00852 | 0.000203 | 0.020303 | 1 | 9.935484 | 4.388889 | 4.981132 | k__Bacteria; p__Firmicutes; c__Clostridia; o__Clostridiales; f__Lachnospiraceae; g__Roseburia; s__faecis |
| Seq306332 | 16.99632 | 0.000204 | 0.020303 | 1 | 13.58065 | 5.796296 | 5.283019 | k__Bacteria; p__Firmicutes; c__Clostridia; o__Clostridiales; f__Lachnospiraceae; g__Roseburia; s__faecis |
| Seq327194 | 16.98901 | 0.000205 | 0.020303 | 1 | 4.322581 | 0.37037 | 0 | k__Bacteria; p__Firmicutes; c__Clostridia; o__Clostridiales; f__Lachnospiraceae; g__; s__ |
| Seq284718 | 16.98874 | 0.000205 | 0.020303 | 1 | 4.548387 | 0.388889 | 0 | k__Bacteria; p__Firmicutes; c__Clostridia; o__Clostridiales; f__Lachnospiraceae |
| Seq326192 | 16.98666 | 0.000205 | 0.020303 | 1 | 2.967742 | 31.40741 | 29.83019 | k__Bacteria; p__Proteobacteria; c__Gammaproteobacteria; o__Enterobacteriales; f__Enterobacteriaceae |
| Seq297721 | 16.97915 | 0.000206 | 0.020303 | 1 | 12.25806 | 1.722222 | 5.433962 | k__Bacteria; p__Firmicutes; c__Clostridia; o__Clostridiales; f__Lachnospiraceae; g__Roseburia; s__ |
| Seq304818 | 16.95634 | 0.000208 | 0.020395 | 1 | 9.645161 | 2.611111 | 3.415094 | k__Bacteria; p__Firmicutes; c__Clostridia; o__Clostridiales; f__Lachnospiraceae; g__; s__ |
| Seq311397 | 16.9545 | 0.000208 | 0.020395 | 1 | 4.903226 | 2.277778 | 3.358491 | k__Bacteria; p__Firmicutes; c__Clostridia; o__Clostridiales; f__Lachnospiraceae; g__; s__ |
| Seq286668 | 16.93959 | 0.00021 | 0.020468 | 1 | 18.09677 | 7.518519 | 7.415094 | k__Bacteria; p__Firmicutes; c__Clostridia; o__Clostridiales; f__Lachnospiraceae; g__Roseburia; s__faecis |
| Seq291469 | 16.92704 | 0.000211 | 0.020517 | 1 | 4.516129 | 1.166667 | 1.226415 | k__Bacteria; p__Firmicutes; c__Clostridia; o__Clostridiales; f__Lachnospiraceae; g__Dorea; s__ |
| Seq293401 | 16.90918 | 0.000213 | 0.020616 | 1 | 3.741935 | 3.981481 | 0 | k__Bacteria; p__Firmicutes; c__Clostridia; o__Clostridiales; f__Lachnospiraceae; g__Lachnospira; s__ |
| Seq300268 | 16.89538 | 0.000214 | 0.020616 | 1 | 4.548387 | 2.296296 | 0.188679 | k__Bacteria; p__Firmicutes; c__Clostridia; o__Clostridiales; f__Lachnospiraceae; g__Dorea; s__ |
| Seq313522 | 16.88651 | 0.000215 | 0.020616 | 1 | 1.419355 | 24.64815 | 20.98113 | k__Bacteria; p__Proteobacteria; c__Gammaproteobacteria; o__Enterobacteriales; f__Enterobacteriaceae |
| Seq286269 | 16.88446 | 0.000216 | 0.020616 | 1 | 4.709677 | 0.5 | 0 | k__Bacteria; p__Firmicutes; c__Clostridia; o__Clostridiales; f__Lachnospiraceae; g__; s__ |
| Seq287097 | 16.87919 | 0.000216 | 0.020616 | 1 | 39.80645 | 9.888889 | 13.98113 | k__Bacteria; p__Firmicutes; c__Clostridia; o__Clostridiales; f__Ruminococcaceae; g__Faecalibacterium; s__prausnitzii |
| Seq295265 | 16.86505 | 0.000218 | 0.020684 | 1 | 8.387097 | 0.074074 | 1.075472 | k__Bacteria; p__Firmicutes; c__Clostridia; o__Clostridiales; f__Lachnospiraceae; g__Clostridium; s__colinum |
| Seq314304 | 16.83412 | 0.000221 | 0.020927 | 1 | 7.290323 | 0.888889 | 0.90566 | k__Bacteria; p__Firmicutes; c__Clostridia; o__Clostridiales; f__Lachnospiraceae; g__; s__ |
| Seq290702 | 16.78088 | 0.000227 | 0.021411 | 1 | 2.483871 | 0.592593 | 0 | k__Bacteria; p__Firmicutes; c__Clostridia; o__Clostridiales; f__Lachnospiraceae; g__Dorea; s__formicigenerans |
| Seq320336 | 16.76287 | 0.000229 | 0.021516 | 1 | 11.3871 | 1.481481 | 0 | k__Bacteria; p__Firmicutes; c__Clostridia; o__Clostridiales; f__Lachnospiraceae; g__Clostridium; s__colinum |
| Seq284736 | 16.75621 | 0.00023 | 0.021516 | 1 | 37.35484 | 9.592593 | 14.49057 | k__Bacteria; p__Firmicutes; c__Clostridia; o__Clostridiales; f__Ruminococcaceae; g__Faecalibacterium; s__prausnitzii |
| Seq295061 | 16.68504 | 0.000238 | 0.022213 | 1 | 1.612903 | 0 | 0.981132 | k__Bacteria; p__Firmicutes; c__Clostridia; o__Clostridiales; f__Lachnospiraceae; g__Lachnospira; s__ |
| Seq324769 | 16.66988 | 0.00024 | 0.022299 | 1 | 11.16129 | 0.351852 | 0.641509 | k__Bacteria; p__Firmicutes; c__Clostridia; o__Clostridiales; f__Lachnospiraceae; g__Clostridium; s__colinum |
| Seq298398 | 16.61754 | 0.000246 | 0.022781 | 1 | 5.129032 | 0.407407 | 1.226415 | k__Bacteria; p__Firmicutes; c__Clostridia; o__Clostridiales; f__Lachnospiraceae; g__; s__ |
| Seq321740 | 16.61239 | 0.000247 | 0.022781 | 1 | 48.6129 | 13.27778 | 16.66038 | k__Bacteria; p__Firmicutes; c__Clostridia; o__Clostridiales; f__Ruminococcaceae; g__Faecalibacterium; s__prausnitzii |
| Seq328086 | 16.57816 | 0.000251 | 0.02309 | 1 | 10.93548 | 7.907407 | 33 | k__Bacteria; p__Bacteroidetes; c__Bacteroidia; o__Bacteroidales; f__Bacteroidaceae; g__Bacteroides; s__caccae |
| Seq320881 | 16.47422 | 0.000265 | 0.024233 | 1 | 5.806452 | 0.5 | 0.509434 | k__Bacteria; p__Firmicutes; c__Clostridia; o__Clostridiales; f__Lachnospiraceae; g__; s__ |
| Seq318721 | 16.40476 | 0.000274 | 0.024999 | 1 | 18.22581 | 4.555556 | 5.528302 | k__Bacteria; p__Firmicutes; c__Clostridia; o__Clostridiales; f__Lachnospiraceae; g__; s__ |
| Seq317806 | 16.3867 | 0.000276 | 0.025086 | 1 | 16.3871 | 14.53704 | 45.09434 | k__Bacteria; p__Bacteroidetes; c__Bacteroidia; o__Bacteroidales; f__Bacteroidaceae; g__Bacteroides |
| Seq327857 | 16.38005 | 0.000277 | 0.025086 | 1 | 14.77419 | 6.444444 | 7.471698 | k__Bacteria; p__Firmicutes; c__Clostridia; o__Clostridiales; f__Lachnospiraceae; g__Roseburia; s__faecis |
| Seq319987 | 16.3762 | 0.000278 | 0.025086 | 1 | 6.032258 | 2.703704 | 2.018868 | k__Bacteria; p__Firmicutes; c__Clostridia; o__Clostridiales; f__Lachnospiraceae; g__; s__ |
| Seq314914 | 16.31998 | 0.000286 | 0.025708 | 1 | 4.903226 | 0.462963 | 1.509434 | k__Bacteria; p__Firmicutes; c__Clostridia; o__Clostridiales; f__Lachnospiraceae; g__; s__ |
| Seq309403 | 16.30356 | 0.000288 | 0.025785 | 1 | 1.870968 | 25.55556 | 22.4717 | k__Bacteria; p__Proteobacteria; c__Gammaproteobacteria; o__Enterobacteriales; f__Enterobacteriaceae |
| Seq285980 | 16.28903 | 0.00029 | 0.025785 | 1 | 19.32258 | 5.407407 | 6.056604 | k__Bacteria; p__Firmicutes; c__Clostridia; o__Clostridiales; f__Lachnospiraceae; g__; s__ |
| Seq320708 | 16.28349 | 0.000291 | 0.025785 | 1 | 5.129032 | 0.12963 | 2.735849 | k__Bacteria; p__Firmicutes; c__Clostridia; o__Clostridiales; f__Lachnospiraceae; g__Dorea; s__ |
| Seq321259 | 16.28059 | 0.000292 | 0.025785 | 1 | 5.967742 | 0.796296 | 0.754717 | k__Bacteria; p__Firmicutes; c__Clostridia; o__Clostridiales; f__Lachnospiraceae; g__; s__ |
| Seq289646 | 16.2786 | 0.000292 | 0.025785 | 1 | 24.54839 | 6.481481 | 11.86792 | k__Bacteria; p__Firmicutes; c__Clostridia; o__Clostridiales; f__Ruminococcaceae; g__Faecalibacterium; s__prausnitzii |
| Seq310548 | 16.26789 | 0.000293 | 0.025833 | 1 | 33.54839 | 7.62963 | 14.64151 | k__Bacteria; p__Firmicutes; c__Clostridia; o__Clostridiales; f__Ruminococcaceae; g__Faecalibacterium; s__prausnitzii |
| Seq294420 | 16.22856 | 0.000299 | 0.026254 | 1 | 6.935484 | 1.814815 | 0 | k__Bacteria; p__Firmicutes; c__Clostridia; o__Clostridiales; f__Lachnospiraceae; g__Dorea; s__ |
| Seq294311 | 16.20032 | 0.000303 | 0.026535 | 1 | 12.06452 | 3.722222 | 4.660377 | k__Bacteria; p__Firmicutes; c__Clostridia; o__Clostridiales; f__Lachnospiraceae; g__; s__ |
| Seq319084 | 16.1851 | 0.000306 | 0.026646 | 1 | 4.064516 | 0.462963 | 0.641509 | k__Bacteria; p__Firmicutes; c__Clostridia; o__Clostridiales; f__Lachnospiraceae; g__; s__ |
| Seq309560 | 16.15565 | 0.00031 | 0.026948 | 1 | 18.19355 | 4.074074 | 4.54717 | k__Bacteria; p__Firmicutes; c__Clostridia; o__Clostridiales; f__Lachnospiraceae; g__Roseburia; s__ |
| Seq316958 | 16.10715 | 0.000318 | 0.027514 | 1 | 18.70968 | 3.277778 | 8.433962 | k__Bacteria; p__Firmicutes; c__Clostridia; o__Clostridiales; f__Lachnospiraceae; g__Roseburia; s__ |
| Seq329163 | 16.09752 | 0.000319 | 0.027552 | 1 | 7.580645 | 3.222222 | 3.169811 | k__Bacteria; p__Firmicutes; c__Clostridia; o__Clostridiales; f__Lachnospiraceae; g__; s__ |
| Seq286852 | 16.07084 | 0.000324 | 0.027827 | 1 | 9.677419 | 3.888889 | 4.415094 | k__Bacteria; p__Firmicutes; c__Clostridia; o__Clostridiales; f__Lachnospiraceae; g__; s__ |
| Seq324805 | 16.03662 | 0.000329 | 0.028175 | 1 | 5.870968 | 1.666667 | 1.377358 | k__Bacteria; p__Firmicutes; c__Clostridia; o__Clostridiales; f__Lachnospiraceae; g__Lachnospira; s__ |
| Seq299357 | 16.03239 | 0.00033 | 0.028175 | 1 | 4.419355 | 1.759259 | 3.207547 | k__Bacteria; p__Firmicutes; c__Clostridia; o__Clostridiales; f__Lachnospiraceae; g__Lachnospira; s__ |
| Seq301278 | 16.02481 | 0.000331 | 0.028186 | 1 | 36.74194 | 10.38889 | 16.09434 | k__Bacteria; p__Firmicutes; c__Clostridia; o__Clostridiales; f__Ruminococcaceae; g__Faecalibacterium; s__prausnitzii |
| Seq291218 | 15.96003 | 0.000342 | 0.028982 | 1 | 5.258065 | 2.481481 | 1.113208 | k__Bacteria; p__Firmicutes; c__Clostridia; o__Clostridiales; f__Lachnospiraceae; g__Lachnospira; s__ |
| Seq325066 | 15.95165 | 0.000344 | 0.028982 | 1 | 12.90323 | 5.166667 | 6.509434 | k__Bacteria; p__Firmicutes; c__Clostridia; o__Clostridiales; f__Lachnospiraceae; g__Roseburia; s__faecis |
| Seq303460 | 15.94893 | 0.000344 | 0.028982 | 1 | 14.45161 | 3.555556 | 4.603774 | k__Bacteria; p__Firmicutes; c__Clostridia; o__Clostridiales; f__Lachnospiraceae; g__Roseburia; s__ |
| Seq316615 | 15.89858 | 0.000353 | 0.029622 | 1 | 4.451613 | 2.703704 | 3.150943 | k__Bacteria; p__Firmicutes; c__Clostridia; o__Clostridiales; f__Lachnospiraceae; g__Lachnospira; s__ |
| Seq301368 | 15.8834 | 0.000356 | 0.029749 | 1 | 9.580645 | 4.314815 | 5.037736 | k__Bacteria; p__Firmicutes; c__Clostridia; o__Clostridiales; f__Lachnospiraceae; g__Roseburia; s__faecis |
| Seq290021 | 15.85212 | 0.000361 | 0.030118 | 1 | 7.935484 | 2.981481 | 1.830189 | k__Bacteria; p__Firmicutes; c__Clostridia; o__Clostridiales; f__Lachnospiraceae; g__; s__ |
| Seq285228 | 15.79181 | 0.000372 | 0.030937 | 1 | 1.419355 | 23.35185 | 19.09434 | k__Bacteria; p__Proteobacteria; c__Gammaproteobacteria; o__Enterobacteriales; f__Enterobacteriaceae |
| Seq288678 | 15.77557 | 0.000375 | 0.031087 | 1 | 5.548387 | 2.592593 | 0.45283 | k__Bacteria; p__Firmicutes; c__Clostridia; o__Clostridiales; f__Lachnospiraceae; g__Lachnospira; s__ |
| Seq323487 | 15.75605 | 0.000379 | 0.031289 | 1 | 11.19355 | 4.351852 | 5.981132 | k__Bacteria; p__Firmicutes; c__Clostridia; o__Clostridiales; f__Lachnospiraceae; g__Roseburia; s__faecis |
| Seq314937 | 15.73365 | 0.000383 | 0.031538 | 1 | 4.612903 | 3.092593 | 0.245283 | k__Bacteria; p__Firmicutes; c__Clostridia; o__Clostridiales; f__Lachnospiraceae; g__Lachnospira; s__ |
| Seq289410 | 15.70702 | 0.000388 | 0.031857 | 1 | 2.935484 | 33.64815 | 27.18868 | k__Bacteria; p__Proteobacteria; c__Gammaproteobacteria; o__Enterobacteriales; f__Enterobacteriaceae |
| Seq306974 | 15.66888 | 0.000396 | 0.032364 | 1 | 16.16129 | 9.722222 | 39.22642 | k__Bacteria; p__Bacteroidetes; c__Bacteroidia; o__Bacteroidales; f__Bacteroidaceae; g__Bacteroides; s__ |
| Seq312179 | 15.64333 | 0.000401 | 0.032675 | 1 | 7.806452 | 1.814815 | 0.433962 | k__Bacteria; p__Firmicutes; c__Clostridia; o__Clostridiales; f__Lachnospiraceae; g__; s__ |
| Seq286689 | 15.60926 | 0.000408 | 0.033129 | 1 | 11.32258 | 6.5 | 3.037736 | k__Bacteria; p__Firmicutes; c__Clostridia; o__Clostridiales; f__Lachnospiraceae; g__Lachnospira; s__ |
| Seq318110 | 15.59405 | 0.000411 | 0.033274 | 1 | 2.096774 | 30.90741 | 24.5283 | k__Bacteria; p__Proteobacteria; c__Gammaproteobacteria; o__Enterobacteriales; f__Enterobacteriaceae |
| Seq318001 | 15.51708 | 0.000427 | 0.034469 | 1 | 12.41935 | 5.37037 | 6.037736 | k__Bacteria; p__Firmicutes; c__Clostridia; o__Clostridiales; f__Lachnospiraceae; g__Roseburia; s__faecis |
| Seq317052 | 15.49693 | 0.000431 | 0.034707 | 1 | 32.74194 | 8.037037 | 18.28302 | k__Bacteria; p__Firmicutes; c__Clostridia; o__Clostridiales; f__Ruminococcaceae; g__Faecalibacterium; s__prausnitzii |
| Seq285343 | 15.40812 | 0.000451 | 0.036167 | 1 | 14.16129 | 8.444444 | 6.867925 | k__Bacteria; p__Firmicutes; c__Clostridia; o__Clostridiales; f__Lachnospiraceae; g__Roseburia; s__faecis |
| Seq323720 | 15.3393 | 0.000467 | 0.037314 | 1 | 37.16129 | 10.96296 | 14.09434 | k__Bacteria; p__Firmicutes; c__Clostridia; o__Clostridiales; f__Ruminococcaceae; g__Faecalibacterium; s__prausnitzii |
| Seq295337 | 15.28088 | 0.000481 | 0.038299 | 1 | 6.096774 | 4.981481 | 1.415094 | k__Bacteria; p__Firmicutes; c__Clostridia; o__Clostridiales; f__Lachnospiraceae; g__Lachnospira; s__ |
| Seq310467 | 15.17516 | 0.000507 | 0.040156 | 1 | 8.16129 | 3.87037 | 2.735849 | k__Bacteria; p__Firmicutes; c__Clostridia; o__Clostridiales; f__Lachnospiraceae; g__; s__ |
| Seq288939 | 15.17357 | 0.000507 | 0.040156 | 1 | 11.70968 | 1.12963 | 1.018868 | k__Bacteria; p__Firmicutes; c__Clostridia; o__Clostridiales; f__Lachnospiraceae; g__Clostridium; s__colinum |
| Seq294108 | 15.09367 | 0.000528 | 0.041661 | 1 | 2.483871 | 31.59259 | 28.90566 | k__Bacteria; p__Proteobacteria; c__Gammaproteobacteria; o__Enterobacteriales; f__Enterobacteriaceae |
| Seq305373 | 15.08247 | 0.000531 | 0.041705 | 1 | 7.903226 | 0.907407 | 0.283019 | k__Bacteria; p__Firmicutes; c__Clostridia; o__Clostridiales; f__Lachnospiraceae; g__Clostridium; s__colinum |
| Seq327892 | 15.07906 | 0.000532 | 0.041705 | 1 | 13.12903 | 9.055556 | 34.92453 | k__Bacteria; p__Bacteroidetes; c__Bacteroidia; o__Bacteroidales; f__Bacteroidaceae; g__Bacteroides; s__caccae |
| Seq285297 | 15.06746 | 0.000535 | 0.041818 | 1 | 12.48387 | 3.62963 | 3.377358 | k__Bacteria; p__Firmicutes; c__Clostridia; o__Clostridiales; f__Lachnospiraceae; g__Roseburia; s__ |
| Seq288422 | 15.04368 | 0.000541 | 0.042043 | 1 | 13.22581 | 5.407407 | 27.98113 | k__Bacteria; p__Bacteroidetes; c__Bacteroidia; o__Bacteroidales; f__Bacteroidaceae; g__Bacteroides |
| Seq312342 | 15.03661 | 0.000543 | 0.042043 | 1 | 26.16129 | 8.055556 | 12.41509 | k__Bacteria; p__Firmicutes; c__Clostridia; o__Clostridiales; f__Ruminococcaceae; g__Faecalibacterium; s__prausnitzii |
| Seq326633 | 15.03581 | 0.000543 | 0.042043 | 1 | 3.419355 | 2.407407 | 0.943396 | k__Bacteria; p__Firmicutes; c__Clostridia; o__Clostridiales; f__Lachnospiraceae; g__Lachnospira; s__ |
| Seq318248 | 15.03201 | 0.000544 | 0.042043 | 1 | 16.45161 | 8.962963 | 4.698113 | k__Bacteria; p__Firmicutes; c__Clostridia; o__Clostridiales; f__Lachnospiraceae; g__Lachnospira; s__ |
| Seq320674 | 14.99969 | 0.000553 | 0.042597 | 1 | 9.387097 | 4.833333 | 4.849057 | k__Bacteria; p__Firmicutes; c__Clostridia; o__Clostridiales; f__Lachnospiraceae; g__Roseburia; s__faecis |
| Seq293807 | 14.97095 | 0.000561 | 0.043082 | 1 | 14.87097 | 6.740741 | 7.528302 | k__Bacteria; p__Firmicutes; c__Clostridia; o__Clostridiales; f__Lachnospiraceae; g__Roseburia; s__faecis |
| Seq306497 | 14.94 | 0.00057 | 0.043537 | 1 | 10.87097 | 1.203704 | 0.603774 | k__Bacteria; p__Firmicutes; c__Clostridia; o__Clostridiales; f__Lachnospiraceae; g__Clostridium; s__colinum |
| Seq328974 | 14.93779 | 0.000571 | 0.043537 | 1 | 15.03226 | 10.07407 | 38.84906 | k__Bacteria; p__Bacteroidetes; c__Bacteroidia; o__Bacteroidales; f__Bacteroidaceae; g__Bacteroides |
| Seq306389 | 14.92887 | 0.000573 | 0.0436 | 1 | 3.967742 | 2.740741 | 0.679245 | k__Bacteria; p__Firmicutes; c__Clostridia; o__Clostridiales; f__Lachnospiraceae; g__Lachnospira; s__ |
| Seq326267 | 14.87195 | 0.00059 | 0.044723 | 1 | 5.645161 | 0.592593 | 3.150943 | k__Bacteria; p__Firmicutes; c__Clostridia; o__Clostridiales; f__Lachnospiraceae; g__Dorea; s__ |
| Seq323726 | 14.85696 | 0.000594 | 0.044924 | 1 | 3.935484 | 0.814815 | 0.245283 | k__Bacteria; p__Firmicutes; c__Clostridia; o__Clostridiales; f__Lachnospiraceae; g__Lachnospira; s__ |
| Seq320489 | 14.83842 | 0.0006 | 0.045207 | 1 | 7.967742 | 0.87037 | 1.301887 | k__Bacteria; p__Firmicutes; c__Clostridia; o__Clostridiales; f__Ruminococcaceae; g__Butyricicoccus; s__pullicaecorum |
| Seq297154 | 14.82935 | 0.000602 | 0.045277 | 1 | 5.935484 | 1.833333 | 2.566038 | k__Bacteria; p__Firmicutes; c__Clostridia; o__Clostridiales; f__Lachnospiraceae; g__Lachnospira; s__ |
| Seq290268 | 14.77656 | 0.000618 | 0.046349 | 1 | 6.741935 | 1.648148 | 1.509434 | k__Bacteria; p__Firmicutes; c__Clostridia; o__Clostridiales; f__Lachnospiraceae; g__Lachnospira; s__ |
| Seq301822 | 14.75925 | 0.000624 | 0.046598 | 1 | 13.06452 | 10.01852 | 36.13208 | k__Bacteria; p__Bacteroidetes; c__Bacteroidia; o__Bacteroidales; f__Bacteroidaceae; g__Bacteroides; s__caccae |
| Seq320083 | 14.75401 | 0.000625 | 0.046598 | 1 | 5.709677 | 2.166667 | 0.245283 | k__Bacteria; p__Firmicutes; c__Clostridia; o__Clostridiales; f__Lachnospiraceae; g__Dorea; s__ |
| Seq324337 | 14.69917 | 0.000643 | 0.047752 | 1 | 4.580645 | 0 | 2.113208 | k__Bacteria; p__Firmicutes; c__Clostridia; o__Clostridiales; f__Ruminococcaceae; g__Oscillospira |
| Seq289171 | 14.67248 | 0.000651 | 0.048251 | 1 | 5.580645 | 0 | 2.716981 | k__Bacteria; p__Firmicutes; c__Clostridia; o__Clostridiales; f__Ruminococcaceae |
| Seq291191 | 14.63841 | 0.000663 | 0.048936 | 1 | 4.419355 | 0.444444 | 0.660377 | k__Bacteria; p__Firmicutes; c__Clostridia; o__Clostridiales; f__Lachnospiraceae; g__Lachnospira; s__ |
| Seq294695 | 14.61465 | 0.000671 | 0.049376 | 1 | 2.193548 | 32.81481 | 24.01887 | k__Bacteria; p__Proteobacteria; c__Gammaproteobacteria; o__Enterobacteriales; f__Enterobacteriaceae |
| Seq294019 | 14.60851 | 0.000673 | 0.049383 | 1 | 7.258065 | 1.555556 | 1.54717 | k__Bacteria; p__Firmicutes; c__Clostridia; o__Clostridiales; f__Lachnospiraceae; g__; s__ |

**Supplementary Table 2. Comparison of relative taxonomic abundance at family and genus level in HC, LBP+FR and LBP cohorts.**

Sample size is n=31 HC, n=54 LBP+FR, n=53 LBP as biologically independent samples. Data are shown as mean % ± standard error of the mean (SEM). Wilcoxon rank-sum Test calculated *P* values for 2 group comparisons. *P* < 0.05 considered statistically significant.

| **Taxonomy (relative abundance, %)** | **HC Mean** | | **LBP+FR** | | **LBP** | | ***P*-value LBP+FR vs LBP** | ***P*-value LBP+FR vs HC** | ***P*-value LBP vs HC** |
| --- | --- | --- | --- | --- | --- | --- | --- | --- | --- |
| **Family level** | **Mean** | **Sem** | **Mean** | **Sem** | **Mean** | **Sem** |  |  |  |
| f__Erysipelotrichaceae | 8.95E-03 | 1.33E-02 | 1.18E-02 | 1.31E-02 | 1.03E-02 | 1.78E-02 | 1.12E-01 | 1.00E-01 | 8.53E-01 |
| f__Streptococcaceae | 4.61E-03 | 1.03E-02 | 1.43E-02 | 2.27E-02 | 1.42E-02 | 2.27E-02 | 8.91E-01 | 3.96E-04 | 2.41E-04 |
| f__Bifidobacteriaceae | 1.38E-02 | 2.40E-02 | 1.09E-02 | 2.42E-02 | 1.46E-02 | 3.00E-02 | 9.33E-01 | 6.58E-01 | 7.38E-01 |
| f__Porphyromonadaceae | 1.88E-02 | 2.48E-02 | 2.37E-02 | 2.37E-02 | 2.46E-02 | 2.19E-02 | 7.67E-01 | 1.18E-01 | 6.44E-02 |
| f__Prevotellaceae | 4.69E-02 | 1.12E-01 | 8.92E-02 | 1.46E-01 | 8.48E-02 | 1.50E-01 | 8.25E-01 | 1.95E-04 | 3.52E-04 |
| f__Rikenellaceae | 5.74E-03 | 9.49E-03 | 1.49E-02 | 2.02E-02 | 1.68E-02 | 1.92E-02 | 4.40E-01 | 1.13E-03 | 1.34E-04 |
| f__Alcaligenaceae | 2.19E-02 | 1.60E-02 | 1.57E-02 | 1.69E-02 | 1.63E-02 | 1.61E-02 | 6.45E-01 | 4.91E-02 | 8.89E-02 |
| f__Clostridiaceae | 1.02E-02 | 1.16E-02 | 2.55E-02 | 3.90E-02 | 2.12E-02 | 2.54E-02 | 6.81E-01 | 3.10E-03 | 7.08E-03 |
| f__Veillonellaceae | 4.37E-02 | 3.66E-02 | 6.94E-02 | 9.69E-02 | 6.54E-02 | 6.65E-02 | 3.48E-01 | 6.98E-01 | 2.52E-01 |
| f__Enterobacteriaceae | 1.18E-02 | 1.55E-02 | 1.13E-01 | 1.24E-01 | 9.42E-02 | 1.07E-01 | 5.13E-01 | 3.26E-10 | 1.79E-09 |
| f__Ruminococcaceae | 2.20E-01 | 1.14E-01 | 9.87E-02 | 5.50E-02 | 1.17E-01 | 8.37E-02 | 5.03E-01 | 2.55E-07 | 2.57E-05 |
| f__Lachnospiraceae | 2.98E-01 | 1.38E-01 | 1.90E-01 | 1.06E-01 | 1.63E-01 | 7.48E-02 | 3.14E-01 | 1.99E-04 | 2.85E-06 |
| f__Bacteroidaceae | 2.51E-01 | 1.54E-01 | 2.39E-01 | 1.71E-01 | 2.68E-01 | 1.62E-01 | 3.23E-01 | 6.98E-01 | 6.46E-01 |
| **Genus level** |  |  |  |  |  |  |  |  |  |
| g_Bacteroides | 2.51E-01 | 1.54E-01 | 2.39E-01 | 1.71E-01 | 2.68E-01 | 1.62E-01 | 3.23E-01 | 6.98E-01 | 6.46E-01 |
| g_Prevotella | 4.69E-02 | 1.12E-01 | 8.91E-02 | 1.46E-01 | 8.47E-02 | 1.50E-01 | 8.30E-01 | 1.95E-04 | 3.52E-04 |
| g_Faecalibacterium | 1.37E-01 | 6.23E-02 | 5.28E-02 | 3.26E-02 | 6.49E-02 | 4.90E-02 | 4.22E-01 | 5.87E-09 | 1.65E-06 |
| g_Escherichia | 7.75E-03 | 1.18E-02 | 7.11E-02 | 1.00E-01 | 5.98E-02 | 7.25E-02 | 6.88E-01 | <0.0001 | <0.0001 |
| g_Roseburia | 6.08E-02 | 4.78E-02 | 2.76E-02 | 2.65E-02 | 3.45E-02 | 3.88E-02 | 8.15E-01 | 2.71E-03 | 1.30E-02 |
| g_Blautia | 2.78E-02 | 2.47E-02 | 3.89E-02 | 3.85E-02 | 3.27E-02 | 4.73E-02 | 1.68E-01 | 1.38E-01 | 7.32E-01 |
| g_Ruminococcus | 4.30E-02 | 5.19E-06 | 2.10E-02 | 2.78E-05 | 3.17E-02 | 4.00E-06 | 1.16E-01 | 9.04E-01 | 6.01E-01 |
| g_[Ruminococcus] | 3.07E-02 | 2.78E-02 | 3.31E-02 | 4.49E-02 | 2.23E-02 | 2.08E-02 | 3.34E-01 | 9.67E-01 | 3.76E-01 |
| g_Enterobacteriaceae | 1.99E-04 | 7.02E-04 | 1.10E-04 | 4.30E-04 | 2.91E-04 | 1.00E-03 | 9.38E-01 | 7.73E-01 | 7.05E-01 |
| g_Parabacteroides | 1.88E-02 | 2.48E-02 | 2.36E-02 | 2.36E-02 | 2.46E-02 | 2.18E-02 | 7.67E-01 | 1.28E-01 | 6.37E-02 |
| g_Megamonas | 1.58E-02 | 3.30E-02 | 2.08E-02 | 4.89E-02 | 2.75E-02 | 5.80E-02 | 8.25E-01 | 3.46E-05 | 4.77E-09 |
| g_Lachnospira | 3.42E-02 | 3.15E-02 | 2.17E-02 | 3.36E-02 | 1.27E-02 | 1.43E-02 | 5.92E-01 | 1.28E-02 | 4.06E-04 |
| g_Sutterella | 2.18E-02 | 1.58E-02 | 1.57E-02 | 1.69E-02 | 1.62E-02 | 1.61E-02 | 7.34E-01 | 4.81E-02 | 8.38E-02 |
| g_Phascolarctobacterium | 1.06E-02 | 1.50E-02 | 1.71E-02 | 2.70E-02 | 1.55E-02 | 2.02E-02 | 7.70E-01 | 2.48E-01 | 1.56E-01 |
| g_Coprococcus | 1.47E-02 | 1.88E-02 | 1.46E-02 | 1.32E-02 | 1.46E-02 | 1.49E-02 | 8.13E-01 | 9.94E-02 | 1.42E-01 |
| g_SMB53 | 6.62E-03 | 8.81E-03 | 1.57E-02 | 2.13E-02 | 1.71E-02 | 2.43E-02 | 5.86E-01 | 4.92E-03 | 9.07E-03 |

**Supplementary Table 3. Comparison of underlying disease-correlated KEGG Orthologies (KOs) between LBP+FR, LBP and HC groups.**

|  | **p-value** |
| --- | --- |
| ko00053: Ascorbate and aldarate metabolism | 0.00246576 |
| ko00130: Ubiquinone and other terpenoid-quinone biosynthesis | 0.003575559 |
| ko01053: Biosynthesis of siderophore group nonribosomal peptides | 0.00388629 |
| ko00280: Valine, leucine and isoleucine degradation | 0.004333233 |
| ko00440: Phosphonate and phosphinate metabolism | 0.006677517 |
| ko03410: Base excision repair | 0.006793821 |
| ko00562: Inositol phosphate metabolism | 0.007389777 |
| ko05146: Amoebiasis | 0.008287491 |
| ko00627: Aminobenzoate degradation | 0.009720753 |
| ko03070: Bacterial secretion system | 0.010282341 |
| ko03430: Mismatch repair | 0.012293942 |
| ko04146: Peroxisome | 0.013123729 |
| ko00860: Porphyrin and chlorophyll metabolism | 0.013473142 |
| ko03013: RNA transport | 0.013473142 |
| ko00640: Propanoate metabolism | 0.014377367 |
| ko00660: C5-Branched dibasic acid metabolism | 0.015079117 |
| ko02010: ABC transporters | 0.016800673 |
| ko00230: Purine metabolism | 0.017299857 |
| ko00540: Lipopolysaccharide biosynthesis | 0.018029143 |
| ko00480: Glutathione metabolism | 0.020345513 |
| ko00071: Fatty acid metabolism | 0.020360793 |
| ko00780: Biotin metabolism | 0.021107896 |
| ko03018: RNA degradation | 0.021346941 |
| ko00550: Peptidoglycan biosynthesis | 0.021523959 |
| ko00052: Galactose metabolism | 0.022668323 |
| ko00600: Sphingolipid metabolism | 0.02439386 |
| ko00471: D-Glutamine and D-glutamate metabolism | 0.024749526 |
| ko00473: D-Alanine metabolism | 0.025427553 |
| ko00770: Pantothenate and CoA biosynthesis | 0.02588986 |
| ko03060: Protein export | 0.026006744 |
| ko00010: Glycolysis / Gluconeogenesis | 0.026839842 |
| ko00564: Glycerophospholipid metabolism | 0.027575134 |
| ko00380: Tryptophan metabolism | 0.027619985 |
| ko00362: Benzoate degradation | 0.028005691 |
| ko00920: Sulfur metabolism | 0.028802379 |
| ko00051: Fructose and mannose metabolism | 0.030792612 |
| ko00900: Terpenoid backbone biosynthesis | 0.031234994 |
| ko00633: Nitrotoluene degradation | 0.031850675 |
| ko00910: Nitrogen metabolism | 0.032308257 |
| ko00270: Cysteine and methionine metabolism | 0.034540746 |
| ko00740: Riboflavin metabolism | 0.035969818 |
| ko00360: Phenylalanine metabolism | 0.036872093 |
| ko00520: Amino sugar and nucleotide sugar metabolism | 0.037655386 |
| ko00400: Phenylalanine, tyrosine and tryptophan biosynthesis | 0.037996156 |
| ko03050: Proteasome | 0.038074939 |
| ko02020: Two-component system | 0.039538497 |
| ko05111: Vibrio cholerae pathogenic cycle | 0.040505777 |
| ko03030: DNA replication | 0.042334109 |
| ko00785: Lipoic acid metabolism | 0.042685162 |
| ko05322: Systemic lupus erythematosus | 0.045569796 |
| ko00260: Glycine, serine and threonine metabolism | 0.046290301 |
| ko00450: Selenocompound metabolism | 0.046290301 |
| ko00790: Folate biosynthesis | 0.046290301 |
| ko00630: Glyoxylate and dicarboxylate metabolism | 0.046849694 |
| ko03440: Homologous recombination | 0.046849694 |
| ko00240: Pyrimidine metabolism | 0.048423126 |
| ko00500: Starch and sucrose metabolism | 0.048423126 |
| ko00040: Pentose and glucuronate interconversions | 0.049787068 |

**Supplementary Table 4. The top 50 differential fecal metabolites and enriched pathways in serum samples from the LBP+FR group.**

**TOP50 metabolites in MCs**

| **Family** | **Description** | **KEGG.ID** | **label** | **t.test_p.value** | **wilcox.test_p.value** | **t.test_p.value_BHcorrect** | **wilcox.test_p.value_BHcorrect** | **VIP** |
| --- | --- | --- | --- | --- | --- | --- | --- | --- |
| Terpenoids | Ginsenoside La | null | up | 3.95E-08 | 5.49E-09 | 4.26E-06 | 1.28E-06 | 3.6989 |
| Amino acids, peptides, and analogues | Diphthamide | null | up | 4.45E-08 | 2.94E-08 | 4.51E-06 | 2.76E-06 | 3.5977 |
| Glycerophospholipids[GP] | CDP-DG | null | up | 4.54E-07 | 2.83E-09 | 1.56E-05 | 9.80E-07 | 3.52 |
| 1-benzopyrans | Asticolorin B | null | up | 0.00424188 | 5.72E-05 | 0.014721903 | 0.00039216 | 3.4879 |
| Terpenoids | Ginsenoside C | null | up | 6.95E-07 | 1.95E-06 | 2.00E-05 | 3.37E-05 | 3.4589 |
| Polyketides [PK] | Erythromycin C | null | up | 1.17E-05 | 2.92E-07 | 0.000139861 | 1.03E-05 | 3.4564 |
| Glycerophospholipids[GP] | CDP-DG | null | up | 9.45E-08 | 3.59E-08 | 6.41E-06 | 3.08E-06 | 3.4042 |
| Saccharolipids[SL] | alpha'-Trehalose 6-palmitate | C04265 | up | 1.13E-08 | 9.77E-11 | 2.41E-06 | 3.69E-07 | 3.3986 |
| Sphingolipids [SP] | Fumonisin C3 | null | up | 1.73E-08 | 7.33E-09 | 2.70E-06 | 1.41E-06 | 3.3443 |
| Polyketides [PK] | Nevadensin 5-gentiobioside | null | up | 3.56E-09 | 2.28E-10 | 1.58E-06 | 3.69E-07 | 3.33 |
| Flavonoids | Hordatine B | null | up | 3.86E-05 | 3.72E-06 | 0.000335294 | 5.14E-05 | 3.2624 |
| Terpenoids | Cephalomannine; Taxol B; | C10579 | up | 8.33E-06 | 9.98E-07 | 0.000108493 | 2.18E-05 | 3.2144 |
| Organic acids | Dinophysistoxin 2 | null | up | 0.00026405 | 4.36E-05 | 0.001535963 | 0.00031292 | 3.1717 |
| Glycerophospholipids [GP] | PIM1 | null | up | 2.73E-06 | 6.50E-08 | 5.13E-05 | 4.18E-06 | 3.1505 |
| Organic acids | o-O-sulfate rosiglitazone | null | up | 3.94E-07 | 2.70E-06 | 1.42E-05 | 4.15E-05 | 3.141 |
| Terpenoids | Furohyperforin | null | up | 3.23E-08 | 3.36E-08 | 3.86E-06 | 2.99E-06 | 3.1345 |
| Sterol Lipids [ST] | Spongioside A | null | up | 1.75E-08 | 2.57E-08 | 2.70E-06 | 2.58E-06 | 3.1111 |
| Prenol Lipids [PR] | phorbol 13-acetate 12-myristate | C05151 | up | 6.12E-05 | 1.47E-05 | 0.000482784 | 0.0001382 | 3.0653 |
| Glycerophospholipids[GP] | PS(MonoMe(9,5)/DiMe(13,5)) | null | up | 4.32E-08 | 6.09E-08 | 4.42E-06 | 3.99E-06 | 3.0485 |
| Flavonoids | Viniferal | null | up | 1.12E-07 | 8.41E-08 | 7.02E-06 | 4.74E-06 | 3.0294 |
| Organic acids | Fumonisin B4 | null | up | 3.99E-07 | 5.61E-07 | 1.43E-05 | 1.51E-05 | 3.0263 |
| Polyketides[PK] | 1,26-Dicaffeoylhexacosanediol | null | up | 5.35E-09 | 6.35E-09 | 1.58E-06 | 1.39E-06 | 3.0254 |
| Carbohydrates | N,O-Didesmethylvenlafaxine glucuronide | null | down | 0.00053285 | 0.000287107 | 0.002713476 | 0.00145467 | 2.991 |
| Polyketides [PK] | Petunidin 3-glucoside-5-(6''-acetylglucoside) | null | up | 1.03E-05 | 2.61E-05 | 0.000126211 | 0.00021114 | 2.9868 |
| Glycerophospholipids[GP] | PGP(16:0/16:0) | null | up | 2.75E-06 | 2.49E-05 | 5.15E-05 | 0.00020302 | 2.9518 |
| Amino acids, peptides, and analogues | Cephapirin | C06896 | up | 6.59E-08 | 1.14E-09 | 5.43E-06 | 6.33E-07 | 2.9417 |
| Triazoles | Hexyl heptanoate | null | up | 2.42E-08 | 9.08E-09 | 3.18E-06 | 1.57E-06 | 2.8942 |
| Polyketides [PK] | 6-Hydroxycyanidin 3-glucoside | null | up | 3.14E-07 | 2.10E-09 | 1.22E-05 | 8.73E-07 | 2.8927 |
| Beta lactams | Cefotaxime | C06885 | up | 1.58E-06 | 1.18E-06 | 3.52E-05 | 2.41E-05 | 2.8917 |
| Fatty Acyls [FA] | 6-bromo-tricosa-5E,9Z-dienoic acid | null | down | 0.00368045 | 0.000216187 | 0.013021051 | 0.00115234 | 2.8763 |
| Fatty Acyls [FA] | 3-O-alpha-L-rhamnopyranosyl-3-hydroxydecanoyl-3-hydroxydecanoic acid | null | up | 1.85E-05 | 6.25E-05 | 0.000193598 | 0.00042141 | 2.8741 |
| Fatty Acyls [FA] | Isoamyl 2-furonpropionate | null | up | 0.0002278 | 1.70E-05 | 0.001363414 | 0.00015291 | 2.8586 |
| Amino acids, peptides, and analogues | Oxytocin | null | up | 5.94E-09 | 2.57E-08 | 1.66E-06 | 2.58E-06 | 2.8545 |
| Sphingolipids [SP] | Fumonisin C4 | null | up | 1.17E-07 | 1.49E-07 | 7.07E-06 | 6.78E-06 | 2.851 |
| Terpenoids | Vinaginsenoside R2 | null | up | 1.24E-05 | 2.42E-06 | 0.000144042 | 3.88E-05 | 2.8468 |
| Fatty Acyls [FA] | Butyl 3-O-beta-D-glucopyranosyl-butanoate | null | up | 3.30E-07 | 2.29E-06 | 1.26E-05 | 3.73E-05 | 2.8443 |
| Polyketides [PK] | 2''-O-Vanilloylvitexin | null | up | 1.14E-07 | 2.29E-07 | 7.02E-06 | 8.90E-06 | 2.8365 |
| Fungal toxins | Luteoskyrin; | C16763 | up | 1.30E-07 | 2.92E-07 | 7.50E-06 | 1.03E-05 | 2.8128 |
| Carbohydrates | N-Acetyl-D-glucosaminyldiphosphodolichol | null | up | 0.00012627 | 2.70E-06 | 0.00084944 | 4.15E-05 | 2.8089 |
| Carbohydrates | CMP-N-trimethyl-2-aminoethylphosphonate | C05674 | up | 1.60E-05 | 5.09E-06 | 0.000174636 | 6.37E-05 | 2.805 |
| Amino acids, peptides, and analogues | Mucronine D | null | up | 8.80E-08 | 1.90E-07 | 6.22E-06 | 7.93E-06 | 2.7991 |
| Steroids and derivatives | 4,7-Didehydroneophysalin B | null | up | 2.59E-07 | 3.49E-07 | 1.10E-05 | 1.16E-05 | 2.7953 |
| Organic acids | Fumonisin B2 | null | up | 2.57E-05 | 1.04E-05 | 0.000245917 | 0.00010726 | 2.7699 |
| Alkyl fluorides | Perfluorohexane sulfonic acid | null | up | 1.31E-06 | 9.98E-07 | 3.07E-05 | 2.18E-05 | 2.7547 |
| Fatty Acyls [FA] | Dipropyl hexanedioate | null | down | 0.00272109 | 0.002041516 | 0.010198509 | 0.00727911 | 2.7449 |
| Steroids and derivatives | Corchorusoside E | null | up | 3.81E-05 | 3.72E-06 | 0.000332367 | 5.14E-05 | 2.687 |
| Fatty Acyls [FA] | PGF2alpha-11-acetate methyl ester | null | up | 1.29E-08 | 6.93E-08 | 2.47E-06 | 4.40E-06 | 2.6863 |
| Sphingolipids [SP] | KDNalpha2-3Galbeta1-4Glcbeta-Cer(d18:1/18:0) | null | up | 4.36E-09 | 3.54E-09 | 1.58E-06 | 9.87E-07 | 2.6537 |
| Amino acids, peptides, and analogues | Phenylalanyl-Isoleucine(negrep); | null | up | 3.80E-06 | 1.84E-06 | 6.41E-05 | 3.26E-05 | 2.6536 |
| Fatty acyls[FA] | Heneicosanoyl-CoA | null | up | 3.83E-08 | 5.61E-07 | 4.19E-06 | 1.51E-05 | 2.6378 |

**Enrich pathways in MCs**

| **Pathway** | **Count** | **Metabolites** | **Count.All** | **Pvalue** | **Pathway.ID** |
| --- | --- | --- | --- | --- | --- |
| Valine, leucine and isoleucine degradation | 3 | Thiamine pyrophosphate, 2-Methyl-1-hydroxypropyl-ThPP, 3-Methyl-1-hydroxybutyl-ThPP | 42 | 0.01563138 | map00280 |
| Nicotinate and nicotinamide metabolism | 3 | Nicotinate D-ribonucleoside, Nicotinamide ribotide(negrep), 2,6-Dihydroxypseudooxynicotine, 1-(2,6-Dihydroxypyridin-3-yl)-4-(methylamino) butan-1-one | 55 | 0.03174616 | map00760 |
| Sphingolipid metabolism | 2 | Galactosylsphingosine, Sphinganine 1-phosphate(negrep) | 25 | 0.0389303 | map00600 |
| Amino sugar and nucleotide sugar metabolism | 6 | CDP-4-dehydro-3,6-dideoxy-D-glucose epimer, UDP-4-keto-rhamnose, UDP-2,3-diacetamido-2,3-dideoxy-alpha-D-mannuronate, UDP-alpha-D-ManNAc3NAcA, UDP-2,3-diacetamido-2,3-dideoxy-alpha-D-mannuronic acid, N,N'-Diacetyllegionaminate, UDP-L-Ara4FN, Uridine 5'-diphospho-beta-(4-deoxy-4-formamido-L-arabinose), UDP-4-deoxy-4-formamido-beta-L-arabinopyranose, Cytidine monophosphate N-acetylneuraminic acid | 108 | 0.02355526 | map00520 |
| Neomycin, kanamycin and gentamicin biosynthesis | 5 | Gentamicin C1a, 5''-Phosphoribostamycin, 2'-Dehydrokanamycin A, 2'-Oxokanamycin, Nebramine, 3'-Deoxyneamine, Kanamycin C | 81 | 0.02517913 | map00524 |
| Porphyrin and chlorophyll metabolism | 7 | Cobalt-precorrin 7, Magnesium protoporphyrin, Magnesium protoporphyrin IX, Mg-protoporphyrin IX, L-Urobilinogen(posrep), Chlorophyll a, Sirohydrochlorin, Uroporphyrin I, (3Z)-Phytochromobilin | 142 | 0.02690531 | map00860 |
| Drug metabolism - cytochrome P450 | 5 | 2-Hydroxyfelbamate, Glycinexylidide, Citalopram N-oxide, Atropaldehyde(posrep)(posrep), 4-Glutathionyl cyclophosphamide, Atropaldehyde(posrep)(posrep)(posrep)(posrep)(posrep) | 87 | 0.03293485 | map00982 |

**Supplementary Table 5. RNA sequencing Detailed results of GO and KEGG enrichment analysis.**

**GO analysis (Top50)**

| ID | Description | p.adjust |
| --- | --- | --- |
| GO:0045444 | fat cell differentiation | 0.00078153 |
| GO:0032874 | positive regulation of stress-activated MAPK cascade | 0.00078153 |
| GO:0070304 | positive regulation of stress-activated protein kinase signaling cascade | 0.00078153 |
| GO:0051412 | response to corticosterone | 0.00078153 |
| GO:0045598 | regulation of fat cell differentiation | 0.00078153 |
| GO:0022407 | regulation of cell-cell adhesion | 0.00078153 |
| GO:1903039 | positive regulation of leukocyte cell-cell adhesion | 0.00150119 |
| GO:0022409 | positive regulation of cell-cell adhesion | 0.00150119 |
| GO:0035630 | bone mineralization involved in bone maturation | 0.00158143 |
| GO:0071222 | cellular response to lipopolysaccharide | 0.00211332 |
| GO:0030217 | T cell differentiation | 0.00219543 |
| GO:0050804 | modulation of chemical synaptic transmission | 0.00254174 |
| GO:0099177 | regulation of trans-synaptic signaling | 0.00254174 |
| GO:0071219 | cellular response to molecule of bacterial origin | 0.0027986 |
| GO:0051385 | response to mineralocorticoid | 0.00313256 |
| GO:1903037 | regulation of leukocyte cell-cell adhesion | 0.00420426 |
| GO:0032872 | regulation of stress-activated MAPK cascade | 0.00420426 |
| GO:0070302 | regulation of stress-activated protein kinase signaling cascade | 0.00420426 |
| GO:0051403 | stress-activated MAPK cascade | 0.00420426 |
| GO:0150078 | positive regulation of neuroinflammatory response | 0.00420426 |
| GO:0045071 | negative regulation of viral genome replication | 0.00460612 |
| GO:0071216 | cellular response to biotic stimulus | 0.00462626 |
| GO:0010721 | negative regulation of cell development | 0.00473013 |
| GO:0007259 | receptor signaling pathway via JAK-STAT | 0.00473243 |
| GO:0034763 | negative regulation of transmembrane transport | 0.00487155 |
| GO:0043931 | ossification involved in bone maturation | 0.00487155 |
| GO:0031098 | stress-activated protein kinase signaling cascade | 0.00487155 |
| GO:1902895 | positive regulation of pri-miRNA transcription by RNA polymerase II | 0.0051515 |
| GO:0030278 | regulation of ossification | 0.00520597 |
| GO:0010469 | regulation of signaling receptor activity | 0.00549309 |
| GO:0097696 | receptor signaling pathway via STAT | 0.00549309 |
| GO:0050671 | positive regulation of lymphocyte proliferation | 0.00549309 |
| GO:0009083 | branched-chain amino acid catabolic process | 0.00549309 |
| GO:0007159 | leukocyte cell-cell adhesion | 0.00549309 |
| GO:0042102 | positive regulation of T cell proliferation | 0.00549309 |
| GO:0042116 | macrophage activation | 0.00549309 |
| GO:0032946 | positive regulation of mononuclear cell proliferation | 0.00549309 |
| GO:0046330 | positive regulation of JNK cascade | 0.0056315 |
| GO:0030098 | lymphocyte differentiation | 0.00571505 |
| GO:0042531 | positive regulation of tyrosine phosphorylation of STAT protein | 0.00571505 |
| GO:0070977 | bone maturation | 0.00571505 |
| GO:0032649 | regulation of interferon-gamma production | 0.0057362 |
| GO:0045600 | positive regulation of fat cell differentiation | 0.0057362 |
| GO:1901214 | regulation of neuron death | 0.0057362 |
| GO:0046425 | regulation of receptor signaling pathway via JAK-STAT | 0.00581921 |
| GO:0044321 | response to leptin | 0.00606672 |
| GO:2001257 | regulation of cation channel activity | 0.0061703 |
| GO:0097191 | extrinsic apoptotic signaling pathway | 0.0062281 |
| GO:0009081 | branched-chain amino acid metabolic process | 0.00667786 |
| GO:0051384 | response to glucocorticoid | 0.00667786 |

**KEGG**

| ID | Description | p.adjust |
| --- | --- | --- |
| hsa04668 | TNF signaling pathway | 0.00055437 |
| hsa04657 | IL-17 signaling pathway | 0.00066074 |
| hsa05135 | Yersinia infection | 0.00508087 |
| hsa05142 | Chagas disease | 0.00508087 |
| hsa00280 | Valine, leucine and isoleucine degradation | 0.00578567 |
| hsa05144 | Malaria | 0.00586505 |
| hsa04060 | Cytokine-cytokine receptor interaction | 0.00818988 |
| hsa05134 | Legionellosis | 0.00818988 |
| hsa00640 | Propanoate metabolism | 0.00895506 |
| hsa05323 | Rheumatoid arthritis | 0.00895506 |
| hsa05143 | African trypanosomiasis | 0.01074137 |
| hsa05146 | Amoebiasis | 0.01080637 |
| hsa05171 | Coronavirus disease - COVID-19 | 0.01080637 |
| hsa04620 | Toll-like receptor signaling pathway | 0.01080637 |
| hsa04625 | C-type lectin receptor signaling pathway | 0.01080637 |
| hsa05133 | Pertussis | 0.0153445 |
| hsa05140 | Leishmaniasis | 0.0153445 |
| hsa04310 | Wnt signaling pathway | 0.02118852 |
| hsa04020 | Calcium signaling pathway | 0.03895852 |
| hsa01523 | Antifolate resistance | 0.03895852 |
| hsa04933 | AGE-RAGE signaling pathway in diabetic complications | 0.03895852 |
| hsa05202 | Transcriptional misregulation in cancer | 0.03895852 |
| hsa05321 | Inflammatory bowel disease | 0.03973698 |
| hsa05130 | Pathogenic Escherichia coli infection | 0.03973698 |
| hsa05132 | Salmonella infection | 0.03973698 |

**Supplemental Table 6. Characteristics of the study population in Fecal Metagenomic Sequencing Analysis.**

|  | LBP+FR  (n= 12) | LBP  (n= 12) | HC  (n= 12) | *P*-value |
| --- | --- | --- | --- | --- |
| Age (years) | 44.83 ± 7.1 | 46.25 ± 7.6 | 41.42 ± 10.1 | Ns |
| Gender（F/M） | 6 / 6 | 6 / 6 | 5 / 7 | Ns |
| BMI, mean (Kg/m^2^) | 22.28 ± 2.01 | 22.92 ± 2.13 | 23.17 ± 2.37 | Ns |
| VAS (1-10) | 5.83 ± 1.86 | 3.42 ± 1.85 | ~ | 0.0058 |
| ODI (%) | 49 ± 18.4% | 29.6 ± 15.2% | ~ | 0.013 |
| Total cholesterol  level（mmol/L） | 4.34 ± 0.88 | 4.04 ± 0.8 | 4.21 ± 0.53 | Ns |
| Total triglycerides level（mmol/L） | 1.26 ± 0.56 | 0.99 ± 0.39 | 1.17 ± 0.42 | Ns |
| Diet | Ad libitum diet | Ad libitum diet | Ad libitum diet |  |

**Supplemental Table 7.** **Characteristics of the study population in Faecal Metabolome Analysis.**

|  | LBP+FR  (n= 40) | LBP  (n= 40) | HC  (n= 30) | *P*-value |
| --- | --- | --- | --- | --- |
| Age (years) | 45.18 ± 6.1 | 46.68 ± 8.85 | 41.67 ± 9.52 | Ns |
| Gender（F/M） | 20 / 20 | 22 / 18 | 13 / 17 | Ns |
| BMI, mean (Kg/m^2^) | 21.76 ± 5.81 | 23.72 ± 2.43 | 23.02 ± 2.12 | Ns |
| VAS (1-10) | 5.49 ± 1.99 | 4.45 ± 2.14 | ~ | 0.022 |
| ODI (%) | 46.9 ± 19.7% | 38.3 ± 18.5% | ~ | 0.0314 |
| Total cholesterol  level（mmol/L） | 4.63 ± 1.08 | 4.51 ± 0.99 | 4.48 ± 0.73 | Ns |
| Total triglycerides level（mmol/L） | 1.4 ± 0.79 | 1.48 ± 0。94 | 1.44 ± 0.75 | Ns |
| Diet | Ad libitum diet | Ad libitum diet | Ad libitum diet |  |
